# Phylogenomic Analysis of the Gammaproteobacterial Methanotrophs (Order *Methylococcales*) Calls for the Reclassification of Members at the Genus and Species Levels

**DOI:** 10.3389/fmicb.2018.03162

**Published:** 2018-12-19

**Authors:** Fabini D. Orata, Jan P. Meier-Kolthoff, Dominic Sauvageau, Lisa Y. Stein

**Affiliations:** ^1^Department of Chemical and Materials Engineering, University of Alberta, Edmonton, AB, Canada; ^2^Department of Biological Sciences, University of Alberta, Edmonton, AB, Canada; ^3^Department of Microorganisms, Leibniz Institute DSMZ – German Collection of Microorganisms and Cell Cultures, Braunschweig, Germany

**Keywords:** *Gammaproteobacteria*, *Methylococcales*, methanotroph, *Methylotuvimicrobium* gen. nov., genome BLAST distance phylogeny, digital DNA–DNA hybridization, average nucleotide identity, average amino acid identity

## Abstract

The order *Methylococcales* constitutes the methanotrophs – bacteria that can metabolize methane, a potent greenhouse gas, as their sole source of energy. These bacteria are significant players in the global carbon cycle and can produce value-added products from methane, such as biopolymers, biofuels, and single-cell proteins for animal feed, among others. Previous studies using single-gene phylogenies have shown inconsistencies in the currently established taxonomic structure of this group. This study aimed to determine and resolve these issues by using whole-genome sequence analyses. Phylogenomic analysis and the use of similarity indexes for genomic comparisons – average amino acid identity, digital DNA–DNA hybridization (dDDH), and average nucleotide identity (ANI) – were performed on 91 *Methylococcales* genomes. Results suggest the reclassification of members at the genus and species levels. Firstly, to resolve polyphyly of the genus *Methylomicrobium*, *Methylomicrobium alcaliphilum*, “*Methylomicrobium buryatense*,” *Methylomicrobium japanense*, *Methylomicrobium kenyense*, and *Methylomicrobium pelagicum* are reclassified to a newly proposed genus, *Methylotuvimicrobium* gen. nov.; they are therefore renamed to *Methylotuvimicrobium alcaliphilum* comb. nov., “*Methylotuvimicrobium buryatense*” comb. nov., *Methylotuvimicrobium japanense* comb. nov., *Methylotuvimicrobium kenyense* comb. nov., and *Methylotuvimicrobium pelagicum* comb. nov., respectively. Secondly, due to the phylogenetic affinity and phenotypic similarities of *Methylosarcina lacus* with *Methylomicrobium agile* and *Methylomicrobium album*, the reclassification of the former species to *Methylomicrobium lacus* comb. nov. is proposed. Thirdly, using established same-species delineation thresholds (70% dDDH and 95% ANI), *Methylobacter whittenburyi* is proposed to be a later heterotypic synonym of *Methylobacter marinus* (89% dDDH and 99% ANI). Also, the effectively but not validly published “*Methylomonas denitrificans*” was identified as *Methylomonas methanica* (92% dDDH and 100% ANI), indicating that the former is a later heterotypic synonym of the latter. Lastly, strains MC09, R-45363, and R-45371, currently identified as *M. methanica*, each represent a putative novel species of the genus *Methylomonas* (21–35% dDDH and 74–88% ANI against *M. methanica*) and were reclassified as *Methylomonas* sp. strains. It is imperative to resolve taxonomic inconsistencies within this group, first and foremost, to avoid confusion with ecological and evolutionary interpretations in subsequent studies.

## Introduction

Methanotrophs are microorganisms that can metabolize methane as their sole source of energy (Whittenbury et al., 1970; Bowman, 2018). They play a major role in the global carbon cycle (Cicerone and Oremland, 1988) and thrive in diverse ecosystems that have an influx of methane, including freshwater and marine sediments, wetlands, coal mine drainage waters, wastewater, groundwater, sewage sludge, most soils, and natural gas reserves (Hanson and Hanson, 1996; Knief, 2015; Bowman, 2018). Methane is 28 times more potent as a heat-trapping greenhouse gas than carbon dioxide (Edenhofer et al., 2014) and is a common low-value industrial by-product (Strong et al., 2015). On the other hand, this industrial waste can be converted by methanotrophs into value-added products such as biopolymers, biofuels, single-cell proteins for animal feed and human food, and nutrients for growth media, among others (Strong et al., 2015; Kalyuzhnaya and Xing, 2018).

Methanotrophs with validly published names belong to two classes, *Gammaproteobacteria* (also termed Type I and Type X) and *Alphaproteobacteria* (also termed Type II) (Bowman, 2018). Gammaproteobacterial methanotrophs belong to the order *Methylococcales*, which constitutes the families *Methylococcaceae*, *Methylothermaceae*, and *Crenotrichaceae*, currently including 42 species with validly published names from 19 genera (Parte, 2014). Most of the identified isolates belong to the family *Methylococcaceae*, which consists of 16 genera and 39 species. Family *Methylothermaceae* consists of two genera and two species, and family *Crenotrichaceae* consists of a single genus and species (Parte, 2014). These taxa were circumscribed mainly based on phylogeny of 16S rRNA gene sequences (Bowman, 2018). The oxidation of methane to methanol is performed by the particulate methane monooxygenase (pMMO), present in most methanotrophs, and the PmoA subunit has also been widely used in phylogenetic analyses (Knief, 2015; Bowman, 2018). Taxonomy within this order has been questioned and revised significantly (Bowman et al., 1993, 1995; Bowman, 2018). In some cases, 16S rRNA and PmoA phylogenies are not congruent and certain genera appear as polyphyletic (e.g., *Methylobacter* and *Methylomicrobium*) or paraphyletic (e.g., *Methylosarcina*) (Knief, 2015). A group or taxon is polyphyletic if they are derived from more than one common ancestor; it is paraphyletic if they are derived from a common ancestor, but the taxon does not include all descendants of that common ancestor. These are in contrast to a monophyletic taxon, which is composed of all descendants of a single common ancestor. The monophyletic nature of members in a phylogenetic tree is the main criterion for defining a taxon (Rosselló-Móra and Amann, 2001).

With the advent of next-generation sequencing, phylogeny using whole-genome sequences, as opposed to single genes, has become an important tool for the delineation of prokaryotic taxa and clarification of taxonomic inconsistencies (Chun and Rainey, 2014; Garrity, 2016; Chun et al., 2018; Parks et al., 2018). Recent studies of the phyla *Actinobacteria* and *Bacteroidetes* mainly used a genome-based phylogeny to reclassify organisms at various taxonomic ranks, despite inconsistencies in phenotypic information (Hahnke et al., 2016; Nouioui et al., 2018). Although several major features are still distinctive between Type I and Type II methanotrophs (e.g., one-carbon assimilation pathway, type of intracytoplasmic membrane arrangement, etc.), the characterization of several new genera and species made initially distinct traits no longer indicative for one or the other type (e.g., signature fatty acids, formation of resting stages, optimum growth temperature, etc.) (Knief, 2015). The availability of whole-genome sequences has also provided defined delineation standards through genomic comparisons, more so for species than higher ranks (Garrity, 2016). Experimental DNA–DNA hybridization (DDH) is now being replaced by *in silico* (digital) DDH (dDDH) (Auch et al., 2010; Meier-Kolthoff et al., 2013), yet still maintaining the cut-off of 70% hybridization for two genomes to belong to the same species (Goris et al., 2007). Similarly, average nucleotide identity (ANI) also measures the nucleotide-level similarity between two genomes (Richter and Rosselló-Móra, 2009), which represent the same species if they have at least 95% ANI (Goris et al., 2007). The use of ANI had uncovered high levels of genomic similarity between methanotrophic species *Methylomicrobium agile* and *Methylomicrobium album* (Kalyuzhnaya, 2016a), as well as *Methylobacter marinus* and *Methylobacter whittenburyi* (Collins et al., 2017), therefore questioning their identities. Although not as widely used, average amino acid identity (AAI) (Konstantinidis and Tiedje, 2005) and the percentage of conserved proteins (POCP) (Qin et al., 2014), which both measure amino acid-level genomic similarity between protein-coding regions, have been proposed to delineate organisms at the genus level.

This study aimed to establish a whole-genome phylogeny for currently available *Methylococcales* genomes, supplemented with genome-based similarity indexes to determine and resolve taxonomic inconsistencies within this group of microorganisms.

## Materials and Methods

### 16S rRNA, PmoA, and Whole-Genome Sequences Used in This Study

Nucleotide or amino acid sequences of the 16S rRNA and *pmoA* genes from 49 type and representative strains of *Methylococcales*, including species with effectively but not validly published names, were obtained from the NCBI GenBank (Benson et al., 2018) or MicroScope (Médigue et al., 2017) databases (Supplementary Table S1). Additionally, a total of 91 whole-genome sequences were obtained from the NCBI Microbial Genomes (Tatusova et al., 2014) or MicroScope (Médigue et al., 2017) databases, including 86, 3, and 2 genomes previously identified to belong to *Methylococcaceae*, *Crenotrichaceae*, and *Methylothermaceae*, respectively (Supplementary Table S2). Annotated plasmid sequences, if present, were excluded from any analysis. An additional set of sequences from two species belonging to the genus *Methylosinus* (Type II methanotroph) was chosen as outgroup for phylogenetic analyses.

### 16S rRNA and PmoA Phylogenies

The 16S rRNA and PmoA sequences were aligned with MAFFT 1.3.7 (Katoh and Standley, 2013) in Geneious 11.1.3 (Kearse et al., 2012). Poorly aligned positions were eliminated using GBlocks 0.91b (Castresana, 2000). The final alignments, with 1,469 nucleotide positions for 16S rRNA and 243 amino acid positions for PmoA, were used to reconstruct maximum-likelihood phylogenetic trees with RAxML 8.2.12 (Stamatakis, 2014). The GTR (general time reversible) nucleotide substitution model or WAG (Whelan and Goldman) amino acid substitution model and the gamma model of rate heterogeneity were used. Robustness of branching was estimated with 100 bootstrap replicates. Nodes with 50% or less bootstrap support were collapsed to polytomies using TreeCollapserCL 4.0 (Hodcroft, 2016). The trees and support values were visualized using iTOL 4.2 (Letunic and Bork, 2016) or FigTree 1.4.3 (Rambaut, 2007).

### Whole-Genome Phylogeny

The high-throughput version (Meier-Kolthoff et al., 2014a) of the Genome BLAST Distance Phylogeny (GBDP) approach (Henz et al., 2005; Meier-Kolthoff et al., 2013) was used to infer phylogenies from the genome sequences (restricted to coding regions) in conjunction with BLAST+ 2.2.3 (Camacho et al., 2009) in BLASTN mode with default parameters except for an *E*-value filter of 10^-8^ (Meier-Kolthoff et al., 2014a). The greedy-with-trimming GBDP algorithm was applied in conjunction with formula *d_5_* and subjected to 100 pseudo-bootstrap replicates (Meier-Kolthoff et al., 2013, 2014a). FastME 2.1.4 (Lefort et al., 2015) was used to infer phylogenetic trees from the original and pseudo-bootstrapped intergenomic distance matrices. The tree and support values were visualized using iTOL 4.2 (Letunic and Bork, 2016) or FigTree 1.4.3 (Rambaut, 2007).

### Genome-Based Similarity Indexes for Genus Delineation

For the clarification of genus affiliations, AAI (Konstantinidis and Tiedje, 2005) and POCP (Qin et al., 2014) were used for amino acid-level comparisons for every pairwise combination of genomes. AAI was determined by calculating the mean protein sequence similarity of all protein-coding genes shared between strains (Konstantinidis and Tiedje, 2005). This was done with CompareM 0.0.21 (Parks, 2017), which employed Prodigal 2.6.3 for gene calling (Hyatt et al., 2010) and DIAMOND 0.9.19 to perform sequence similarity searches (Buchfink et al., 2015), using default BLASTP parameters (i.e., 10^-5^ *E*-value, 30% sequence identity cut-off, and ≥70% alignment length) to define bidirectional best BLAST hits between genomes. Also, using CompareM 0.0.21 (Parks, 2017), the number of orthologous genes shared between two genomes was determined. This was subsequently used to calculate POCP using the formula [(2 × S)/(T1+T2)] × 100%, where S represents the number of genes shared between genomes and T1 and T2 represent the total number of proteins in the two genomes being compared (Qin et al., 2014).

### Genome-Based Similarity Indexes for Species Delineation

For the clarification of species affiliations, dDDH (Auch et al., 2010; Meier-Kolthoff et al., 2013) and ANI (Goris et al., 2007) were used for nucleotide-level comparisons for every pairwise combination of genomes. dDDH was calculated using the recommended settings (formula 2) of the Genome-to-Genome Distance Calculator 2.1 (Auch et al., 2010; Meier-Kolthoff et al., 2013). ANI was calculated as described by Goris et al. (2007) and using default parameters in JSpecies 1.2.1 (Richter and Rosselló-Móra, 2009). Briefly, the query genome was cut into 1,020 bp fragments, and these were used to search against the reference genome using BLASTN with a sequence identity cut-off and an alignment length minimum as above. The query and reference genomes were then reversed. The bidirectional best BLAST hits for each fragment were reported as the average percent identity from all comparisons. Two genomes belonging to the same species would have a dDDH of at least 70%, which corresponds to at least 95% ANI (Goris et al., 2007; Auch et al., 2010; Meier-Kolthoff et al., 2013).

### Selection of Phenotypes to Support Reclassifications

In cases were phenotypic information was needed to clarify taxon affiliation, data on several phenotypic characteristics commonly tested between organisms were collected from the original isolation papers and the *Bergey’s Manual of Systematics of Archaea and Bacteria* (Table 1; Bowman, 2016a,b; Hirayama, 2016; Kalyuzhnaya, 2016a,b; Collins et al., 2017; Bowman, 2018). Data missing from isolation papers and information on isolates that are not type strains were retrieved from other references as stipulated.

**Table 1:** Phenotypic characteristics of member species of *Methylomicrobium*, *Methylotuvimicrobium* gen. nov., and *Methylosarcina*.

Genus	*Methylomicrobium*			*Methylotuvimicrobium* gen. nov.			*Methylosarcina*	
Species	*Methylomicrobium agile*	*Methylomicrobium album*	*Methylomicrobium lacus* c.n.	*Methylotuvimicrobium alcaliphilum* c.n.	“*Methylotuvimicrobium buryatense*” c.n.	*Methylotuvimicrobium japanense* c.n.	*Methylotuvimicrobium kenyense* c.n.	*Methylotuvimicrobium pelagicum* c.n.	*Methylosarcina fibrata*	*Methylosarcina quisquiliarum*
Former name	NA	NA	*Methylosarcina lacus*	*Methylomicrobium alcaliphilum*	“*Methylomicrobium buryatense*”	*Methylomicrobium japanense*	*Methylomicrobium kenyense*	*Methylomicrobium pelagicum*	NA	NA
Type strain	ATCC 35068	BG8	LW14	20Z	5B	NI	AMO1	AA-23	AML-C10	AML-D4
Type species	Yes	No	No	Yes	No	No	No	No	Yes	No
**Characteristics**		
Pigmentation	W to SL	W to SL	W to SL	W to SL	W to SL	W to SL	W to SL	W to SL	LB	LB
Motility	+	+	–	+	+	+	+	+	+	+
Cyst formation	–	–	–	–	–	–	–	–	+	+
Desiccation resistance	–	–	–	+	+	–	–	–	–	–
Growth occurs with 3.0% NaCl	–	–	NR	+	+	+	+	+	–	–
Temp. growth, range (°C)	10–37	10–37	4–35	NR	4–45	NR	NR	10–30	22–37	22–32
Temp. growth, optimum (°C)	25–30	25–30	28–30	NR	28–30	15–37	NR	20–25	NR	NR
Growth at 37°C	+	V	–	+	+	+	–	–	+	–
Growth at 45°C	–	–	NR	–	+	–	–	–	NR	NR
Heat resistance (80°C)	–	–	–	+	+	–	–	–	–	–
pH growth, range	6–9	6–9	4–7	6.5–9.5	6–11	NR	9–10.5	6–8.5	5.0–9.0	5.5–9.0
pH growth, optimum	7	7	5.5–6.5	9	8.5–9.5	8.1	10	7	NR	NR
pMMO	+	+	+	+	+	+	+	+	+	+
sMMO	–	–	–	–	+	+	–	–	–	–
Main fatty acid	C_16:1_ ω5t	C_16:1_ ω5t	C_16:1_ ω8c	C_16:1_ ω7c	C_16:1_ ω7c	C_16:1_	NR	C_16:1_ ω5t	C_16:1_ ω5t	C_16:1_ ω7c
DNA G+C content (mol%)	58.1–59.6	54.4–56.3	52–54.7	48–49	48–49	49	50.2	48.5	53.9–54.3	54–54.6

*Data obtained from Bowman et al. (1995), Wise et al. (2001), Kalyuzhnaya et al. (2005), Bowman (2016a), and Kalyuzhnaya (2016a,b). c.n., combinatio nova; NA, not applicable; +, present/tested positive; –, absent/tested negative; V, variable among strains; W to SL, white to slightly cream; LB, light brown; NR, not reported; pMMO, particulate methane monooxygenase; sMMO, soluble methane monooxygenase.*

## Results and Discussion

**FIGURE 1:**
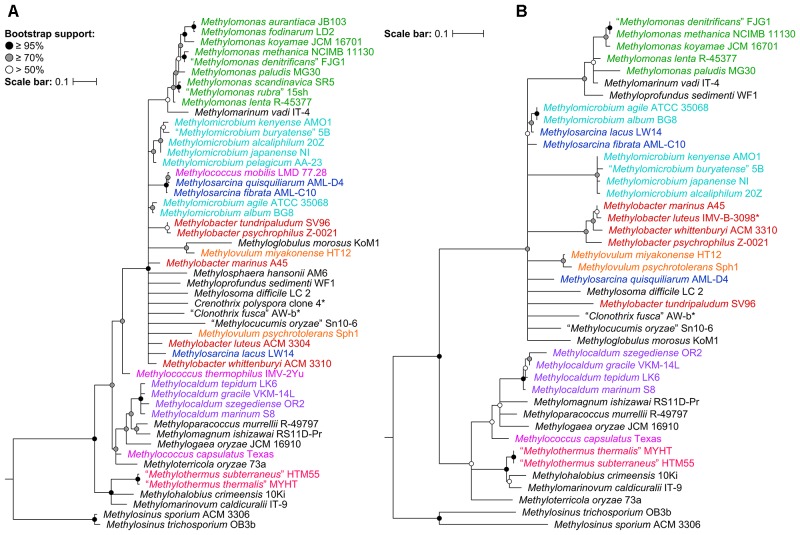
The phylogenetic relationship of *Methylococcales* type and representative strains based on **(A)** 16S rRNA and **(B)** PmoA sequences. The maximum-likelihood trees were constructed from the alignments of **(A)** 16S rRNA (1,469 nucleotide positions) and **(B)** PmoA (243 amino acid positions) sequences. *Methylosinus* was chosen as outgroup. Nodes with 50% or less bootstrap support are collapsed. Bootstrap support is indicated on the nodes as black (≥95%), gray (≥70%), or white (>50%) circles. The scale bars represent **(A)** nucleotide or **(B)** amino acid substitutions per site. Genera with at least two members are colored; genera with single members (and outgroup) are black. Asterisks indicate non-type strains.

To determine the impact of using the 16S rRNA and *pmoA* genes as main molecular markers for defining new methanotrophic taxa, phylogenetic analyses were performed using sequences from all 49 currently known type and representative strains belonging to the order *Methylococcales*, including those with effectively but not validly published species names (Supplementary Table S1). The phylogenetic trees show low bootstrap support overall (Figure 1 and Supplementary Figure S1). When nodes with bootstrap support below 50% are collapsed, the poorly resolved tree backbones are evident (Figure 1). One should take caution when interpreting relationships from these trees, always keeping in mind that the taxon of interest should correspond to a highly supported monophyletic clade. It is also important to note that the use of these gene markers alone should not describe a taxon, but they can provide the first indication that an isolate could belong to a novel or already existing taxon (Tindall et al., 2010).

**FIGURE 2:**
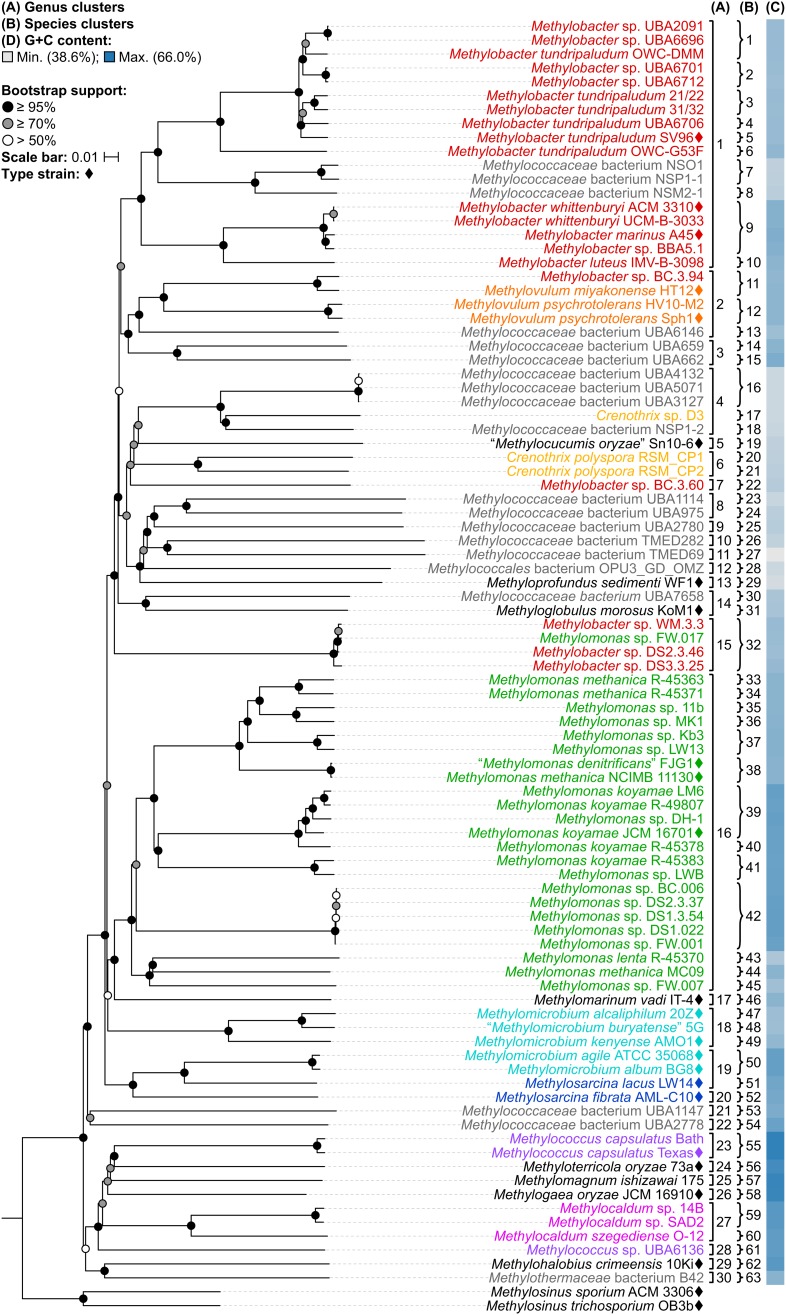
The *Methylococcales* phylogenomic tree inferred with GBDP. The tree was inferred with FastME from GBDP distances calculated from the genome sequences restricted to coding regions. *Methylosinus* was chosen as outgroup. Pseudo-bootstrap support is indicated on the nodes as black (≥95%), gray (≥70%), or white (>50%) circles. The branches are scaled in terms of log-transformed intergenomic distances (GBDP formula *d_5_*). Genera with at least two members are colored; genera with single members (and outgroup) are black; genomes with no genus designation are gray. Diamonds indicate type strains. Inferred clusters are for **(A)** genus, denoted by numbers and brackets and **(B)** species, denoted by numbers and braces. **(C)** Percent G+C content of genome sequences.

A whole-genome-based phylogenetic tree was then reconstructed from 91 *Methylococcales* genomes via the GBDP approach (Henz et al., 2005; Meier-Kolthoff et al., 2013). The inferred tree was used to assess the monophyletic status of members supposedly belonging to the same taxon. Unlike the 16S rRNA and PmoA trees (Figure 1 and Supplementary Figure S1), the genome phylogeny revealed robust bootstrap support with most branches having maximum support (Figure 2). This highlighted apparent taxonomic inconsistencies, but also allowed for the confident establishment of relationships within *Methylococcales*.

### Taxonomic Structure at the Family Level Within *Methylococcales*

The reconstructed genome phylogeny reveals three lineages of gammaproteobacterial methanotrophs (Figure 2). This is because the clade consisting of the genera *Methylococcus*, *Methyloterricola*, *Methylomagnum*, *Methylogaea*, and *Methylocaldum* (i.e., Type X methanotrophs) is not monophyletic with the rest of family *Methylococcaceae*. These three lineages were also shown by the Genome Taxonomy Database (GTDB) phylogeny reconstructed from 120 ubiquitous single-copy protein-coding genes (Parks et al., 2018). As such, Parks et al. (2018) proposed the transference of the majority of *Methylococcaceae* members to a different family. The three family lineages were therefore named as “*Methylomonadaceae*,” *Methylococcaceae*, and *Methylothermaceae* (Supplementary Figure S2; Parks et al., 2018), corresponding to Type Ia, Ib (or X), and Ic, respectively (Knief, 2015; Bowman, 2016a).

The Type X and *Methylothermaceae* clades appear monophyletic in the genome-based tree (Figure 2), and it can be argued that members of these clades could be merged into one family. Members of these lineages are mesophilic to thermophilic and form dessication-resistant *Azotobacter*-type cysts, unlike members of “*Methylomonadaceae*” which are psychrophilic to mesophilic and form non-desiccation-resistant “immature” *Azotobacter*-type cysts (Bowman, 2016a). On the other hand, members of *Methylothermaceae* can be distinguished from *Methylococcaceae* by their abundance of C_18:1_ fatty acids (Hirayama et al., 2014; Hirayama, 2016), a typical characteristic of Type II methanotrophs (Hanson and Hanson, 1996). *Methylothermaceae* consists of three genera, “*Methylothermus*,” *Methylomarinovum*, and *Methylohalobius*, but only the latter has a genome-sequenced type strain. Thus, reclassification, if warranted, is premature at this point without complete genomic information.

Despite the lack of a cultivated representative, *Crenothrix polyspora* is a validly published species name (Ad Hoc Committee of the Judicial Commission of the ICSB, 1989). The species is listed under family *Crenotrichaceae* in the List of Prokaryotic Names with Standing in Nomenclature (Parte, 2014) but has been transferred to *Methylococcaceae* in the most recent edition of the *Bergey’s Manual of Systematics of Archaea and Bacteria* (Bowman, 2016a). Although *C. polyspora* is multicellular and filamentous, its assignment to *Methylococcaceae* is consistent with the occurrence of intracytoplasmic membrane stacks and their arrangement as vesicular discs (Völker et al., 1977; Stoecker et al., 2006), as well as the placement of the species within the family based on 16S rRNA phylogeny (Figure 1A and Supplementary Figure S1A; Völker et al., 1977; Stoecker et al., 2006; Vigliotta et al., 2007; Knief, 2015; Bowman, 2016a; Oswald et al., 2017). Indeed, the genome phylogeny shows the three *Crenothrix* genomes (*C. polyspora* RSM_CP1 and RSM_CP2 and *Crenothrix* sp. D3) positioned well within *Methylococcaceae* with high bootstrap support (Figure 2). To our knowledge, this is the first confirmation by whole-genome phylogeny of *Crenothrix* belonging to *Methylococcaceae*. Additionally, these three genomes are not monophyletic, and the consequence of this polyphyly in terms of genus nomenclature is discussed below.

A fourth family, “*Cycloclasticaceae*,” was introduced by the GTDB taxonomy as a member of order *Methylococcales* (Parks et al., 2018). This family includes the species *Cycloclasticus pugetii*, a gammaproteobacterium that does not use methane or methanol but is instead capable of growing on aromatic hydrocarbons as sole sources of organic carbon (Dyksterhouse et al., 1995). The genus *Cycloclasticus* is currently a member of the family *Piscirickettsiaceae* (Parte, 2014; Geiselbrecht, 2015). Interestingly, Bowman (2016a, 2018) noted the paraphyly of *Methylococcales* to a number of other taxa of the class *Gammaproteobacteria* (e.g., *Cycloclasticus* and *Methylophaga*) based on 16S rRNA phylogeny. This raises the possible inclusion of non-methanotrophic bacteria into *Methylococcales*, which currently only includes methanotrophs (Bowman, 2018), and the potential reorganization of taxonomic structure at the family and higher levels. The presence of non-methanotrophic members in the same family has been identified for the Type II methanotrophs (i.e., *Beijerinckiaceae* and *Methylocystaceae* of the order *Rhizobiales*) (Parte, 2014). Reorganization and reclassification of this level warrants a larger-scale genome sequencing effort, especially of unsequenced type strains within *Methylococcales* and all closely related taxa. Such effort in sequencing the genomes of type strains and making them publicly available is underway, such as the massive Genomic Encyclopedia of *Bacteria* and *Archaea* sequencing project (Wu et al., 2009; Kyrpides et al., 2014; Whitman et al., 2015; Mukherjee et al., 2017).

### Use of POCP vs. AAI for the Delineation of Genera

Taxonomic reorganization is required at the genus level because of several polyphyletic (e.g., *Methylobacter*, *Crenothrix*, *Methylomonas*, *Methylomicrobium*, and *Methylococcus*) and paraphyletic (e.g., *Methylovulum* and *Methylosarcina*) genera (Figure 2). Reorganization requires setting criteria for the amount of diversity allowed within genera, keeping in mind the use of a genome-based phylogeny as a primary guideline to identify clades that (i) are monophyletic (Rosselló-Móra and Amann, 2001) and (ii) would require the fewest number of changes from the current taxonomy. Here, the viability of genome-based similarity indexes, POCP (Qin et al., 2014) and AAI (Konstantinidis and Tiedje, 2005), as supplements to genome phylogeny to infer genera were examined.

**FIGURE 3:**
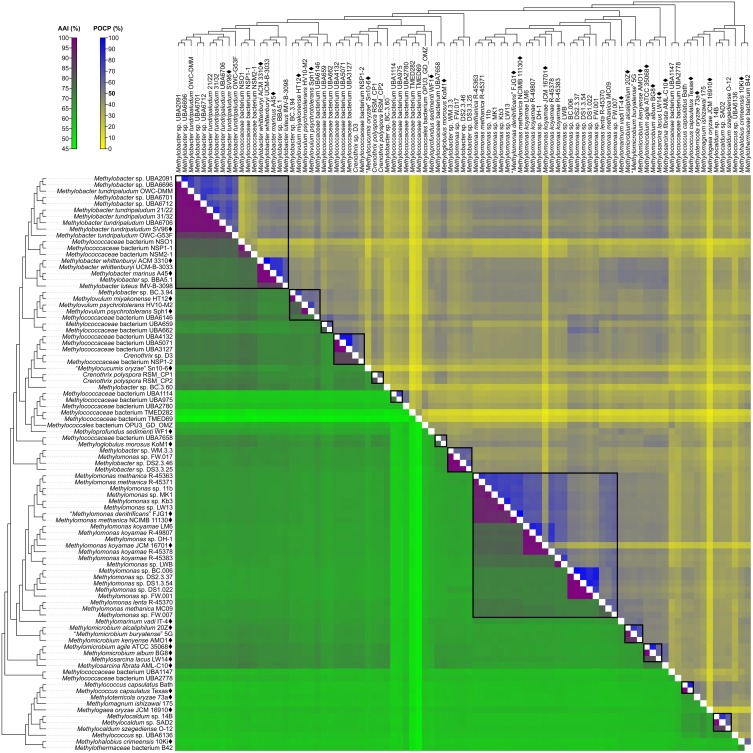
AAI and POCP from pairwise whole-genome comparisons. The heat map shows AAI and POCP values between genomes, along with the tree cladogram to show relationships. Boxed regions indicate inferred genus clusters with at least two members based on AAI comparisons, as well as monophyly in the genome-based phylogeny. POCP was not used to infer genera.

A fixed genus boundary of 50% POCP has been proposed (Qin et al., 2014). Applying this boundary shows several pairwise comparisons of at least 50% POCP (Figure 3 and Supplementary Table S3) that would automatically violate the monophyly rule for taxon delineation (Rosselló-Móra and Amann, 2001). Taking into consideration comparisons with at least 50% POCP that also exhibited monophyly in the genome phylogeny, inferred genus-level clusters would require splitting previously described species from the same genus into different genera (Supplementary Figure S3). For example, the inferred *Methylobacter* clade, the largest monophyletic clade formed by the majority of *Methylobacter* strains (Figure 2), would be split into five genera and separate type strains *Methylobacter tundripaludum* SV96^T^ (Wartiainen et al., 2006) from *M. whittenburyi* ACM 3310^T^ and *M. marinus* A45^T^ (Bowman et al., 1993; Supplementary Figure S3). The same could be said for the inferred *Methylomonas* clade, the largest monophyletic clade formed by the majority of *Methylomonas* strains (Figure 2), which would be split into seven genera. It would separate *Methylomonas methanica* NCIMB 11130^T^ (Bowman, 2016b) and “*Methylomonas denitrificans*” FJG1^T^ (Kits et al., 2015) from *Methylomonas koyamae* JCM 16701^T^ (Ogiso et al., 2012; Supplementary Figure S3). On the other hand, this POCP threshold would merge *Methylococcus capsulatus* Texas^T^ (Foster and Davis, 1966) and *Methyloterricola oryzae* 73a^T^ (Frindte et al., 2017) into the same genus. In this context, the 50% POCP boundary is not an appropriate metric to delineate genera within *Methylococcaceae*. The use of the POCP has, similarly, been shown to be ineffective in delineating genera within the families *Bacillaceae* (Aliyu et al., 2016), *Burkholderiaceae* (Lopes-Santos et al., 2017), *Neisseriaceae* (Li et al., 2017), and *Rhodobacteraceae* (Wirth and Whitman, 2018), among others.

Another tool proposed for delimiting taxonomic ranks at the genus level is AAI (Konstantinidis and Tiedje, 2005). However, prokaryotic taxa exhibit a range of AAI values, making distinct boundaries difficult to define (Konstantinidis and Tiedje, 2005; Luo et al., 2014). AAI comparisons by Luo et al. (2014) of related but different genera typically ranged from 60 to 80%. We determined pairwise AAI values for the 91 *Methylococcales* genomes investigated (Figure 3 and Supplementary Table S3). Over 70% of comparisons had at least 60% AAI, which would result in the collapse of the majority of genomes into a single genus. To minimize changes to the current nomenclature, this inferred lower AAI boundary (Luo et al., 2014) cannot be applied to discriminate genera. We then looked at the AAI range of the two most well-represented monophyletic clades, *Methylobacter* and *Methylomonas* (genus clusters 1 and 16, respectively) (Figure 2). In a previous study with a limited number of five to six genomes, the AAI ranges for these genera were determined to be 70–95% and 75–90%, respectively (Skennerton et al., 2015). In our analysis, the AAI values obtained ranged from 74 to 100% and 71 to 100% from 18 and 23 genomes, respectively (Supplementary Table S3 and Supplementary Figure S3). For the *Methylobacter* clade, going below 74% AAI would include several genomes (*Methylobacter* sp. BC.3.94, *Methylovulum miyakonense* HT12^T^, and *Methylococcaceae* bacterium UBA6146) in the genus that would result in paraphyly, which should be avoided.

The use of 71% AAI as the lower genus limit resulted in 30 inferred monophyletic genera that maintained most of the current classifications for the identified genomes (Figure 2). The AAI ranges within the different inferred genera varied (Supplementary Table S3 and Supplementary Figure S3). The clades containing *Methylovulum* (genus cluster 2), *Crenothrix* sp. D3 (genus cluster 4), and *Methylocaldum* (genus cluster 27) (Figure 2) had AAI ranges of 73–100%, 84–100%, and 84–100%, respectively. The variable AAI ranges among the inferred genus clades may be due to a lack of representation of some genera, but variations in AAI can be expected since different prokaryotic taxa, even those that are closely related, can evolve at different rates due to differences in responses to evolutionary and ecological processes (Ramette and Tiedje, 2007). These AAI ranges may eventually change as more genomes from identified isolates become available in the future, allowing for an even better resolution of individual genus-level clades.

As mentioned previously, the three *Crenothrix* genomes are not monophyletic, with *Crenothrix* sp. D3 (Oswald et al., 2017) instead exhibiting affinity with “*Methylocucumis oryzae*” Sn10-6 (Pandit et al., 2018; Figure 2). The initial assignment of genome D3 to *Crenothrix* was ambiguous since it is separate from *C. polyspora* in the 16S rRNA phylogeny and 60% AAI was used for genus prediction (Oswald et al., 2017). Genome D3 is monophyletic with several other genomes only designated as *Methylococcaceae* bacterium, UBA4132, UBA5071, UBA3127, and NSP1-2 (genus cluster 4). As inferred by the GTDB taxonomy (Parks et al., 2018), this clade is separate from *C. polyspora* RSM_CP1 and RSM_CP2 (genus cluster 6). *Crenothrix* sp. D3 is thus renamed to *Methylococcaceae* bacterium D3, since only one clade can keep the genus name. Genomes RSM_CP1 and RSM_CP2 remained as *Crenothrix* based solely on their phylogenetic affinity with other 16S rRNA genes from *C. polyspora* (Oswald et al., 2017). All sequenced members of these clades are metagenome-assembled genomes (MAGs) obtained from various methane sinks (Oswald et al., 2017; Parks et al., 2017; Smith et al., 2018), so formal genus descriptions cannot be performed until known isolates exhibiting affinity within these clades become available (Tindall et al., 2010).

A similar case was seen with *Methylococcus*, where *M. capsulatus* Texas^T^ and Bath are monophyletic (genus cluster 23) but are positioned separately from *Methylococcus* sp. UBA6136 (genus cluster 28) (Figure 2), a MAG derived from an oil sands tailings pond metagenome (Parks et al., 2017). The latter is renamed *Methylococcaceae* bacterium UBA6136 as it is currently the sole member of a yet to be described genus.

The *Methylovulum* clade (genus cluster 2) consists of type strains *M. miyakonense* HT12^T^ (Iguchi et al., 2011) and *Methylovulum psychrotolerans* Sph1^T^ (Oshkin et al., 2016) and strain HV10-M2 (Mateos-Rivera et al., 2018; Figure 2). This clade also includes *Methylobacter* sp. BC.3.94 and *Methylococcaceae* bacterium UBA6146, both derived from wastewater metagenomes from groundwater (Zhang et al., 2017) and an oil sands tailings pond (Parks et al., 2017), respectively. Accordingly, the latter strains are renamed *Methylovulum* sp. BC.3.94 and *Methylovulum* sp. UBA6146.

The two genomes representing *Methylothermaceae*, *Methylohalobius crimeensis* 10Ki^T^ (Heyer et al., 2005) and *Methylothermaceae* bacterium B42, are monophyletic but likely represent different genera (genus clusters 29 and 30, respectively) (Figure 2), as determined previously by Skennerton et al. (2015) and the GTDB taxonomy (Parks et al., 2018), since AAI between the two is only 69% (Supplementary Table S3).

### Resolving Genus-Level Taxonomic Inconsistencies Between *Methylomicrobium* and *Methylosarcina*

Our whole-genome phylogeny shows the five *Methylomicrobium* strains to be polyphyletic (Figure 2). *M. agile* ATCC 35068^T^ and *M. album* BG8^T^ are separate from *Methylomicrobium alcaliphilum* 20Z^T^, “*Methylomicrobium buryatense*” 5G, and *Methylomicrobium kenyense* AMO1^T^. AAI between these clades is 66–67% and are thus proposed to represent different genera (Supplementary Table S3), consistent with the GTDB taxonomy (Parks et al., 2018). We propose the transference of *M. alcaliphilum*, “*M. buryatense*,” and *M. kenyense* to a genus different from *M. agile* and *M. album*. According to Rule 39a (section 7, chapter 3) of the *International Code of Nomenclature of Bacteria: Bacteriological Code* (Lapage et al., 1992), if a genus is divided into two or more genera, the generic name must be retained for one of them based on name priority by publication date (Rules 23a and 23b, section 5) and/or type designation (Rule 39b, section 7). Therefore, we propose the reclassification of *M. alcaliphilum*, “*M. buryatense*,” and *M. kenyense* to a novel genus, *Methylotuvimicrobium* gen. nov. They should then be renamed *Methylotuvimicrobium alcaliphilum* comb. nov., “*Methylotuvimicrobium buryatense*” comb. nov., and *Methylotuvimicrobium kenyense* comb. nov., respectively, since they were identified (ca. 2001 and 2008) (Kaluzhnaya et al., 2001; Kalyuzhnaya et al., 2008) after *M. agile* and *M. album* (ca. 1995) and since *M. agile* is the type species of the genus (Bowman et al., 1995).

Unfortunately, the genomes of three closely related type strains, “*M. buryatense*” 5B^T^ (Kaluzhnaya et al., 2001), *Methylomicrobium japanense* NI^T^ (Kalyuzhnaya et al., 2008), and *Methylomicrobium pelagicum* AA-23^T^ (Bowman et al., 1995), are not sequenced, although “*M. buryatense*” has a genome-sequenced representative strain (Figure 2). Despite the lack of genome sequences for these strains, several pieces of evidence suggest that they would also likely belong to *Methylotuvimicrobium* gen. nov. Firstly, 16S rRNA and PmoA phylogenies consistently placed these species in a monophyletic clade with *M. alcaliphilum* and *M. kenyense* with high bootstrap support (Figure 1 and Supplementary Figure S1; Kalyuzhnaya et al., 2008; Knief, 2015; Bowman, 2016a). Secondly, experimental DDH supports these species to be more closely related to each other and to “*M. buryatense*” 5G than to *M. agile* and *M. album* (Kalyuzhnaya et al., 2008). Thirdly, members of *Methylotuvimicrobium* gen. nov. are halophilic with most strains also alkaliphilic, which differentiates them from *M. agile* and *M. album* (Table 1). Their G+C content range is 48–50% compared to 52–60% for *Methylomicrobium*. Ideally, this reclassification would warrant *M. pelagicum* comb. nov. type species status of the novel genus. However, type strain AA-23^T^ has been lost from culture collections, and there are no other cultures available to replace/represent this species (Kalyuzhnaya et al., 2008; Kalyuzhnaya, 2016a). Thus, we also propose *M. alcaliphilum* comb. nov. as the type species of *Methylotuvimicrobium* gen. nov. since it is the earliest described species with a validly published name after *M. pelagicum* comb. nov. (Kalyuzhnaya et al., 2008). It is imperative to sequence the genomes of the remaining type strains to confirm their placement within this group in the context of a genome phylogeny. Nonetheless, a description of *Methylotuvimicrobium* gen. nov. and emended descriptions of the species proposed to belong to this genus are included.

Furthermore, the two *Methylosarcina* type strains, *Methylosarcina lacus* LW14^T^ and *Methylosarcina fibrata* AML-C10^T^, are paraphyletic (Figure 2). This was addressed in the GTDB taxonomy by splitting the two species into separate genera (Parks et al., 2018). However, both strains are monophyletic with *M. agile* ATCC 35068^T^ and *M. album* BG8^T^, and AAI comparisons between groups (73–82% AAI) suggest that the two *Methylosarcina* strains could be reclassified to *Methylomicrobium* (Supplementary Table S3). However, with *M. fibrata* as the type species of the genus (Wise et al., 2001), and *Methylosarcina quisquiliarum* not included in our dataset (i.e., genome is not sequenced), it is premature to completely collapse *M. fibrata*, *M. lacus*, and, consequently, *M. quisquiliarum* with *Methylomicrobium* without complete genomic information. Based on 16S rRNA phylogeny, *M. quisquiliarum* is more closely related to *M. fibrata* than *M. lacus* (Figure 1A and Supplementary Figure S1A) (Wise et al., 2001; Kalyuzhnaya et al., 2005, 2008; Knief, 2015; Bowman, 2016a). Unlike *M. fibrata* and *M. quisquiliarum*, *M. lacus* does not form sarcina-like clusters (Kalyuzhnaya et al., 2005; Kalyuzhnaya, 2016b). The former two species also differ from the latter based on pigmentation (light brown as opposed to white to slightly light cream) and their ability to form cysts (Table 1). The absence of pigmentation and inability to form cysts are shared characteristics between *Methylomicrobium* and *Methylotuvimicrobium* gen. nov. In addition, *M. lacus* was shown previously to be more closely related to *M. album* than *M. fibrata* based on whole-genome sequence comparisons (Kalyuzhnaya, 2016b), which is consistent with our findings (Supplementary Table S3). *M. lacus* is psychrotolerant and is able to grow at lower temperatures (<22°C), unlike *M. fibrata* and *M. quisquiliarum* (Table 1). *M. agile* and *M. album* are also capable of growth at temperatures as low as 10°C. Based on genome phylogeny, genomic comparisons, and phenotypic differences, we propose the reclassification of *M. lacus* into the genus *Methylomicrobium* as *Methylomicrobium lacus* comb. nov. Emended descriptions of *Methylomicrobium* and *Methylosarcina* are included.

### Use of dDDH, ANI, and G+C Content for the Delineation of Species

**FIGURE 4:**
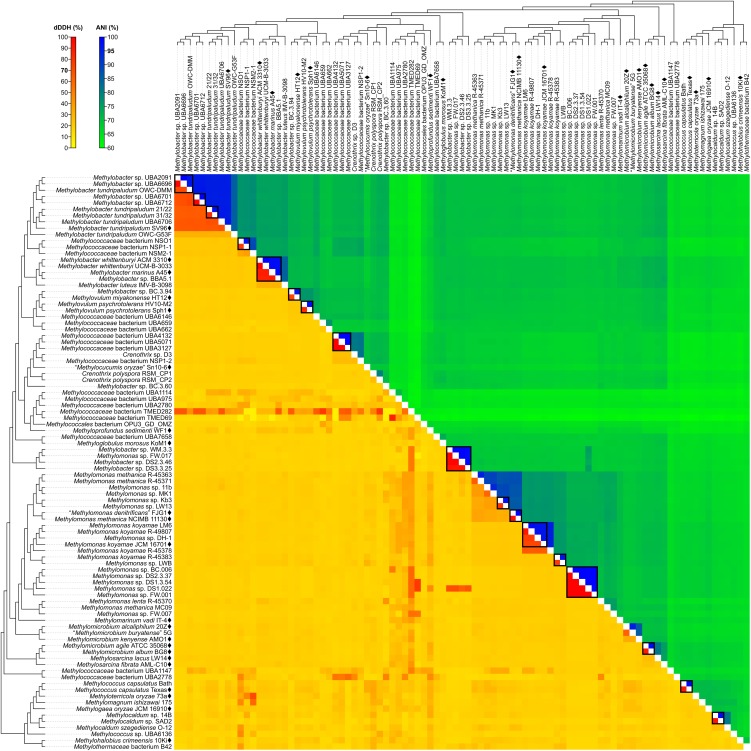
dDDH and ANI from pairwise whole-genome comparisons. The heat map shows dDDH and ANI values between genomes, along with the tree cladogram to show relationships. Boxed regions indicate inferred species clusters with at least two members based on comparisons that are at least 70% dDDH and 95% ANI, as well as monophyly in the genome-based phylogeny.

Although reclassifications at the genus level has resolved most of the polyphyly and paraphyly within *Methylococcales*, misclassifications at the species level also need to be addressed. For this, pairwise nucleotide-level comparisons were performed between genome sequences to determine dDDH (Auch et al., 2010; Meier-Kolthoff et al., 2013) and ANI values (Goris et al., 2007; Figure 4 and Supplementary Table S4). The G+C content from genome sequences was also determined (Supplementary Table S5). Using previously established species delineation standards, two genomes are considered to belong to the same species if they (i) are monophyletic (Rosselló-Móra and Amann, 2001), (ii) have at least 70% dDDH (Auch et al., 2010; Meier-Kolthoff et al., 2013) and 95% ANI (Goris et al., 2007), and (iii) have less than 1% difference in G+C content (Meier-Kolthoff et al., 2014b). Genomic comparisons revealed 63 species clusters (Figure 2).

Several genomes initially identified as *M. tundripaludum* proved to be polyphyletic due to the inclusion of unidentified *Methylobacter* sp. genomes (UBA2091, UBA6696, UBA6701, and UBA6712). dDDH values less than 70% against type strain SV96^T^ (Wartiainen et al., 2006) indicate that none of these genomes can be attributed to *M. tundripaludum* (Supplementary Table S4). Several comparisons within this group, however, showed 95–96% ANI. In cases where ANI is near the threshold for species delineation (i.e., 94–96%), dDDH is proven to be more discriminatory, as demonstrated with *Vibrio cidicii* (Orata et al., 2016) and “*Bradyrhizobium brasilense*” (Martins da Costa et al., 2017), where dDDH values were below the threshold when the species were compared against their closest relatives and ANI was inconclusive. Therefore, the *Methylobacter* genomes were reclassified into several unidentified species (clusters 1–4 and 6) within a known genus (i.e., *Methylobacter*) (Figure 2). Several other species clusters with at least two members and identified only up to the genus level include clusters 7 (*Methylobacter* sp.), 37, 41, 42 (*Methylomonas* sp.), and 59 (*Methylocaldum* sp.). It has been shown previously through comparative genomics that the MAGs of species clusters 7 (*Methylococcaceae* bacterium NSO1 and NSP1-1) and 8 (*Methylococcaceae* bacterium NSM2-1) are part of the *Methylobacter* clade (Smith et al., 2018) and strains Kb3 and LW13 (Kalyuzhnaya et al., 2015) of cluster 37 represent a putative novel species of the genus *Methylomonas* (Rahalkar and Pandit, 2018).

Genus cluster 6 contains the MAGs *C. polyspora* RSM_CP1 and RSM_CP2 (Figure 2). Since both genomes represent different species (clusters 20 and 21, respectively, based on 26% dDDH and 80% ANI) (Supplementary Table S4), only one of the strains can keep the species name. We retained the species designation to strain RSM_CP1 based solely on its phylogenetic affinity with previously sequenced 16S rRNA genes from *C. polyspora* (Oswald et al., 2017).

The four MAGs of species cluster 32 (*Methylobacter* sp. WM.3.3, DS2.3.46, and DS3.3.25 and *Methylomonas* sp. FW.017) do not fall within the *Methylobacter* or *Methylomonas* clades (Figure 2) but instead represent a different genus and species (92–99% dDDH and 98–100% ANI) (Supplementary Table S4). All these MAGs were derived from wastewater metagenomes (Zhang et al., 2017), and a species description cannot be formally proposed until a known isolate of this species is identified (Tindall et al., 2010). These strains are renamed instead as *Methylococcaceae* bacterium.

Surprisingly, both *Methylococcaceae* bacterium genomes, TMED282 (species cluster 26) and TMED69 (species cluster 27) (Figure 2), have high dDDH to other distant MAGs (Figure 4 and Supplementary Table S4). dDDH ranged from 78 to 79% when TMED282 was compared with genomes UBA659 (species cluster 14) and RSM_CP2 (species cluster 21), and from 78 to 88% when TMED69 was compared with genomes DS1.3.54, DS1.022 (species cluster 42), and UBA1114 (species cluster 23). In contrast, the same comparisons fell way below the 95% ANI same-species cut-off (61–63%). These inferred species clusters contradict the genome phylogeny (i.e., not monophyletic), and the difference in G+C content among strains within these clusters is 1–8% and 4–18%, respectively (Supplementary Table S5), suggesting different species (Rosselló-Móra and Amann, 2001; Meier-Kolthoff et al., 2014b).

### Resolving Species-Level Taxonomic Inconsistencies Between Close Relatives

*M. whittenburyi* ACM 3310^T^ and UCM-B-3033, *M. marinus* A45^T^, and *Methylobacter* sp. BBA5.1 exhibit a relatively high dDDH (88–100%) and ANI (98–100%) with each other and should therefore represent the same species (cluster 9) (Figure 2 and Supplementary Table S4). Previous genomic comparisons have also demonstrated that *M. whittenburyi* ACM 3310^T^ and *Methylobacter* sp. BBA5.1 have high sequence similarity (99% ANI) with *M. marinus* A45^T^ (Collins et al., 2017). With the two names appearing in the same publication, priority is determined by page number where the names appear (Rule 24b-2, section 5 of the *Bacteriological Code*; Lapage et al., 1992). Thus, name priority is granted to *M. marinus* over *M. whittenburyi* (Bowman et al., 1993). As such, *M. whittenburyi* (strain ACM 3310^T^) is proposed as a later heterotypic synonym of *M. marinus* (Note 3, Rule 24a, section 5 of the *Bacteriological Code*; Lapage et al., 1992). Also, UCM-B-3033 and BBA5.1 are identified as *Methylobacter marinus*. An emended description of *M. marinus* is included.

Comparisons between *M. agile* ATCC 35068^T^ and *M. album* BG8^T^ resulted in 90% dDDH and 99% ANI (Supplementary Table S4), and both strains should represent the same species (cluster 50) (Figure 2). This high ANI was also shown in a previous work (Kalyuzhnaya, 2016a). With *M. agile*, being the type species of the genus and described before *M. album* (Bowman et al., 1995), the former species should take name priority over *M. album* (Rule 23a, section 5 and Rule 38, section 7 of the *Bacteriological Code;* Lapage et al., 1992); thus, *M. album* is presumed to be a later heterotypic synonym. However, there is circumstantial evidence of misidentification of the currently deposited ATCC (American Type Culture Collection) strains for both species. Preliminary data suggests that strain ATCC 35068^T^ (*M. agile*) is phenotypically similar to *M. album*, whereas strain ATCC 33003^T^ (*M. album*) is similar to *Methylosinus sporium* (Dr. M. G. Kalyuzhnaya, personal communication). It is possible that the strains were interchanged, mislabeled, or lost, and that the *M. agile* ATCC 35068^T^ genome sequence was from an *M. album* isolate. Tracking the original *M. agile* and *M. album* isolates is difficult since they were originally isolated and identified in 1970 (as *Methylomonas agile* and *Methylomonas albus*, respectively) (Whittenbury et al., 1970). Also, it is unfortunately common for methanotrophic isolates to be lost from or mis-deposited into culture collections, as these isolates take substantial efforts to maintain (Bowman, 2016b). Both strains are no longer available from the NCIMB (National Collection of Industrial Food and Marine Bacteria), where the strains were also initially deposited (Bowman et al., 1993, 1995). This matter requires further investigation, with the need to track laboratories that would have maintained stocks of both strains to perform extensive phenotypic characterizations and to re-sequence the genomes to confirm their identities, as well as to replace or deposit the strains in multiple culture collections.

It was suggested that members of the newly proposed *Methylotuvimicrobium* gen. nov., *M. alcaliphilum* comb. nov. 20Z^T^ and “*M. buryatense*” comb. nov. 5G, be considered a single species based on 95% ANI (Kalyuzhnaya, 2016a). While we obtained a similar borderline ANI value, dDDH is only 63%, providing a much stronger argument to maintain the two as different species (clusters 47 and 48, respectively) (Figure 2 and Supplementary Table S4). They can be distinguished from each other since “*M. buryatense*” comb. nov., unlike *M. alcaliphilum* comb. nov., is able to grow at 45°C and contains a soluble methane monooxygenase (sMMO) (Table 1).

Genomic comparisons also revealed misclassifications of some *Methylomonas* strains. Type strains *M. methanica* NCIMB 11130^T^ and “*M. denitrificans*” FJG1^T^ exhibit 92% dDDH and 100% ANI with each other, indicating that they should be considered as the same species (cluster 38) (Figure 2 and Supplementary Table S4). Both strains are pale pink, utilize flagella for motility, and are neutrophilic and mesophilic, which are characteristics of *M. methanica* (Kits et al., 2015; Bowman, 2016b). With “*M. denitrificans*” being described much later than *M. methanica*, the former name is proposed to be a later heterotypic synonym of the latter. Moreover, strains R-45363, R-45371, and MC09 (species clusters 33, 34, and 44, respectively), currently identified as *M. methanica* (Boden et al., 2011; Heylen et al., 2016), do not belong to this species since they are not monophyletic with the *M. methanica* strains (Figure 2) and genomic comparisons revealed only 21–35% dDDH and 74–88% ANI (Supplementary Table S4). Additionally, sMMO is predicted to be absent in *M. methanica* (Heylen et al., 2016; Orata et al., 2018) but present in R-45363, R-45371, and MC09 (Boden et al., 2011; Heylen et al., 2016). *Methylomonas lenta* R-45370 (species cluster 43) (Hoefman et al., 2014; Heylen et al., 2016) is the closest known relative of strain MC09 (Figure 2), but they do not represent the same species (21% dDDH and 76% ANI) (Supplementary Table S4). Therefore, these three strains are renamed as *Methylomonas* sp. and each represent a putative novel species. They need to be extensively characterized phenotypically in comparison to their closest relatives to justify their classification into novel species.

### Incorporating Whole-Genome Sequencing to Prokaryotic Taxonomy: Advantages and Limitations

**FIGURE 5:**
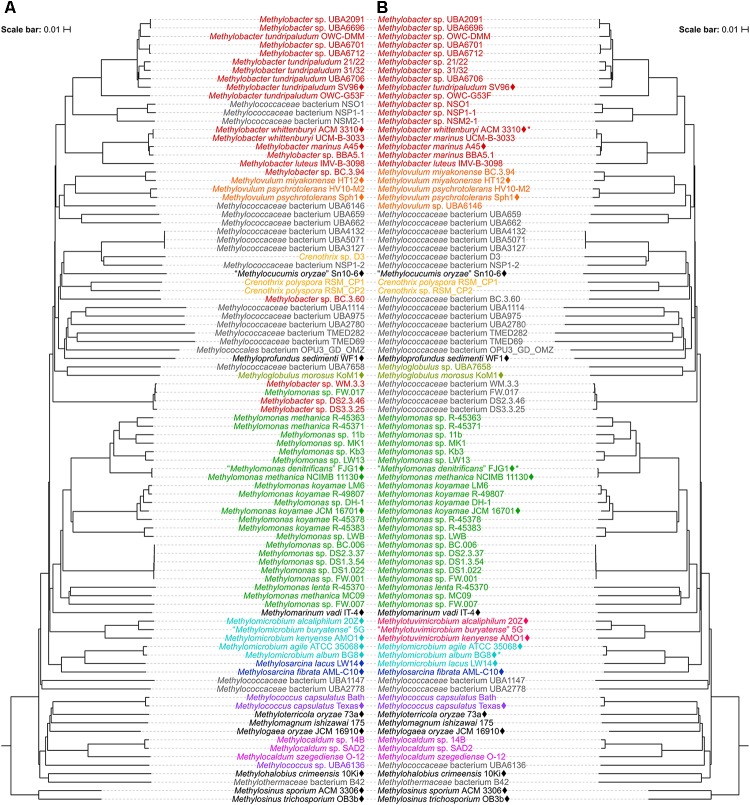
Reclassification of the *Methylococcales* genomes. The phylogenetic trees are the same trees as in Figure 2 (mirror image). Names and genera (colored when containing at least two members) are shown **(A)** before and **(B)** after reclassifications. **(B)** Asterisks indicate later heterotypic synonyms of their senior counterparts in the same species clusters as shown in Figure 2.

The results from reclassifications are summarized in Figure 5 and Supplementary Table S5. For the sake of consistency and accuracy of the analyses, the reclassifications in this study were mainly limited to strains with available genome sequences. The genomes of the majority of *Methylococcales* type strains have yet to be sequenced. In fact, only 22 out of the 49 currently identified type strains (including those with effectively but not validly published names) have sequenced genomes (Figure 2). Nonetheless, we considered exceptions for some type strains that are not genome-sequenced, for currently available information (i.e., highly supported clades in both 16S rRNA and PmoA phylogenies, experimental DDH, phenotypic similarities, etc.) are sufficient to support our proposed reclassifications. Incorporation of genome sequence data is not mandatory in officially describing a novel taxon (Tindall et al., 2010), however, the routine description of species on the basis of their genome sequences is recommended, which would allow for type strains to be uniquely and unambiguously identified to avoid redundancies and inconsistencies in taxonomy (Whitman, 2011; Thompson et al., 2015; Chun et al., 2018). Additionally, since the current taxonomic practice is highly reliant on comparisons with type strains (Tindall et al., 2010), this also becomes a limitation (Konstantinidis et al., 2017). Several genomes in our dataset were binned from metagenomes obtained from various methane sinks (Skennerton et al., 2015; Oswald et al., 2017; Parks et al., 2017; Zhang et al., 2017; Smith et al., 2018) that currently do not show any affinity with sequenced type strains and therefore cannot be officially designated species names.

It is still recommended that a genomic approach to descriptions of novel taxa must not abandon the fundamental principles of taxonomy, which includes the incorporation of phenotypic data (Tindall et al., 2010; Kämpfer and Glaeser, 2012; Garrity, 2016). Our reclassifications incorporated phenotypic characterizations when needed (e.g., between *Methylotuvimicrobium* gen. nov., *Methylomicrobium*, and *Methylosarcina*) (Table 1). However, phenotypic characterizations should be genome phylogeny-guided, not the reverse (Hahnke et al., 2016; Nouioui et al., 2018). Inconsistencies between single-gene phylogenies (e.g., 16S rRNA and PmoA) and instances of poly- and paraphyly within *Methylococcales* have generally been overlooked in the past in favor of phenotypic similarities (Bowman, 2016a, 2018). Regardless of phenotype, inclusion of an isolate to a known or novel taxon should first and foremost be supported by genome phylogeny (i.e., highly supported monophyletic clade) and supplemented with whole-genome comparisons (i.e., AAI, dDDH, and ANI) so that extensive poly- and paraphyly do not confuse taxonomy and subsequent interpretation.

## Conclusion

Our work successfully established a whole-genome-based phylogeny for all currently available *Methylococcales* genome sequences. In addition, pairwise genome comparisons were used to supplement the robust phylogeny to confidently reclassify several previously identified genomes, as well as provide identities to some unidentified genomes, including MAGs. This work serves as a foundation for the classification/reclassification of current and future isolates within *Methylococcales*. This approach will enable consistent and reliable classification of *Methyloccocales*, which becomes more and more important as the number of isolated and sequenced strains is rapidly growing.

## Taxonomic Reclassifications

### Description of *Methylotuvimicrobium* gen. nov.

Me.thy.lo.tu.vi.mi.cro’bi.um. N.L. n. *methylum* (from French *méthyle*, back-formation from French *méthylène*, coined from Gr. n. *methu*, wine and Gr. n. *hulê*, wood), the methyl radical; N.L. pref. *methylo-*, pertaining to the methyl radical; N.L. neut. adj. *tuvi*, pertaining to Tuva, Russia, the isolation source of the type species and strain; N.L. neut. n. *microbium* (from Gr. adj. *mikros*, small, and Gr. n. *bios*, life), microbe; N.L. neut. n. *Methylotuvimicrobium*, methane-utilizing microbe first isolated from Tuva, Russia.

Cells are Gram-negative and mainly rods but can also vary to ellipsoids, ovoids, and coccoids. 0.5–1.5 μm × 0.8–3.0 μm in size. Reproduce by binary fission. Motile mainly by a single polar flagellum but some may possess up to three peritrichous flagella. Stacks of intracytoplasmic membrane present. Do not form cysts. Colonies are white to slightly cream. Grow in a wide range of pH from 6 to 11, temperature from 4 to 45°C, and salinity from 0.03 to 1.5 M NaCl, with varying optimal conditions per species. Some species are resistant to desiccation and heat (80°C). Some species possess an sMMO. The most abundant fatty acids are C_16:1_, C_16:1_ ω7c, or C_16:1_ ω5t, with type depending on the species. DNA G+C content from 48 to 50.2 mol%. The type species for the genus is *Methylotuvimicrobium alcaliphilum*.

### Description of *Methylotuvimicrobium alcaliphilum*, comb. nov.

Basonym: *Methylomicrobium alcaliphilum* (Kalyuzhnaya et al., 2008). The description is the same as that of the genus and for *Methylomicrobium alcaliphilum* (Kalyuzhnaya et al., 2008) with the following amendment. DNA G+C content of type strain 20Z^T^ (= VKM B-2133^T^ = NCIMB 14124^T^ = DSM 19304^T^) is 48.7 mol% based on its whole-genome sequence.

### Description of “*Methylotuvimicrobium buryatense*,” comb. nov.

Basonym: “*Methylomicrobium buryatense*” (Kaluzhnaya et al., 2001). The description is the same as that of the genus and for “*Methylomicrobium buryatense*” (Kaluzhnaya et al., 2001) with the following amendment. DNA G+C content of strain 5G is 48.7 mol% based on its whole-genome sequence.

### Description of *Methylotuvimicrobium kenyense*, comb. nov.

Basonym: *Methylomicrobium kenyense* (Kalyuzhnaya et al., 2008). The description is the same as that of the genus and for *Methylomicrobium kenyense* (Kalyuzhnaya et al., 2008) with the following amendment. DNA G+C content of type strain AMO1^T^ (= NCCB 97157^T^ = VKM B-2464^T^ = NCIMB 13566^T^ = DSM 19305^T^) is 50.2 mol% based on its whole-genome sequence.

### Description of *Methylotuvimicrobium japanense*, comb. nov.

Basonym: *Methylomicrobium japanense* (Kalyuzhnaya et al., 2008). The description is the same as that of the genus and for *Methylomicrobium japanense* (Kalyuzhnaya et al., 2008). Despite the absence of a genome-sequenced representative strain, 16S rRNA and PmoA phylogenies, phenotypic characteristics, and experimental DDH provided strong evidence for the placement of this species in the genus *Methylotuvimicrobium* gen. nov.

### Description of *Methylotuvimicrobium pelagicum*, comb. nov.

Basonym: *Methylomicrobium pelagicum* (Sieburth et al., 1987; Bowman et al., 1995). The description is the same as that of the genus and for *Methylomicrobium pelagicum* (Bowman et al., 1993, 1995). Despite the absence of a genome-sequenced representative strain, 16S rRNA phylogeny, phenotypic characteristics, and experimental DDH provided strong evidence for the placement of this species in the genus *Methylotuvimicrobium* gen. nov.

### Description of *Methylomicrobium lacus*, comb. nov.

Basonym: *Methylosarcina lacus* (Kalyuzhnaya et al., 2005). The description is the same as for *Methylosarcina lacus* (Kalyuzhnaya et al., 2005) with the following amendment. DNA G+C content of type strain LW14^T^ (= ATCC BAA-1047^T^ = JCM 13284^T^) is 54.7 mol% based on its whole-genome sequence.

### Emended Description of *Methylomicrobium* (Bowman et al., 1995) emend. (Kalyuzhnaya et al., 2008)

The description is the same as given by Bowman et al. (1995) and Kalyuzhnaya et al. (2008) with the following amendments. Cells are short rods or coccobacilli. 0.5–1.0 μm × 1.0–2.5 μm in size. Cells may be motile by means of a single polar flagellum or nonmotile. Mostly mesophilic, with optimum temperature for growth at 25–30°C; range, 4–37°C. Optimum pH for growth from 5.5 to 7; range, pH 4–9. All species possess a pMMO but no sMMO. The most abundant fatty acids are C_16:1_ ω5t or C_16:1_ ω8c, with type depending on the species. DNA G+C content from 52 to 59.6 mol%.

### Emended Description of *Methylosarcina* (Wise et al., 2001) emend. (Kalyuzhnaya et al., 2005)

The description is the same as given by Wise et al. (2001) and excludes the amendments by Kalyuzhnaya et al. (2005) due to the proposed transference of *Methylosarcina lacus* (Kalyuzhnaya et al., 2005) to the genus *Methylomicrobium (*Bowman et al., 1995) emend. (Kalyuzhnaya et al., 2008).

### Emended Description of *Methylobacter marinus* (Bowman et al., 1993)

The description is the same as given by Bowman et al. (1993) with the following amendments. Cells are 0.8–1.5 μm × 1.5–3.0 μm. May or may not require NaCl for growth. Optimum temperature for growth, ∼30–35°C; range, 15–40°C. DNA G+C content from 48.7 to 53.7 mol%. DNA G+C content of type strain A45^T^ (= Lidstrom A4^T^ = ACM 4717^T^) is 52.7 mol% based on its whole-genome sequence.

## Author Contributions

FO, DS, and LS conceived and supervised the work. FO and JM-K carried out the phylogenetic and comparative genomic analyses. FO drafted the original manuscript. All authors reviewed and approved the manuscript.

## Conflict of Interest Statement

The authors declare that the research was conducted in the absence of any commercial or financial relationships that could be construed as a potential conflict of interest.

## References

[B1] Ad Hoc Committee of the Judicial Commission of the ICSB (1989). *Approved Lists of Bacterial Names (Amended).* Washington, DC: ASM Press. 20806452

[B2] Aliyu H. Lebre P. Blom J. Cowan D. De Maayer P. (2016). Phylogenomic re-assessment of the thermophilic genus *Geobacillus*. *Syst. Appl. Microbiol.* 39 527–533. 10.1016/j.syapm.2016.09.004 27726901

[B3] Auch A. F. von Jan M. Klenk H. P. Göker M. (2010). Digital DNA–DNA hybridization for microbial species delineation by means of genome-to-genome sequence comparison. *Stand. Genomic Sci.* 2 117–134. 10.4056/sigs.531120 21304684 PMC3035253

[B4] Benson D. A. Cavanaugh M. Clark K. Karsch-Mizrachi I. Ostell J. Pruitt K. D. (2018). GenBank. *Nucleic Acids Res.* 46 D41–D47. 10.1093/nar/gkx1094 29140468 PMC5753231

[B5] Boden R. Cunliffe M. Scanlan J. Moussard H. Kits K. D. Klotz M. G. (2011). Complete genome sequence of the aerobic marine methanotroph *Methylomonas methanica* MC09. *J. Bacteriol.* 193 7001–7002. 10.1128/JB.06267-11 22123758 PMC3232845

[B6] Bowman J. P. (2016a). “*Methylococcaceae*,” in *Bergey’s Manual of Systematics of Archaea and Bacteria*, eds Whitman W. B. Rainey F. Kämpfer P. Trujillo M. Chun J. De Vos P. (Hoboken, NJ: John Wiley & Sons, Inc).

[B7] Bowman J. P. (2016b). “Methylomonas,” in *Bergey’s Manual of Systematics of Archaea and Bacteria*, eds Whitman W. B. Rainey F. Kämpfer P. Trujillo M. Chun J. De Vos P. (Hoboken, NJ: John Wiley & Sons, Inc).

[B8] Bowman J. P. (2018). “*Methylococcales*,” in *Bergey’s Manual of Systematics of Archaea and Bacteria*, eds Whitman W. B. Rainey F. Kämpfer P. Trujillo M. Chun J. De Vos P. (Hoboken, NJ: John Wiley & Sons, Inc).

[B9] Bowman J. P. Sly L. I. Nichols P. D. Hayward A. C. (1993). Revised taxonomy of the methanotrophs: description of *Methylobacter* gen. nov., emendation of *Methylococcus*, validation of *Methylosinus* and *Methylocystis* species, and a proposal that the family *Methylococcaceae* includes only the group I methanotrophs. *Int. J. Syst. Evol. Microbiol.* 43 735–753.

[B10] Bowman J. P. Sly L. I. Stackebrandt E. (1995). The phylogenetic position of the family *Methylococcaceae*. *Int. J. Syst. Bacteriol.* 45 182–185. 10.1099/00207713-45-1-182 7857800

[B11] Buchfink B. Xie C. Huson D. H. (2015). Fast and sensitive protein alignment using DIAMOND. *Nat. Methods* 12 59–60. 10.1038/nmeth.3176 25402007

[B12] Camacho C. Coulouris G. Avagyan V. Ma N. Papadopoulos J. Bealer K. (2009). BLAST+: architecture and applications. *BMC Bioinformatics* 10:421. 10.1186/1471-2105-10-421 20003500 PMC2803857

[B13] Castresana J. (2000). Selection of conserved blocks from multiple alignments for their use in phylogenetic analysis. *Mol. Biol. Evol.* 17 540–552. 10.1093/oxfordjournals.molbev.a026334 10742046

[B14] Chun J. Oren A. Ventosa A. Christensen H. Arahal D. R. da Costa M. S. (2018). Proposed minimal standards for the use of genome data for the taxonomy of prokaryotes. *Int. J. Syst. Evol. Microbiol.* 68 461–466. 10.1099/ijsem.0.002516 29292687

[B15] Chun J. Rainey F. A. (2014). Integrating genomics into the taxonomy and systematics of the *Bacteria* and *Archaea*. *Int. J. Syst. Evol. Microbiol.* 64 316–324. 10.1099/ijs.0.054171-0 24505069

[B16] Cicerone R. J. Oremland R. S. (1988). Biogeochemical aspects of atmospheric methane. *Glob. Biogeochem. Cycles* 2 299–327. 10.1029/GB002i004p00299

[B17] Collins D. A. Akberdin I. R. Kalyuzhnaya M. G. (2017). “*Methylobacter*,” in *Bergey’s Manual of Systematics of Archaea and Bacteria*, eds Whitman W. B. Rainey F. Kämpfer P. Trujillo M. Chun J. De Vos P. (Hoboken, NJ: John Wiley & Sons, Inc).

[B18] Dyksterhouse S. E. Gray J. P. Herwig R. P. Lara J. C. Staley J. T. (1995). *Cycloclasticus pugetii* gen. nov., sp. nov., an aromatic hydrocarbon-degrading bacterium from marine sediments. *Int. J. Syst. Bacteriol.* 45 116–123. 10.1099/00207713-45-1-116 7857792

[B19] Edenhofer O. Pichs-Madruga R. Sokona Y. Kadner S. Minx J. C. Brunner S. (2014). “IPCC, 2014: technical summary,” in *Climate Change 2014: Mitigation of Climate Change. Contribution of Working Group III to the Fifth Assessment Report of the Intergovernmental Panel on Climate Change*, eds Edenhofer O. Pichs-Madruga R. Sokona Y. Farahani E. Kadner S. Seyboth K. (Cambridge: Cambridge University Press), 33–107.

[B20] Foster J. W. Davis R. H. (1966). A methane-dependent coccus, with notes on classification and nomenclature of obligate, methane-utilizing bacteria. *J. Bacteriol.* 91 1924–1931. 5937247 10.1128/jb.91.5.1924-1931.1966 PMC316146

[B21] Frindte K. Maarastawi S. A. Lipski A. Hamacher J. Knief C. (2017). Characterization of the first rice paddy cluster I isolate, *Methyloterricola oryzae* gen. nov., sp. nov. and amended description of *Methylomagnum ishizawai*. *Int. J. Syst. Evol. Microbiol.* 67 4507–4514. 10.1099/ijsem.0.002319 28984554

[B22] Garrity G. M. (2016). A new genomics-driven taxonomy of Bacteria and Archaea: are we there yet? *J. Clin. Microbiol.* 54 1956–1963. 10.1128/JCM.00200-16 27194687 PMC4963521

[B23] Geiselbrecht A. D. (2015). “*Cycloclasticus*,” in *Bergey’s Manual of Systematics of Archaea and Bacteria*, eds Whitman W. B. Rainey F. Kämpfer P. Trujillo M. Chun J. De Vos P. (Hoboken, NJ: John Wiley & Sons, Inc).

[B24] Goris J. Konstantinidis K. T. Klappenbach J. A. Coenye T. Vandamme P. Tiedje J. M. (2007). DNA–DNA hybridization values and their relationship to whole-genome sequence similarities. *Int. J. Syst. Evol. Microbiol.* 57 81–91. 10.1099/ijs.0.64483-0 17220447

[B25] Hahnke R. L. Meier-Kolthoff J. P. García-López M. Mukherjee S. Huntemann M. Ivanova N. N. (2016). Genome-based taxonomic classification of *Bacteroidetes*. *Front. Microbiol.* 7:2003 10.3389/fmicb.2016.02003 PMC5167729 28066339

[B26] Hanson R. S. Hanson T. E. (1996). Methanotrophic bacteria. *Microbiol. Rev.* 60 439–471. 8801441 10.1128/mr.60.2.439-471.1996 PMC239451

[B27] Henz S. R. Huson D. H. Auch A. F. Nieselt-Struwe K. Schuster S. C. (2005). Whole-genome prokaryotic phylogeny. *Bioinformatics* 21 2329–2335. 10.1093/bioinformatics/bth324 15166018

[B28] Heyer J. Berger U. Hardt M. Dunfield P. F. (2005). *Methylohalobius crimeensis* gen. nov., sp. nov., a moderately halophilic, methanotrophic bacterium isolated from hypersaline lakes of Crimea. *Int. J. Syst. Evol. Microbiol.* 55 1817–1826. 10.1099/ijs.0.63213-0 16166672

[B29] Heylen K. De Vos P. Vekeman B. (2016). Draft genome sequences of eight obligate methane oxidizers occupying distinct niches based on their nitrogen metabolism. *Genome Announc.* 4:e00421-16. 10.1128/genomeA.00421-16 27491982 PMC4974303

[B30] Hirayama H. (2016). “*Methylothermaceae*,” in *Bergey’s Manual of Systematics of Archaea and Bacteria*, eds Whitman W. B. Rainey F. Kämpfer P. Trujillo M. Chun J. De Vos P. (Hoboken, NJ: John Wiley & Sons, Inc).

[B31] Hirayama H. Abe M. Miyazaki M. Nunoura T. Furushima Y. Yamamoto H. (2014). *Methylomarinovum caldicuralii* gen. nov., sp. nov., a moderately thermophilic methanotroph isolated from a shallow submarine hydrothermal system, and proposal of the family *Methylothermaceae fam*. nov. *Int. J. Syst. Evol. Microbiol.* 64 989–999. 10.1099/ijs.0.058172-0 24425820

[B32] Hodcroft E. (2016). *TreeCollapserCL 4.* Available at: http://emmahodcroft.com/TreeCollapseCL.html [accessed September 27 2018].

[B33] Hoefman S. Heylen K. De Vos P. (2014). *Methylomonas lenta* sp. nov., a methanotroph isolated from manure and a denitrification tank. *Int. J. Syst. Evol. Microbiol.* 64 1210–1217. 10.1099/ijs.0.057794-0 24408530

[B34] Hyatt D. Chen G. L. LoCascio P. F. Land M. L. Larimer F. W. Hauser L. J. (2010). Prodigal: prokaryotic gene recognition and translation initiation site identification. *BMC Bioinformatics* 11:119. 10.1186/1471-2105-11-119 20211023 PMC2848648

[B35] Iguchi H. Yurimoto H. Sakai Y. (2011). *Methylovulum miyakonense* gen. nov., sp. nov., a Type I methanotroph isolated from forest soil. *Int. J. Syst. Evol. Microbiol.* 61 810–815. 10.1099/ijs.0.019604-0 20435749

[B36] Kaluzhnaya M. Khmelenina V. Eshinimaev B. Suzina N. Nikitin D. Solonin A. (2001). Taxonomic characterization of new alkaliphilic and alkalitolerant methanotrophs from soda lakes of the Southeastern Transbaikal region and description of *Methylomicrobium buryatense* sp. nov. *Syst. Appl. Microbiol.* 24 166–176. 10.1078/0723-2020-00028 11518319

[B37] Kalyuzhnaya M. G. (2016a). “*Methylomicrobium*,” in *Bergey’s Manual of Systematics of Archaea and Bacteria*, eds Whitman W. B. Rainey F. Kämpfer P. Trujillo M. Chun J. De Vos P. (Hoboken, NJ: John Wiley & Sons, Inc).

[B38] Kalyuzhnaya M. G. (2016b). “Methylosarcina,” in *Bergey’s Manual of Systematics of Archaea and Bacteria*, eds Whitman W. B. Rainey F. Kämpfer P. Trujillo M. Chun J. De Vos P. (Hoboken, NJ: John Wiley & Sons, Inc).

[B39] Kalyuzhnaya M. G. Khmelenina V. Eshinimaev B. Sorokin D. Fuse H. Lidstrom M. (2008). Classification of halo(alkali)philic and halo(alkali)tolerant methanotrophs provisionally assigned to the genera *Methylomicrobium* and *Methylobacter* and emended description of the genus *Methylomicrobium*. *Int. J. Syst. Evol. Microbiol.* 58 591–596. 10.1099/ijs.0.65317-0 18319461

[B40] Kalyuzhnaya M. G. Lamb A. E. McTaggart T. L. Oshkin I. Y. Shapiro N. Woyke T. (2015). Draft genome sequences of gammaproteobacterial methanotrophs isolated from Lake Washington sediment. *Genome Announc.* 3:e00103-15. 10.1128/genomeA.00103-15 25767239 PMC4357761

[B41] Kalyuzhnaya M. G. Stolyar S. M. Auman A. J. Lara J. C. Lidstrom M. E. Chistoserdova L. (2005). *Methylosarcina lacus* sp. nov., a methanotroph from Lake Washington, Seattle, USA, and emended description of the genus *Methylosarcina*. *Int. J. Syst. Evol. Microbiol.* 55 2345–2350. 10.1099/ijs.0.63405-0 16280494

[B42] Kalyuzhnaya M. G. Xing X. H. (2018). *Methane Biocatalysis: Paving the Way to Sustainability.* Cham: Springer.

[B43] Kämpfer P. Glaeser S. P. (2012). Prokaryotic taxonomy in the sequencing era – the polyphasic approach revisited. *Environ. Microbiol.* 14 291–317. 10.1111/j.1462-2920.2011.02615.x 22040009

[B44] Katoh K. Standley D. M. (2013). MAFFT multiple sequence alignment software version 7: improvements in performance and usability. *Mol. Biol. Evol.* 30 772–780. 10.1093/molbev/mst010 23329690 PMC3603318

[B45] Kearse M. Moir R. Wilson A. Stones-Havas S. Cheung M. Sturrock S. (2012). Geneious Basic: an integrated and extendable desktop software platform for the organization and analysis of sequence data. *Bioinformatics* 28 1647–1649. 10.1093/bioinformatics/bts199 22543367 PMC3371832

[B46] Kits K. D. Klotz M. G. Stein L. Y. (2015). Methane oxidation coupled to nitrate reduction under hypoxia by the gammaproteobacterium *Methylomonas denitrificans*, sp. nov. type strain FJG1. *Environ. Microbiol.* 17 3219–3232. 10.1111/1462-2920.12772 25580993

[B47] Knief C. (2015). Diversity and habitat preferences of cultivated and uncultivated aerobic methanotrophic bacteria evaluated based on *pmoA* as molecular marker. *Front. Microbiol.* 6:1346. 10.3389/fmicb.2015.01346 26696968 PMC4678205

[B48] Konstantinidis K. T. Rosselló-Móra R. Amann R. (2017). Uncultivated microbes in need of their own taxonomy. *ISME J.* 11 2399–2406. 10.1038/ismej.2017.113 28731467 PMC5649169

[B49] Konstantinidis K. T. Tiedje J. M. (2005). Towards a genome-based taxonomy for prokaryotes. *J. Bacteriol.* 187 6258–6264. 10.1128/JB.187.18.6258-6264.2005 16159757 PMC1236649

[B50] Kyrpides N. C. Hugenholtz P. Eisen J. A. Woyke T. Göker M. Parker C. T. (2014). Genomic encyclopedia of *Bacteria* and *Archaea*: sequencing a myriad of type strains. *PLoS Biol.* 12:e1001920. 10.1371/journal.pbio.1001920 25093819 PMC4122341

[B51] Lapage S. P. Sneath P. H. A. Lessel E. F. Skerman V. B. D. Seeliger H. P. R. Clark W. A. (1992). “Chapter 3: rules of nomenclature with recommendations,” in *International Code of Nomenclature of Bacteria: Bacteriological Code, 1990 Revision*, eds Lapage S. P. Sneath P. H. A. Lessel E. F. Skerman V. B. D. Seeliger H. P. R. Clark W. A. (Washington, DC: ASM Press). 21089234

[B52] Lefort V. Desper R. Gascuel O. (2015). FastME 2.0: a comprehensive, accurate, and fast distance-based phylogeny inference program. *Mol. Biol. Evol.* 32 2798–2800. 10.1093/molbev/msv150 26130081 PMC4576710

[B53] Letunic I. Bork P. (2016). Interactive tree of life (iTOL) v3: an online tool for the display and annotation of phylogenetic and other trees. *Nucleic Acids Res.* 44 W242–W245. 10.1093/nar/gkw290 27095192 PMC4987883

[B54] Li Y. Xue H. Sang S. Q. Lin C. L. Wang X. Z. (2017). Phylogenetic analysis of family *Neisseriaceae* based on genome sequences and description of *Populibacter corticis* gen. nov., sp. nov., a member of the family *Neisseriaceae*, isolated from symptomatic bark of *Populus* × *euramericana* canker. *PLoS One* 12:e0174506. 10.1371/journal.pone.0174506 28406911 PMC5390963

[B55] Lopes-Santos L. Castro D. B. A. Ferreira-Tonin M. Corrêa D. B. A. Weir B. S. Park D. (2017). Reassessment of the taxonomic position of *Burkholderia andropogonis* and description of *Robbsia andropogonis* gen. nov., comb. nov. *Antonie Van Leeuwenhoek* 110 727–736. 10.1007/s10482-017-0842-6 28190154

[B56] Luo C. Rodriguez-R L. M. Konstantinidis K. T. (2014). MyTaxa: an advanced taxonomic classifier for genomic and metagenomic sequences. *Nucleic Acids Res.* 42 e73. 10.1093/nar/gku169 24589583 PMC4005636

[B57] Martins da Costa E. Azarias Guimaraes A. Pereira Vicentin R. de Almeida Ribeiro P. R. Ribas Leao A. C. (2017). *Bradyrhizobium brasilense* sp. nov., a symbiotic nitrogen-fixing bacterium isolated from Brazilian tropical soils. *Arch. Microbiol.* 199 1211–1221. 10.1007/s00203-017-1390-1 28551732

[B58] Mateos-Rivera A. Islam T. Marshall I. P. G. Schreiber L. Øvreås L. (2018). High-quality draft genome of the methanotroph *Methylovulum psychrotolerans* str. HV10-M2 isolated from plant material at a high-altitude environment. *Stand. Genomic Sci.* 13:10. 10.1186/s40793-018-0314-2 29686747 PMC5898042

[B59] Médigue C. Calteau A. Cruveiller S. Gachet M. Gautreau G. Josso A. (2017). MicroScope—an integrated resource for community expertise of gene functions and comparative analysis of microbial genomic and metabolic data. *Brief. Bioinform.* 10.1093/bib/bbx113 [Epub ahead of print]. 28968784 PMC6931091

[B60] Meier-Kolthoff J. P. Auch A. F. Klenk H. P. Göker M. (2013). Genome sequence-based species delimitation with confidence intervals and improved distance functions. *BMC Bioinformatics* 14:60. 10.1186/1471-2105-14-60 23432962 PMC3665452

[B61] Meier-Kolthoff J. P. Auch A. F. Klenk H. P. Göker M. (2014a). Highly parallelized inference of large genome-based phylogenies. *Concurr. Comput. Pract. Exp.* 26 1715–1729. 10.1002/cpe.3112

[B62] Meier-Kolthoff J. P. Klenk H. P. Göker M. (2014b). Taxonomic use of DNA G+C content and DNA–DNA hybridization in the genomic age. *Int. J. Syst. Evol. Microbiol.* 64 352–356. 10.1099/ijs.0.056994-0 24505073

[B63] Mukherjee S. Seshadri R. Varghese N. J. Eloe-Fadrosh E. A. Meier-Kolthoff J. P. Göker M. (2017). 1,003 reference genomes of bacterial and archaeal isolates expand coverage of the tree of life. *Nat. Biotechnol.* 35 676–683. 10.1038/nbt.3886 28604660

[B64] Nouioui I. Carro L. Garcia-Lopez M. Meier-Kolthoff J. P. Woyke T. Kyrpides N. C. (2018). Genome-based taxonomic classification of the phylum *Actinobacteria*. *Front. Microbiol.* 9:2007 10.3389/fmicb.2018.02007 PMC6113628 30186281

[B65] Ogiso T. Ueno C. Dianou D. Huy T. V. Katayama A. Kimura M. (2012). *Methylomonas koyamae* sp. nov., a Type I methane-oxidizing bacterium from floodwater of a rice paddy field. *Int. J. Syst. Evol. Microbiol.* 62 1832–1837. 10.1099/ijs.0.035261-0 21984674

[B66] Orata F. D. Kits K. D. Stein L. Y. (2018). Complete genome sequence of *Methylomonas denitrificans* strain FJG1, an obligate aerobic methanotroph that can couple methane oxidation with denitrification. *Genome Announc.* 6:e00276-18. 10.1128/genomeA.00276-18 29700144 PMC5920175

[B67] Orata F. D. Xu Y. Gladney L. M. Rishishwar L. Case R. J. Boucher Y. (2016). Characterization of clinical and environmental isolates of *Vibrio cidicii* sp. nov., a close relative of *Vibrio navarrensis*. *Int. J. Syst. Evol. Microbiol.* 66 4148–4155. 10.1099/ijsem.0.001327 27468862

[B68] Oshkin I. Y. Belova S. E. Danilova O. V. Miroshnikov K. K. Rijpstra W. I. Sinninghe Damste J. S. (2016). *Methylovulum psychrotolerans* sp. nov., a cold-adapted methanotroph from low-temperature terrestrial environments, and emended description of the genus *Methylovulum*. *Int. J. Syst. Evol. Microbiol.* 66 2417–2423. 10.1099/ijsem.0.001046 27031985

[B69] Oswald K. Graf J. S. Littmann S. Tienken D. Brand A. Wehrli B. (2017). *Crenothrix* are major methane consumers in stratified lakes. *ISME J.* 11 2124–2140. 10.1038/ismej.2017.77 28585934 PMC5563964

[B70] Pandit P. S. Hoppert M. Rahalkar M. C. (2018). Description of ’Candidatus *Methylocucumis oryzae*’, a novel Type I methanotroph with large cells and pale pink colour, isolated from an Indian rice field. *Antonie Van Leeuwenhoek* 111 2473–2484. 10.1007/s10482-018-1136-3 30066210

[B71] Parks D. H. (2017). *CompareM: a Toolbox for Comparative Genomics.* Available at: https://github.com/dparks1134/CompareM [accessed July 3 2018].

[B72] Parks D. H. Chuvochina M. Waite D. W. Rinke C. Skarshewski A. Chaumeil P. A. (2018). A standardized bacterial taxonomy based on genome phylogeny substantially revises the tree of life. *Nat. Biotechnol.* 36 996–1004. 10.1038/nbt.4229 30148503

[B73] Parks D. H. Rinke C. Chuvochina M. Chaumeil P. A. Woodcroft B. J. Evans P. N. (2017). Recovery of nearly 8,000 metagenome-assembled genomes substantially expands the tree of life. *Nat. Microbiol.* 2 1533–1542. 10.1038/s41564-017-0012-7 28894102

[B74] Parte A. C. (2014). LPSN—list of prokaryotic names with standing in nomenclature. *Nucleic Acids Res.* 42 D613–D616. 10.1093/nar/gkt1111 24243842 PMC3965054

[B75] Qin Q. L. Xie B. B. Zhang X. Y. Chen X. L. Zhou B. C. Zhou J. (2014). A proposed genus boundary for the prokaryotes based on genomic insights. *J. Bacteriol.* 196 2210–2215. 10.1128/JB.01688-14 24706738 PMC4054180

[B76] Rahalkar M. C. Pandit P. (2018). Genome-based insights into a putative novel Methylomonas species (strain Kb3), isolated from an Indian rice field. *Gene Rep.* 13 9–13. 10.1016/j.genrep.2018.08.004

[B77] Rambaut A. (2007). *FigTree.* Available at: http://tree.bio.ed.ac.uk/software/figtree [accessed September 27 2018].

[B78] Ramette A. Tiedje J. M. (2007). Biogeography: an emerging cornerstone for understanding prokaryotic diversity, ecology, and evolution. *Microb. Ecol.* 53 197–207. 10.1007/s00248-005-5010-2 17106806

[B79] Richter M. Rosselló-Móra R. (2009). Shifting the genomic gold standard for the prokaryotic species definition. *Proc. Natl. Acad. Sci. U.S.A.* 106 19126–19131. 10.1073/pnas.0906412106 19855009 PMC2776425

[B80] Rosselló-Móra R. Amann R. (2001). The species concept for prokaryotes. *FEMS Microbiol. Rev.* 25 39–67. 10.1111/j.1574-6976.2001.tb00571.x 11152940

[B81] Sieburth J. M. Johnson P. W. Eberhardt M. A. Sieracki M. E. Lidstrom M. Laux D. (1987). The first methane-oxidizing bacterium from the upper mixing layer of the deep ocean: Methylomonas pelagica sp. nov. *Curr. Microbiol.* 14 285–293. 10.1007/Bf01568138

[B82] Skennerton C. T. Ward L. M. Michel A. Metcalfe K. Valiente C. Mullin S. (2015). Genomic reconstruction of an uncultured hydrothermal vent gammaproteobacterial methanotroph (family *Methylothermaceae*) indicates multiple adaptations to oxygen limitation. *Front. Microbiol.* 6:1425. 10.3389/fmicb.2015.01425 26779119 PMC4688376

[B83] Smith G. J. Angle J. C. Solden L. M. Borton M. A. Morin T. H. Daly R. A. (2018). Members of the genus *Methylobacter* are inferred to account for the majority of aerobic methane oxidation in oxic soils from a freshwater wetland. *mBio* 9:e00815-18. 10.1128/mBio.00815-18 30401770 PMC6222125

[B84] Stamatakis A. (2014). RAxML version 8: a tool for phylogenetic analysis and post-analysis of large phylogenies. *Bioinformatics* 30 1312–1313. 10.1093/bioinformatics/btu033 24451623 PMC3998144

[B85] Stoecker K. Bendinger B. Schöning B. Nielsen P. H. Nielsen J. L. Baranyi C. (2006). Cohn’s Crenothrix is a filamentous methane oxidizer with an unusual methane monooxygenase. *Proc. Natl. Acad. Sci. U.S.A.* 103 2363–2367. 10.1073/pnas.0506361103 16452171 PMC1413686

[B86] Strong P. J. Xie S. Clarke W. P. (2015). Methane as a resource: can the methanotrophs add value? *Environ. Sci. Technol.* 49 4001–4018. 10.1021/es504242n 25723373

[B87] Tatusova T. Ciufo S. Fedorov B. O’Neill K. Tolstoy I. (2014). RefSeq microbial genomes database: new representation and annotation strategy. *Nucleic Acids Res.* 42 D553–D559. 10.1093/nar/gkt1274 24316578 PMC3965038

[B88] Thompson C. C. Amaral G. R. Campeao M. Edwards R. A. Polz M. F. Dutilh B. E. (2015). Microbial taxonomy in the post-genomic era: rebuilding from scratch? *Arch. Microbiol.* 197 359–370. 10.1007/s00203-014-1071-2 25533848

[B89] Tindall B. J. Rosselló-Móra R. Busse H. J. Ludwig W. Kämpfer P. (2010). Notes on the characterization of prokaryote strains for taxonomic purposes. *Int. J. Syst. Evol. Microbiol.* 60 249–266. 10.1099/ijs.0.016949-0 19700448

[B90] Vigliotta G. Nutricati E. Carata E. Tredici S. M. De Stefano M. Pontieri P. (2007). *Clonothrix fusca* Roze 1896, a filamentous, sheathed, methanotrophic γ-proteobacterium. *Appl. Environ. Microbiol.* 73 3556–3565. 10.1128/AEM.02678-06 17416684 PMC1932688

[B91] Völker H. Schweisfurth R. Hirsch P. (1977). Morphology and ultrastructure of *Crenothrix polyspora* Cohn. *J. Bacteriol.* 131 306–313. 873887 10.1128/jb.131.1.306-313.1977 PMC235423

[B92] Wartiainen I. Hestnes A. G. McDonald I. R. Svenning M. M. (2006). *Methylobacter tundripaludum* sp. nov., a methane-oxidizing bacterium from Arctic wetland soil on the Svalbard islands, Norway (78° N). *Int. J. Syst. Evol. Microbiol.* 56 109–113. 10.1099/ijs.0.63728-0 16403874

[B93] Whitman W. B. (2011). Intent of the nomenclatural Code and recommendations about naming new species based on genomic sequences. *Bull. BISMiS* 2 135–139.

[B94] Whitman W. B. Woyke T. Klenk H. P. Zhou Y. Lilburn T. G. Beck B. J. (2015). Genomic Encyclopedia of Bacterial and Archaeal Type Strains, Phase III: the genomes of soil and plant-associated and newly described type strains. *Stand. Genomic Sci.* 10:26. 10.1186/s40793-015-0017-x 26203337 PMC4511459

[B95] Whittenbury R. Phillips K. C. Wilkinson J. F. (1970). Enrichment, isolation and some properties of methane-utilizing bacteria. *J. Gen. Microbiol.* 61 205–218. 10.1099/00221287-61-2-205 5476891

[B96] Wirth J. S. Whitman W. B. (2018). Phylogenomic analyses of a clade within the roseobacter group suggest taxonomic reassignments of species of the genera *Aestuariivita*, *Citreicella*, *Loktanella*, *Nautella*, *Pelagibaca*, *Ruegeria*, *Thalassobius*, *Thiobacimonas* and *Tropicibacter*, and the proposal of six novel genera. *Int. J. Syst. Evol. Microbiol.* 68 2393–2411. 10.1099/ijsem.0.002833 29809121

[B97] Wise M. G. McArthur J. V. Shimkets L. J. (2001). *Methylosarcina fibrata* gen. nov., sp. nov. and *Methylosarcina quisquiliarum* sp. nov., novel Type 1 methanotrophs. *Int. J. Syst. Evol. Microbiol.* 51 611–621. 10.1099/00207713-51-2-611 11321107

[B98] Wu D. Hugenholtz P. Mavromatis K. Pukall R. Dalin E. Ivanova N. N. (2009). A phylogeny-driven genomic encyclopaedia of Bacteria and Archaea. *Nature* 462 1056–1060. 10.1038/nature08656 20033048 PMC3073058

[B99] Zhang Y. Kitajima M. Whittle A. J. Liu W. T. (2017). Benefits of genomic insights and CRISPR-Cas signatures to monitor potential pathogens across drinking water production and distribution systems. *Front. Microbiol.* 8:2036. 10.3389/fmicb.2017.02036 29097994 PMC5654357

