# Supplementary material for: Phylogenomic Analysis of the Gammaproteobacterial Methanotrophs (Order Methylococcales) Calls for the Reclassification of Members at the Genus and Species Levels
Source: Front Microbiol. 2018 Dec 19;9:3162. doi: 10.3389/fmicb.2018.03162 (PMC6315193; doi:10.3389/fmicb.2018.03162)
Supplement: Supplementary file 1 [file Data_Sheet_1.PDF]

frontiers

Supplementary Material

Phylogenomic Analysis of the Gammaproteobacterial Methanotrophs (Order *Methylococcales*) Calls for the Reclassification of Members at the Genus and Species Levels

Fabini D. Orata*, Jan P. Meier-Kolthoff, Dominic Sauvageau†, and Lisa Y. Stein†

***Correspondence:** Fabini D. Orata, orata@ualberta.ca
†These authors contributed equally to this work.

1. Supplementary Tables

Supplementary Table S1. 16S rRNA and PmoA sequences used in this study.

Species and strain	Accession numbers	
	**16S rRNA**	**PmoA**
"*Clonothrix fusca*" AW-b*	DQ984190.1	ABL64049.1
Crenothrix polyspora clone 4*	DQ295895.1	–
Methylobacter luteus ACM 3304^T	AF304195.1	–
Methylobacter luteus IMV-B-3098*	–	ATYJ01
Methylobacter marinus A45^T	AF304197.1	ARVS01
Methylobacter psychrophilus Z-0021^T	AF152597.1	AAX48776.1
Methylobacter tundripaludum SV96^T	AJ414655.1	AEGW02
Methylobacter whittenburyi ACM 3310^T	X72773.1	NA
Methylocaldum gracile VKM-14L^T	U89298.1	AAC04380.1
Methylocaldum marinum S8^T	AB894129.1	BAO51827.1
Methylocaldum szegediense OR2^T	U89300.1	AAC04381.1
Methylocaldum tepidum LK6^T	U89297.1	AAC04382.1
Methylococcus capsulatus Texas^T	AJ563935.1	AUKJ01
Methylococcus mobilis LMD 77.28^T	HQ676599.1	–
Methylococcus thermophilus IMV-2Yu^T	X73819.1	–
"*Methylocucumis oryzae*" Sn10-6^T	KP793700.1	LAJX01
Methylogaea oryzae JCM 16910^T	EU672873.1	BBDL01
Methyloglobulus morosus KoM1^T	JN386974.1	AYLO01
Methylohalobius crimeensis 10Ki^T	AJ581837.1	BAM71040.1
Methylomagnum ishizawai RS11D-Pr^T	AB669155.1	BAK78887.1
Methylomarinovum caldicuralii IT-9^T	AB301718.2	BAF62078.2
Methylomarinum vadi IT-4^T	AB301717.2	JPON01
Methylomicrobium agile ATCC 35068^T	X72767.1	JPOJ01
Methylomicrobium album BG8^T	X72777.1	CM001475.1
Methylomicrobium alcaliphilum 20Z^T	EF495157.1	FO082060.1
"*Methylomicrobium buryatense*" 5B^T	AF307138.1	AAL09399.1
Methylomicrobium japanense NI^T	D89279.1	BAE86885.1
Methylomicrobium kenyense AMO1^T	AJ132384.1	NA
Methylomicrobium pelagicum AA-23^T	X72775.1	–

Species and strain	Accession numbers	
	**16S rRNA**	**PmoA**
Methylomonas aurantiaca JB103^T	X72776.1	–
"*Methylomonas denitrificans*" FJG1^T	CP014476.1	CP014476.1
Methylomonas fodinarum LD2^T	X72778.1	–
Methylomonas koyamae JCM 16701^T	AB538964.1	BBCK01
Methylomonas lenta R-45377^T	FR798962.1	CCW45942.1
Methylomonas methanica NCIMB 11130^T	AF304196.1	LUUF01
Methylomonas paludis MG30^T	HE801216.1	CCH22593.1
"*Methylomonas rubra*" 15sh^T	AF304194.1	–
Methylomonas scandinavica SR5^T	AJ131369.1	–
Methyloparacoccus murrellii R-49797^T	HF558990.1	CCW45946.1
Methyloprofundus sedimenti WF1^T	KF484906.1	LPUF01
Methylosarcina fibrata AML-C10^T	AF177296.1	ARCU01
Methylosarcina lacus LW14^T	AY007296.1	AZUN01
Methylosarcina quisquiliarum AML-D4^T	AF177297.1	AAF04266.1
Methylosoma difficile LC 2^T	DQ119050.1	ABD13901.1
Methylosphaera hansonii AM6^T	U67929.1	–
Methyloterricola oryzae 73a^T	JYNS01	JYNS01
"*Methylothermus subterraneus*" HTM55^T	AB536747.2	BAI52935.1
"*Methylothermus thermalis*" MYHT^T	AY829009.1	AAX37294.1
Methylovulum miyakonense HT12^T	AB501287.1	AQZU01
Methylovulum psychrotolerans Sph1^T	KT381578.1	PGFZ01
Methylosinus sporium ACM 3306^T	Y18946.1	PWB93641.1
Methylosinus trichosporium OB3b^T	Y18947.1	ATQ66669.1

Sequence versions are indicated in the NCBI accession numbers. The PmoA sequences of M. whittenburyi *ACM 3310^T and* M. kenyense *AMO1^T were obtained from the MicroScope database and therefore have no NCBI accession numbers. Sequences that were not available from type strains were taken from different representative strains (indicated by asterisks). NA, not applicable; –, no sequence available.*

Supplementary Table S2. Whole-genome sequences used in this study.

Genome	Accession number
Crenothrix polyspora RSM_CP1	FUKI01
Crenothrix polyspora RSM_CP2	FUKJ01
Crenothrix sp. D3	MBQZ01
Methylobacter luteus IMV-B-3098	ATYJ01
Methylobacter marinus A45^T	ARVS01
Methylobacter sp. BBA5.1	JQKS01
Methylobacter sp. BC.3.60	PESA01
Methylobacter sp. BC.3.94	PERX01
Methylobacter sp. DS2.3.46	PERP01
Methylobacter sp. DS3.3.25	PERK01
Methylobacter sp. UBA2091	DCZF01
Methylobacter sp. UBA6696	DKJI01
Methylobacter sp. UBA6701	DKJD01
Methylobacter sp. UBA6712	DKIS01
Methylobacter sp. WM.3.3	PERF01
Methylobacter tundripaludum 21/22	JMLA01
Methylobacter tundripaludum 31/32	JPOH01
Methylobacter tundripaludum OWC-DMM	PTIZ01
Methylobacter tundripaludum OWC-G53F	PTIY01
Methylobacter tundripaludum SV96^T	AEGW02
Methylobacter tundripaludum UBA6706	DKIY01
Methylobacter whittenburyi ACM 3310^T	NA
Methylobacter whittenburyi UCM-B-3033	JQNS01
Methylocaldum sp. 14B	MSCV01
Methylocaldum sp. SAD2	MUGL01
Methylocaldum szegediense O-12	ATXX01
Methylococcaceae bacterium NSM2-1	NQJI01
Methylococcaceae bacterium NSO1	NQJJ01
Methylococcaceae bacterium NSP1-1	NQJG01
Methylococcaceae bacterium NSP1-2	NQJH01
Methylococcaceae bacterium TMED282	NHMB01
Methylococcaceae bacterium TMED69	NHDW01
Methylococcaceae bacterium UBA1114	DCBF01
Methylococcaceae bacterium UBA1147	DBZZ01
Methylococcaceae bacterium UBA2778	DEII01
Methylococcaceae bacterium UBA2780	DEIG01
Methylococcaceae bacterium UBA3127	DEZR01
Methylococcaceae bacterium UBA4132	DFWO01
Methylococcaceae bacterium UBA5071	DIAT01
Methylococcaceae bacterium UBA6146	DIUY01
Methylococcaceae bacterium UBA659	DBNZ01
Methylococcaceae bacterium UBA662	DBNW01
Methylococcaceae bacterium UBA7658	DLHW01
Methylococcaceae bacterium UBA975	DBBV01
Methylococcales bacterium OPU3_GD_OMZ	MPSY01

**Genome**	**Accession number**
Methylococcus capsulatus Bath	AE017282.2
Methylococcus capsulatus Texas^T	AUKJ01
Methylococcus sp. UBA6136	DIVI01
"*Methylocucumis oryzae*" Sn10-6^T	LAJX01
Methylogaea oryzae JCM 16910^T	BBDL01
Methyloglobulus morosus KoM1^T	AYLO01
Methylohalobius crimeensis 10Ki^T	ATXB01
Methylomagnum ishizawai 175	FXAM01
Methylomarinum vadi IT-4^T	JPON01
Methylomicrobium agile ATCC 35068^T	JPOJ01
Methylomicrobium album BG8^T	CM001475.1
Methylomicrobium alcaliphilum 20Z^T	FO082060.1
"*Methylomicrobium buryatense*" 5G	AOTL01
Methylomicrobium kenyense AMO1^T	NA
"*Methylomonas denitrificans*" FJG1^T	CP014476.1
Methylomonas koyamae JCM 16701^T	BBCK01
Methylomonas koyamae LM6	CP023669.1
Methylomonas koyamae R-45378	LUUJ01
Methylomonas koyamae R-45383	LUUK01
Methylomonas koyamae R-49807	LUUL01
Methylomonas lenta R-45370	LUUI01
Methylomonas methanica MC09	CP002738.1
Methylomonas methanica NCIMB 11130^T	LUUF01
Methylomonas methanica R-45363	LUUG01
Methylomonas methanica R-45371	LUUH01
Methylomonas sp. 11b	AZXK01
Methylomonas sp. BC.006	PESC01
Methylomonas sp. DH-1	CP014360.1
Methylomonas sp. DS1.022	PERS01
Methylomonas sp. DS1.3.54	PERR01
Methylomonas sp. DS2.3.37	PERQ01
Methylomonas sp. FW.001	PERW01
Methylomonas sp. FW.007	PERU01
Methylomonas sp. FW.017	PERV01
Methylomonas sp. Kb3	PIZT01
Methylomonas sp. LW13	JNLB01
Methylomonas sp. LWB	MKMC01
Methylomonas sp. MK1	AQOV01
Methyloprofundus sedimenti WF1^T	LPUF01
Methylosarcina fibrata AML-C10^T	ARCU01
Methylosarcina lacus LW14^T	AZUN01
Methyloterricola oryzae 73a^T	JYNS01
Methylothermaceae bacterium B42	LSNW01
Methylovulum miyakonense HT12^T	AQZU01
Methylovulum psychrotolerans HV10-M2	CP022129.1
Methylovulum psychrotolerans Sph1^T	PGFZ01
Methylosinus sporium ACM 3306^T	PUIV01

Genome	Accession number
Methylosinus trichosporium OB3b^T	CP023737.1

Sequence versions are indicated in the NCBI accession numbers. The genome sequences of M. whittenburyi *ACM 3310^T and* M. kenyense *AMO1^T were obtained from the MicroScope database and therefore have no NCBI accession numbers. NA, not applicable.*

Supplementary Table S3. AAI and POCP from pairwise whole-genome comparisons.

Percentage of conserved proteins (POCP) (upper right); **Average amino acid identity (AAI)** (lower left)

Previous name	Name after reclassification	*Methylobacter* sp. UBA2091	*Methylobacter* sp. UBA6696	*Methylobacter tundripaludum* OWC-DMM	*Methylobacter* sp. UBA6701	*Methylobacter* sp. UBA6712	*Methylobacter tundripaludum* 21/22	*Methylobacter tundripaludum* 31/32	*Methylobacter tundripaludum* UBA6706	*Methylobacter tundripaludum* SV96^{T}	*Methylobacter tundripaludum* OWC-G53F	*Methylococcaceae* bacterium NSO1	*Methylococcaceae* bacterium NSP1-1	*Methylococcaceae* bacterium NSM2-1	*Methylobacter whittenburyi* ACM 3310^{T}	*Methylobacter whittenburyi* UCM-B-3033	*Methylobacter marinus* A45^{T}	*Methylobacter* sp. BBA5.1	*Methylobacter luteus* IMV-B-3098	*Methylobacter* sp. BC.3.94	*Methylovulum miyakonense* HT12^{T}	*Methylovulum psychrotolerans* HV10-M2	*Methylovulum psychrotolerans* Sph1^{T}	*Methylococcaceae* bacterium UBA6146	*Methylococcaceae* bacterium UBA659	*Methylococcaceae* bacterium UBA662	*Methylococcaceae* bacterium UBA4132	*Methylococcaceae* bacterium UBA5071	*Methylococcaceae* bacterium UBA3127	*Crenothrix* sp. D3	*Methylococcaceae* bacterium NSP1-2	"*Methylocucumis oryzae*" Sn10-6^{T}	*Crenothrix polyspora* RSM_CP1	*Crenothrix polyspora* RSM_CP2	*Methylobacter* sp. BC.3.60	*Methylococcaceae* bacterium UBA1114	*Methylococcaceae* bacterium UBA975	*Methylococcaceae* bacterium UBA2780	*Methylococcaceae* bacterium TMED282	*Methylococcaceae* bacterium TMED69	*Methylococcales* bacterium OPU3_GD_OMZ	*Methyloprofundus sedimenti* WF1^{T}	*Methylococcaceae* bacterium UBA7658	*Methyloglobulus morosus* KoM1^{T}	*Methylobacter* sp. WM.3.3	*Methylomonas* sp. FW.017	*Methylobacter* sp. DS2.3.46	*Methylobacter* sp. DS3.3.25	*Methylomonas methanica* R-45363	*Methylomonas methanica* R-45371	*Methylomonas* sp. 11b	*Methylomonas* sp. MK1	*Methylomonas* sp. Kb3	*Methylomonas* sp. LW13	"*Methylomonas denitrificans*" FJG1^{T}	*Methylomonas methanica* NCIMB 11130^{T}	*Methylomonas koyamae* LM6	*Methylomonas koyamae* R-49807	*Methylomonas* sp. DH-1	*Methylomonas koyamae* JCM 16701^{T}	*Methylomonas koyamae* R-45378	*Methylomonas koyamae* R-45383	*Methylomonas* sp. LWB	*Methylomonas* sp. BC.006	*Methylomonas* sp. DS2.3.37	*Methylomonas* sp. DS1.3.54	*Methylomonas* sp. DS1.022	*Methylomonas* sp. FW.001	*Methylomonas lenta* R-45370	*Methylomonas methanica* MC09	*Methylomonas* sp. FW.007	*Methylomarinum vadi* IT-4^{T}	*Methylomicrobium alcaliphilum* 20Z^{T}	"*Methylomicrobium buryatense*" 5G	*Methylomicrobium kenyense* AMO1^{T}	*Methylomicrobium agile* ATCC 35068^{T}	*Methylomicrobium album* BG8^{T}	*Methylosarcina lacus* LW14^{T}	*Methylosarcina fibrata* AML-C10^{T}	*Methylococcaceae* bacterium UBA1147	*Methylococcaceae* bacterium UBA2778	*Methylococcus capsulatus* Bath	*Methylococcus capsulatus* Texas^{T}	*Methyloterricola oryzae* 73a^{T}	*Methylomagnum ishizawai* 175	*Methylogaea oryzae* JCM 16910^{T}	*Methylocaldum* sp. 14B	*Methylocaldum* sp. SAD2	*Methylocaldum szegediense* O-12	*Methylococcus* sp. UBA6136	*Methylohalobius crimeensis* 10Ki^{T}	*Methylothermaceae* bacterium B42
Methylobacter sp. UBA2091	*Methylobacter* sp. UBA2091		89	77	65	75	75	71	65	72	65	29	36	40	49	49	51	51	50	48	48	44	44	52	41	46	49	49	49	48	42	30	44	41	29	33	35	39	16	21	38	44	45	50	42	41	43	46	48	48	47	48	48	48	48	49	48	47	48	29	47	47	47	49	48	47	44	46	49	47	50	48	47	47	47	51	51	52	48	33	35	41	42	41	37	21	38	37	38	31	39	41
Methylobacter sp. UBA6696	*Methylobacter* sp. UBA6696	100		76	65	73	74	70	65	71	64	29	37	40	49	49	51	51	50	48	48	44	44	52	42	47	49	49	49	48	42	30	44	42	30	34	35	39	15	20	38	44	45	50	41	41	43	46	48	47	47	48	48	48	48	49	48	47	47	29	47	46	47	50	49	47	45	47	49	47	50	47	47	47	47	50	50	52	48	33	35	41	41	40	37	21	38	37	38	31	39	40
Methylobacter tundripaludum OWC-DMM	*Methylobacter* sp. OWC-DMM	97	97		66	77	79	75	67	77	66	29	36	40	51	51	53	52	52	49	49	46	46	52	40	45	49	49	50	48	42	31	45	42	29	33	35	39	15	21	38	46	46	52	43	41	44	46	49	50	49	50	49	50	50	51	49	48	48	30	49	48	49	48	48	46	44	46	50	49	50	49	48	48	48	52	53	54	50	34	36	42	43	42	38	22	40	39	39	31	40	41
Methylobacter sp. UBA6701	*Methylobacter* sp. UBA6701	96	97	97		79	64	61	59	62	57	26	32	35	44	43	45	45	44	42	42	38	39	44	36	40	42	42	42	41	36	27	39	37	26	28	30	33	13	17	33	39	40	44	37	36	38	41	41	41	41	42	42	42	42	42	42	41	42	27	41	40	41	42	42	40	38	40	42	41	43	41	41	41	42	44	44	46	43	28	29	35	36	36	32	19	34	33	33	27	34	35
Methylobacter sp. UBA6712	*Methylobacter* sp. UBA6712	97	97	97	100		74	71	65	71	66	29	35	39	50	50	52	52	51	48	49	45	45	52	40	46	49	49	49	48	41	31	45	42	29	33	35	38	15	20	38	45	46	51	42	41	43	45	48	48	47	49	48	49	49	49	48	48	48	30	48	47	48	48	48	46	44	46	48	47	50	47	47	47	48	50	51	52	49	33	35	41	42	41	37	21	39	38	38	31	40	41
Methylobacter tundripaludum 21/22	*Methylobacter* sp. 21/22	96	96	97	96	96		87	66	75	65	28	35	39	51	50	53	53	53	49	49	45	46	51	40	45	49	49	49	47	42	30	44	42	28	33	35	38	15	21	38	45	46	51	42	40	43	45	50	50	49	51	50	50	51	51	50	49	50	30	49	48	48	48	47	46	43	45	50	48	50	48	47	47	48	51	52	53	50	33	35	42	42	42	37	22	40	38	39	31	39	40
Methylobacter tundripaludum 31/32	*Methylobacter* sp. 31/32	96	96	97	96	96	98		63	71	63	27	33	37	49	49	51	51	51	47	47	44	45	49	37	43	46	46	46	45	40	29	42	40	27	31	33	36	14	19	35	44	43	48	40	38	41	42	48	48	49	49	48	49	49	50	49	48	48	29	47	46	46	45	44	43	41	42	48	47	48	46	45	45	46	49	50	51	48	31	34	40	40	40	36	21	38	37	38	29	38	38
Methylobacter tundripaludum UBA6706	*Methylobacter* sp. UBA6706	96	96	97	96	96	97	96		63	59	27	34	37	45	45	47	47	46	44	43	40	40	47	38	42	45	45	44	43	39	28	40	38	28	30	32	35	14	19	35	40	41	44	38	36	39	42	42	43	42	43	43	43	43	44	43	43	42	27	42	41	42	44	44	43	40	42	44	42	45	43	42	42	42	45	46	47	44	30	31	37	38	37	33	20	35	34	34	29	35	37
Methylobacter tundripaludum SV96^{T}	*Methylobacter tundripaludum* SV96^{T}	96	97	97	96	97	97	96	97		65	27	34	38	49	49	52	51	51	48	48	45	44	51	38	43	47	47	47	46	40	30	43	41	27	32	34	37	15	20	36	44	44	50	41	39	42	43	48	48	48	49	48	49	49	49	49	48	47	29	47	46	47	46	45	44	41	43	49	47	50	49	47	47	47	50	51	53	48	32	34	41	41	40	37	21	39	37	38	30	38	39
Methylobacter tundripaludum OWC-G53F	*Methylobacter* sp. OWC-G53F	87	87	87	86	87	87	87	87	87		30	37	41	51	51	53	52	50	50	50	47	47	53	42	48	51	51	51	50	43	32	46	43	30	35	37	40	16	21	40	48	48	51	44	43	45	47	50	50	49	50	50	51	50	51	51	49	50	30	50	49	50	50	50	48	45	48	50	50	52	49	49	49	50	51	52	52	51	35	36	43	44	42	39	22	39	38	39	33	41	42
Methylococcaceae bacterium NSO1	*Methylobacter* sp. NSO1	80	79	79	79	80	80	80	79	79	79		39	34	25	24	26	26	26	27	26	25	24	33	30	33	32	31	32	30	30	17	27	27	24	24	24	26	12	15	29	26	29	27	27	25	27	26	23	23	23	24	24	24	23	24	25	24	24	16	24	22	23	31	31	30	27	30	26	24	27	28	26	26	28	27	28	28	26	25	21	25	25	22	19	13	19	18	20	20	23	26
Methylococcaceae bacterium NSP1-1	*Methylobacter* sp. NSP1-1	80	79	80	79	80	80	79	79	79	79	97		42	31	30	33	32	32	33	33	31	30	40	37	41	40	39	39	36	37	21	34	32	27	29	30	32	15	19	35	32	35	35	32	30	33	34	30	30	30	31	30	30	30	31	31	30	31	19	30	29	30	39	39	38	35	38	32	30	35	34	32	32	34	34	35	36	33	31	27	32	32	28	25	15	25	24	26	27	29	32
Methylococcaceae bacterium NSM2-1	*Methylobacter* sp. NSM2-1	79	79	79	78	79	79	79	79	79	79	88	89		34	34	36	35	35	36	35	33	32	43	39	42	42	42	42	40	38	22	35	34	30	30	31	33	15	19	36	35	37	37	34	34	35	37	31	31	31	32	31	31	32	33	32	32	32	20	31	30	31	40	40	39	36	39	35	32	36	36	35	34	36	37	37	39	36	30	29	35	36	30	26	16	27	26	28	28	31	34
Methylobacter whittenburyi ACM 3310^{T}	*Methylobacter whittenburyi* ACM 3310^{T}*	75	75	75	74	75	75	74	74	75	75	76	76	76		99	82	81	64	44	44	41	41	46	36	41	42	42	42	42	36	28	41	37	24	31	32	37	14	19	35	45	43	46	39	37	41	43	46	46	44	46	45	45	45	45	45	45	45	28	45	43	44	44	43	41	39	41	48	47	48	51	51	51	50	49	50	49	50	30	34	39	39	39	37	19	41	40	40	29	42	40
Methylobacter whittenburyi UCM-B-3033	*Methylobacter marinus* UCM-B-3033	75	75	75	74	75	74	74	74	75	75	76	76	76	100		82	81	64	44	44	41	41	46	36	41	42	42	42	42	36	28	41	37	24	31	32	37	14	19	34	45	43	46	39	37	41	43	46	45	44	46	45	45	45	45	46	45	45	28	45	43	44	44	43	41	39	41	48	47	48	52	51	51	50	49	50	50	50	30	34	39	39	39	37	19	41	40	40	29	42	40
Methylobacter marinus A45^{T}	*Methylobacter marinus* A45^{T}	75	75	75	74	75	75	75	74	75	75	76	76	76	99	99		84	66	46	46	43	42	49	39	44	45	45	45	44	38	29	42	38	26	32	34	39	15	20	36	47	44	48	40	40	43	45	46	46	46	47	47	47	47	47	47	46	46	29	47	45	46	46	46	44	42	44	49	48	49	53	52	52	51	51	52	52	51	32	36	41	42	41	38	20	43	42	42	30	43	42
Methylobacter sp. BBA5.1	*Methylobacter marinus* BBA5.1	75	75	75	74	75	74	74	75	74	75	76	76	76	98	98	99		68	47	46	42	43	48	38	43	44	44	44	44	38	29	42	38	26	32	33	38	15	19	36	47	44	47	41	39	43	45	47	47	46	47	47	47	47	47	48	47	47	29	47	45	45	45	45	43	41	43	50	48	49	52	52	52	51	51	52	51	53	32	35	40	41	41	37	19	43	42	41	30	43	42
Methylobacter luteus IMV-B-3098	*Methylobacter luteus* IMV-B-3098	76	76	76	75	76	75	75	75	75	76	76	76	76	86	86	86	86		47	46	42	43	48	38	43	45	45	45	44	38	28	42	38	25	30	32	36	14	19	35	46	45	48	39	38	41	42	45	45	45	46	46	46	46	46	46	45	45	28	45	44	45	43	43	41	40	41	47	47	47	47	49	48	48	50	51	53	51	32	35	40	41	40	37	19	41	40	40	30	40	39
Methylobacter sp. BC.3.94	*Methylovulum miyakonense* BC.3.94	73	73	73	72	73	73	73	73	73	73	75	75	74	71	71	71	71	71		76	57	57	56	44	50	46	46	46	46	40	33	45	41	25	30	31	35	15	19	35	41	45	48	40	38	41	43	46	46	46	47	47	47	47	47	47	46	46	28	46	45	47	44	44	42	40	42	45	46	47	42	43	43	43	47	47	48	46	31	33	40	40	38	37	19	37	36	36	30	36	38
Methylovulum miyakonense HT12^{T}	*Methylovulum miyakonense* HT12^{T}	73	73	73	72	73	73	72	72	73	73	75	75	74	71	71	71	71	71	97		58	58	55	43	49	46	46	46	47	39	33	45	42	25	30	31	35	15	19	34	42	45	48	40	38	41	43	46	46	46	48	47	47	47	48	47	47	47	29	46	46	48	44	44	42	40	42	45	47	47	42	43	44	43	47	47	48	45	31	33	40	40	38	38	19	37	36	36	30	37	38
Methylovulum psychrotolerans HV10-M2	*Methylovulum psychrotolerans* HV10-M2	72	72	72	71	72	72	72	71	72	72	73	74	73	70	70	70	70	70	79	79		76	53	41	47	44	44	44	44	37	32	44	39	23	29	30	33	14	18	33	39	42	44	38	36	39	41	41	41	41	43	42	42	42	42	43	42	42	26	42	41	42	42	42	40	38	40	43	42	44	39	40	41	41	43	44	45	42	30	31	38	38	36	37	18	34	33	33	29	34	35
Methylovulum psychrotolerans Sph1^{T}	*Methylovulum psychrotolerans* Sph1^{T}	72	71	72	71	71	71	71	71	72	71	73	74	73	70	70	70	70	70	79	79	98		53	40	45	43	43	43	44	36	32	43	39	23	28	29	32	13	18	32	39	42	44	37	35	38	40	42	42	42	43	42	42	42	43	43	42	42	26	42	41	42	41	41	39	37	39	43	42	44	39	40	41	40	43	44	44	42	29	30	37	38	36	37	18	34	33	33	28	34	35
Methylococcaceae bacterium UBA6146	*Methylovulum* sp. UBA6146	75	75	75	74	75	75	75	75	75	75	77	77	76	73	73	73	73	73	75	75	74	73		54	63	53	53	53	52	46	34	49	46	31	36	38	42	18	23	42	47	50	49	44	43	46	47	46	45	45	46	46	46	46	47	47	46	46	28	45	44	45	53	52	50	47	51	50	48	52	48	49	49	49	49	50	51	47	38	38	44	45	41	38	20	37	36	38	35	41	44
Methylococcaceae bacterium UBA659	*Methylococcaceae* bacterium UBA659	69	69	69	68	69	70	69	69	70	69	70	71	70	69	69	69	69	69	70	70	70	70	72		65	46	46	46	43	40	27	42	40	29	33	34	38	17	21	40	37	43	40	39	36	40	41	34	34	34	35	35	35	35	35	37	36	36	22	36	34	35	50	50	49	45	49	39	37	41	39	39	38	40	41	41	42	39	35	32	39	39	33	32	16	30	29	31	33	35	39
Methylococcaceae bacterium UBA662	*Methylococcaceae* bacterium UBA662	68	68	69	68	68	68	68	68	68	69	69	70	69	68	68	68	68	68	69	69	69	69	71	78		51	51	51	49	45	31	48	43	33	38	39	43	19	24	44	42	49	46	44	42	45	47	40	40	40	41	41	41	40	41	43	42	43	25	42	40	41	58	58	56	51	56	45	43	48	44	45	44	46	47	47	48	45	40	36	44	45	38	36	18	35	34	36	36	40	44
Methylococcaceae bacterium UBA4132	*Methylococcaceae* bacterium UBA4132	73	73	73	73	73	73	73	73	74	73	75	75	75	71	71	71	71	71	71	71	71	71	73	68	67		92	92	60	54	28	48	43	31	35	37	40	18	22	42	43	47	47	42	39	42	44	41	41	41	42	41	41	42	43	43	42	43	26	42	40	42	49	49	47	44	47	44	42	46	44	43	42	44	47	47	50	47	37	36	41	42	39	34	19	35	34	36	33	38	41
Methylococcaceae bacterium UBA5071	*Methylococcaceae* bacterium UBA5071	74	74	73	73	73	74	74	73	74	73	76	75	75	71	71	71	71	71	72	71	71	71	73	68	67	100		93	61	54	28	48	44	31	35	37	40	18	22	42	43	48	47	41	39	42	44	41	41	41	42	42	42	42	43	43	42	43	26	42	41	42	49	49	47	44	47	45	42	46	44	43	42	44	47	47	50	47	37	36	42	42	39	34	20	35	34	35	32	38	41
Methylococcaceae bacterium UBA3127	*Methylococcaceae* bacterium UBA3127	73	73	73	73	73	73	73	73	74	73	75	75	75	71	71	71	71	71	72	71	71	70	73	68	67	100	100		60	55	28	48	44	31	35	37	40	18	22	42	43	47	47	42	40	42	44	41	41	41	42	42	42	42	43	43	43	43	26	42	41	42	49	49	47	45	48	44	43	46	44	43	42	44	47	47	50	47	37	36	41	42	39	34	19	35	34	35	33	38	41
Crenothrix sp. D3	*Methylococcaceae* bacterium D3	74	74	74	73	74	74	74	73	74	73	75	75	75	71	71	71	71	71	71	71	71	70	73	68	67	84	84	84		50	29	46	43	28	34	35	38	16	21	39	42	46	45	39	37	40	42	42	42	41	43	42	42	42	43	43	42	43	26	42	40	42	47	46	45	42	45	45	43	46	43	42	43	44	45	45	47	43	35	34	41	42	37	35	19	33	33	34	31	36	38
Methylococcaceae bacterium NSP1-2	*Methylococcaceae* bacterium NSP1-2	74	74	74	73	74	74	74	73	74	74	76	76	75	72	72	72	72	72	72	71	71	71	73	68	67	84	84	84	85		25	40	38	28	31	31	34	16	19	36	36	41	40	36	34	36	38	34	34	34	35	34	34	35	36	36	34	35	23	34	33	34	41	41	40	37	40	37	35	39	37	37	36	37	39	40	42	38	32	30	35	35	31	28	18	28	28	29	27	32	35
"*Methylocucumis oryzae*" Sn10-6^{T}	"*Methylocucumis oryzae*" Sn10-6^{T}	68	68	68	68	68	68	68	67	68	68	70	70	69	67	67	67	67	68	70	70	69	69	70	68	67	68	68	68	68	67		28	27	16	20	20	22	9	12	23	26	30	30	26	25	27	27	29	28	28	29	29	29	28	29	30	29	29	21	29	29	29	30	29	29	28	28	29	29	30	28	28	28	28	29	29	29	29	20	21	25	25	25	24	15	23	22	22	19	24	24
Crenothrix polyspora RSM_CP1	*Crenothrix polyspora* RSM_CP1	72	72	72	71	72	72	72	71	72	72	73	73	73	69	69	70	69	70	69	69	69	69	71	67	66	70	70	70	71	71	66		57	27	32	34	37	16	21	38	40	48	49	39	37	40	41	41	40	40	41	41	41	40	41	41	41	41	25	41	39	40	46	46	44	41	45	43	41	45	42	44	43	44	45	46	46	44	33	34	38	39	36	35	17	34	33	34	31	36	39
Crenothrix polyspora RSM_CP2	*Crenothrix* sp. RSM_CP2	73	73	73	72	73	73	73	72	73	73	74	74	74	71	71	71	71	71	70	71	70	69	72	67	67	71	71	71	71	71	67	82		25	30	32	35	15	19	36	36	45	45	36	34	37	37	37	37	36	37	37	37	37	37	38	37	37	24	37	36	37	42	41	40	37	40	39	38	42	39	39	40	40	41	42	43	40	31	31	34	35	32	31	17	31	30	31	28	34	37
Methylobacter sp. BC.3.60	*Methylococcaceae bacterium* BC.3.60	74	74	74	74	74	74	74	74	74	74	75	75	74	72	72	72	72	73	70	71	70	70	73	69	68	72	71	72	72	72	67	70	70		25	25	28	12	16	27	24	28	28	27	26	26	29	24	24	23	25	25	24	24	25	25	25	25	14	25	23	24	33	33	32	29	32	27	24	28	25	26	26	28	27	27	28	26	24	21	25	26	23	20	11	20	19	20	21	23	26
Methylococcaceae bacterium UBA1114	*Methylococcaceae* bacterium UBA1114	57	56	57	56	57	57	57	56	57	57	57	57	57	57	57	57	57	57	56	56	56	56	57	56	56	57	57	57	57	56	56	57	56	56		74	56	25	30	43	34	33	33	31	31	32	33	29	29	29	30	30	30	30	30	31	30	31	19	30	29	30	40	40	39	35	39	32	31	34	35	34	33	34	32	33	32	31	38	32	35	36	31	28	16	29	28	30	28	35	40
Methylococcaceae bacterium UBA975	*Methylococcaceae* bacterium UBA975	57	57	57	57	57	57	57	57	57	57	58	57	57	57	57	57	57	58	57	57	57	57	58	56	56	57	57	57	57	57	56	57	57	56	74		58	26	31	44	36	35	34	32	32	33	34	31	31	31	32	32	32	32	32	33	32	32	20	32	31	31	42	41	40	36	40	34	32	37	36	36	35	37	34	34	34	33	39	33	36	37	32	29	16	30	29	31	29	37	41
Methylococcaceae bacterium UBA2780	*Methylococcaceae* bacterium UBA2780	60	60	60	59	60	60	60	59	60	60	60	60	60	60	60	60	60	60	59	59	59	59	60	59	58	59	60	60	60	59	58	59	59	59	60	60		24	31	47	39	38	38	36	36	37	38	35	35	35	36	36	36	36	37	38	36	37	22	36	34	36	47	47	44	41	46	39	37	42	43	40	40	41	38	38	37	37	42	37	40	40	37	34	19	34	34	36	31	42	47
Methylococcaceae bacterium TMED282	*Methylococcaceae* bacterium TMED282	52	51	52	51	52	52	52	51	52	52	52	50	51	52	52	51	52	52	52	52	52	52	52	51	51	52	52	52	52	52	52	52	52	50	51	51	52		37	22	17	17	16	15	15	15	16	13	13	13	13	13	13	14	14	14	14	14	8	14	13	14	19	19	18	16	20	15	14	16	16	16	16	16	16	16	16	15	22	16	18	19	15	14	8	14	14	15	15	18	21
Methylococcaceae bacterium TMED69	*Methylococcaceae* bacterium TMED69	49	49	49	48	49	49	49	48	49	49	50	49	48	49	49	49	49	49	49	50	49	49	50	49	49	49	50	50	50	50	49	49	50	48	49	49	49	54		27	21	21	22	19	19	20	20	18	18	18	19	19	19	19	19	19	19	19	11	18	18	19	25	24	24	22	25	20	19	21	21	21	20	21	21	21	20	20	26	21	24	24	21	18	10	19	18	20	19	23	26
Methylococcales bacterium OPU3_GD_OMZ	*Methylococcaceae* bacterium OPU3_GD_OMZ	63	62	63	62	63	63	63	62	63	63	63	63	62	63	63	63	63	63	62	62	62	62	63	61	61	62	62	62	62	62	61	62	62	62	56	57	59	51	49		39	40	39	35	35	36	37	33	33	33	34	34	34	34	35	35	35	35	22	34	32	34	46	46	44	40	45	36	35	40	39	39	37	39	38	38	38	36	45	36	38	39	33	29	17	30	29	32	31	38	44
Methyloprofundus sedimenti WF1^{T}	*Methyloprofundus sedimenti* WF1^{T}	65	65	65	64	65	65	65	64	65	65	65	65	65	65	65	65	65	65	64	64	64	64	65	62	62	64	64	64	64	64	63	63	64	64	57	57	59	51	49	62		41	42	36	35	38	39	42	42	41	42	42	42	42	42	43	42	42	27	42	40	41	43	43	41	39	41	46	45	45	50	46	46	47	42	42	43	42	34	38	40	41	38	34	18	36	34	35	30	40	42
Methylococcaceae bacterium UBA7658	*Methyloglobulus* sp. UBA7658	70	70	70	69	70	70	70	70	70	70	71	71	71	68	68	69	68	69	68	68	67	67	69	66	66	69	69	69	68	68	65	69	69	68	57	57	59	52	50	61	63		56	41	39	42	42	42	42	41	43	43	43	42	43	43	42	42	26	42	40	42	47	47	45	42	45	44	43	45	43	44	43	44	47	48	48	47	35	36	40	41	40	36	18	36	35	36	32	38	42
Methyloglobulus morosus KoM1^{T}	*Methyloglobulus morosus* KoM1^{T}	71	71	71	70	71	71	71	70	71	71	73	73	72	69	69	69	69	69	69	69	68	68	71	67	67	70	70	70	70	70	66	70	70	69	57	57	59	52	49	62	63	76		44	42	46	47	47	46	46	48	47	47	47	47	48	47	47	28	46	45	46	47	47	45	43	45	47	47	47	46	46	46	46	50	51	51	50	34	35	42	42	44	39	19	40	39	39	31	39	41
Methylobacter sp. WM.3.3	*Methylococcaceae* bacterium WM.3.3	68	69	69	68	69	69	69	68	69	69	70	69	69	69	69	69	69	69	68	67	67	67	69	66	66	67	68	68	67	68	66	66	67	68	56	57	59	52	50	61	63	66	66		60	62	63	38	38	38	40	40	39	39	40	40	39	39	24	39	38	40	46	46	44	42	45	40	40	42	39	40	39	39	40	41	41	41	32	31	36	37	35	32	17	32	31	32	28	34	37
Methylomonas sp. FW.017	*Methylococcaceae* bacterium FW.017	68	68	68	68	68	68	68	68	68	69	69	70	68	69	69	68	68	69	68	68	67	67	69	66	66	67	67	67	67	67	66	66	67	67	56	57	59	52	50	61	63	66	67	99		63	57	37	37	38	39	39	39	38	39	40	39	40	23	39	37	38	45	45	43	41	44	39	40	41	38	38	37	38	38	39	39	38	32	30	36	37	34	32	16	32	31	32	28	34	37
Methylobacter sp. DS2.3.46	*Methylococcaceae* bacterium DS2.3.46	69	69	69	68	69	69	69	68	69	69	69	70	69	68	69	69	69	69	68	68	67	67	69	67	66	67	67	67	67	68	66	66	67	68	57	57	59	52	50	61	63	66	67	99	99		69	42	43	41	43	42	43	42	43	43	42	43	26	42	41	43	46	46	44	42	45	43	43	45	41	41	41	42	41	41	42	41	32	31	38	39	37	34	18	34	33	34	29	35	38
Methylobacter sp. DS3.3.25	*Methylococcaceae* bacterium DS3.3.25	68	68	68	67	68	68	68	67	68	68	68	69	68	68	68	68	68	68	67	67	67	67	68	66	65	66	66	66	66	66	65	66	66	67	55	56	58	51	48	60	62	65	67	99	98	99		42	42	42	43	43	44	43	44	44	43	43	25	43	42	44	50	50	48	45	49	43	44	47	43	42	43	43	43	44	43	42	32	31	39	40	39	36	18	36	35	36	30	37	40
Methylomonas methanica R-45363	*Methylomonas* sp. R-45363	67	67	67	67	67	67	67	67	67	68	68	68	67	67	67	67	66	67	66	66	66	66	67	65	64	66	66	66	65	66	64	65	65	67	56	57	59	52	49	61	63	64	64	65	65	65	64		81	74	77	74	74	72	73	66	66	65	40	66	62	62	51	50	48	46	48	59	58	61	48	48	49	47	46	47	47	45	29	32	38	38	43	37	19	39	38	38	29	36	37
Methylomonas methanica R-45371	*Methylomonas* sp. R-45371	67	67	67	67	67	67	67	67	67	68	68	68	67	67	67	67	67	67	67	66	66	66	67	65	64	66	66	66	65	66	64	65	65	67	56	57	59	52	49	61	63	64	64	66	65	66	64	97		75	76	73	74	73	74	64	64	64	40	64	61	62	50	49	47	45	47	58	57	60	48	47	48	46	46	46	45	46	29	32	38	38	42	37	20	39	38	37	28	35	38
Methylomonas sp. 11b	*Methylomonas* sp. 11b	67	67	67	67	67	67	67	67	67	68	68	68	67	67	67	67	66	67	67	67	66	66	67	65	64	66	66	66	65	66	64	65	65	67	56	57	59	52	49	61	63	64	65	65	65	65	64	93	93		78	74	76	71	73	65	65	64	40	65	61	62	50	49	47	46	47	56	57	59	47	46	47	45	45	46	46	45	29	31	38	38	41	37	20	38	37	37	28	35	37
Methylomonas sp. MK1	*Methylomonas* sp. MK1	67	67	68	67	67	67	67	67	67	68	68	68	67	67	67	66	66	67	67	66	66	66	67	65	64	66	66	66	65	66	64	65	65	67	56	57	59	52	49	61	63	64	64	65	65	65	64	93	93	96		77	76	73	74	67	67	67	41	66	63	64	52	51	49	47	49	57	59	61	48	48	48	46	47	47	46	46	30	32	39	39	42	38	20	40	39	38	29	36	38
Methylomonas sp. Kb3	*Methylomonas* sp. Kb3	67	67	68	67	67	67	67	67	67	68	67	68	68	67	67	67	66	67	66	66	66	66	67	65	64	66	66	66	66	66	64	65	65	67	56	57	59	51	49	61	63	64	64	65	65	65	64	91	91	92	92		89	71	73	67	67	66	41	67	63	64	52	52	49	47	49	56	59	60	48	47	47	47	46	47	46	45	29	32	39	39	42	38	20	39	38	38	29	36	38
Methylomonas sp. LW13	*Methylomonas* sp. LW13	67	67	68	67	67	67	67	67	67	68	68	68	67	67	67	67	67	67	66	66	66	65	67	65	64	66	66	66	66	66	64	65	65	67	56	57	59	51	49	61	63	64	64	65	65	65	65	91	92	93	92	98		73	74	68	68	67	42	68	64	65	52	52	50	47	49	56	59	60	48	47	47	47	47	47	46	46	29	32	39	39	42	38	20	39	38	38	29	36	38
"*Methylomonas denitrificans*" FJG1^{T}	"*Methylomonas denitrificans*" FJG1^{T}*	67	67	68	67	67	67	67	67	67	68	68	68	68	67	67	67	67	68	66	67	66	65	67	65	64	66	66	66	65	66	64	65	65	67	56	57	59	51	49	61	63	64	65	65	66	65	65	92	92	91	91	90	90		98	65	64	64	40	65	62	61	50	50	48	46	48	56	58	60	48	47	47	47	46	47	47	45	30	32	38	39	42	37	20	39	38	38	28	36	38
Methylomonas methanica NCIMB 11130^{T}	*Methylomonas methanica* NCIMB 11130^{T}	67	67	68	67	67	67	67	67	68	68	68	68	68	67	67	67	67	68	66	67	66	65	67	65	64	66	66	66	65	66	64	65	65	67	56	57	59	51	49	61	63	64	65	65	65	65	65	92	92	91	91	90	90	100		66	65	65	41	65	63	62	52	51	49	47	49	57	59	61	49	47	48	47	47	47	47	46	30	33	39	39	43	38	20	39	38	38	29	37	39
Methylomonas koyamae LM6	*Methylomonas koyamae* LM6	66	66	67	66	66	66	66	66	66	67	66	67	67	66	66	66	66	66	66	66	65	65	67	65	64	65	65	65	65	65	64	64	65	66	56	56	59	52	49	61	63	64	64	65	65	65	64	78	78	78	78	79	78	78	78		89	85	50	81	63	62	53	53	51	49	51	56	58	62	49	48	48	48	49	49	48	47	30	32	40	40	43	39	20	40	39	39	30	38	39
Methylomonas koyamae R-49807	*Methylomonas koyamae* R-49807	66	66	67	66	66	66	66	66	66	67	67	67	67	66	66	66	66	66	66	66	65	65	67	65	64	65	65	65	65	65	64	64	65	66	56	56	59	52	49	61	63	64	64	65	65	65	64	79	79	79	79	79	79	79	79	99		85	50	81	62	62	52	52	49	47	49	55	57	60	48	47	47	47	48	49	48	46	30	32	39	40	42	38	20	39	39	39	29	37	38
Methylomonas sp. DH-1	*Methylomonas koyamae* DH-1	66	66	67	66	66	66	66	66	66	67	67	67	67	66	66	66	66	67	66	66	65	65	67	65	64	65	65	65	65	65	64	65	65	66	56	57	59	52	49	61	63	64	64	65	65	65	64	78	78	78	78	79	79	79	79	98	98		49	80	62	62	53	52	50	48	50	55	58	60	48	47	47	47	48	49	48	46	30	33	40	40	43	39	20	40	39	39	29	37	39
Methylomonas koyamae JCM 16701^{T}	*Methylomonas koyamae* JCM 16701^{T}	66	66	66	65	66	66	66	66	66	67	67	67	67	66	66	66	66	67	66	66	66	65	67	65	64	65	65	65	65	65	64	65	65	65	56	57	59	51	49	61	63	64	64	65	65	64	64	79	79	79	79	79	79	79	79	97	97	97		49	38	38	31	31	30	29	30	34	35	36	30	29	30	29	29	30	29	28	18	20	23	24	26	23	15	24	24	23	18	22	23
Methylomonas koyamae R-45378	*Methylomonas* sp. R-45378	66	66	67	66	67	66	66	66	67	67	67	67	67	66	66	66	66	67	66	66	65	65	67	65	64	65	65	65	65	65	64	64	65	66	56	57	59	52	49	61	63	64	64	65	65	65	64	79	79	79	79	79	79	79	79	96	96	97	96		62	62	53	52	50	48	50	54	57	60	48	47	48	47	48	49	47	46	30	32	39	39	42	38	20	40	39	39	29	37	38
Methylomonas koyamae R-45383	*Methylomonas* sp. R-45383	66	66	66	65	66	66	66	65	66	66	67	67	66	66	66	66	66	67	65	65	65	65	66	65	64	65	65	65	65	65	64	64	64	65	56	56	58	51	49	61	63	64	64	64	65	64	64	76	76	76	76	76	76	76	76	76	76	76	77	76		80	50	49	47	45	47	52	55	56	46	44	45	44	45	46	44	44	29	31	38	38	40	38	19	38	37	36	28	35	36
Methylomonas sp. LWB	*Methylomonas* sp. LWB	66	66	66	65	66	66	66	65	66	66	66	67	66	66	66	66	66	66	65	65	65	65	66	64	64	65	65	65	65	65	63	64	64	66	56	57	59	52	49	61	63	64	64	64	64	64	64	76	76	76	76	76	76	76	76	76	77	76	77	76	97		51	51	49	47	49	53	57	58	46	45	46	45	46	46	44	45	30	32	39	39	42	38	20	39	38	37	29	36	38
Methylomonas sp. BC.006	*Methylomonas* sp. BC.006	65	65	65	64	65	65	65	65	65	66	65	65	65	65	65	65	66	66	65	65	65	65	65	64	64	64	65	65	64	64	63	64	64	65	56	57	58	51	49	60	63	63	64	64	64	64	63	72	72	72	72	72	72	72	72	72	72	72	72	72	72	72		98	92	84	91	54	52	60	51	49	48	50	48	49	47	45	40	37	45	46	42	39	20	39	37	39	37	43	47
Methylomonas sp. DS2.3.37	*Methylomonas* sp. DS2.3.37	65	65	65	64	65	65	65	65	65	66	65	65	65	65	65	65	66	66	65	65	65	65	65	64	64	64	65	65	64	64	63	64	64	65	56	57	58	51	49	60	63	63	64	64	64	64	63	72	72	72	72	72	72	72	72	72	72	72	72	72	72	72	100		92	85	91	53	52	60	50	49	48	50	48	48	47	45	39	37	45	46	42	39	20	38	37	39	37	42	47
Methylomonas sp. DS1.3.54	*Methylomonas* sp. DS1.3.54	65	65	65	64	65	65	65	65	65	65	65	65	65	65	65	65	65	66	65	65	65	65	65	64	64	64	65	65	64	64	63	64	64	65	56	56	58	51	49	60	62	63	64	64	63	64	63	72	72	72	72	72	72	72	72	72	72	72	72	72	72	71	100	100		91	86	51	50	58	49	47	46	48	46	47	45	43	38	36	43	44	40	37	19	37	35	37	36	41	45
Methylomonas sp. DS1.022	*Methylomonas* sp. DS1.022	65	65	65	64	65	65	65	65	65	65	65	65	65	65	65	65	66	66	65	65	65	65	65	64	64	64	64	64	64	64	63	64	64	65	56	57	58	51	48	60	63	63	64	66	66	65	65	72	72	72	72	72	72	72	72	72	72	72	72	72	71	71	100	100	100		79	48	48	54	46	44	44	45	44	44	42	41	35	34	41	41	38	35	18	35	34	35	33	38	42
Methylomonas sp. FW.001	*Methylomonas* sp. FW.001	65	65	65	64	65	65	65	65	65	66	65	65	65	65	65	65	66	66	65	65	65	65	65	64	63	64	65	65	65	64	63	64	64	65	56	56	58	51	49	60	63	63	64	64	64	64	63	72	72	73	72	72	72	73	72	72	72	72	72	72	72	72	100	100	100	100		51	49	57	48	47	46	48	46	46	45	43	39	36	43	44	40	37	20	36	35	37	35	41	46
Methylomonas lenta R-45370	*Methylomonas lenta* R-45370	67	66	67	66	66	66	66	66	67	67	67	67	67	66	66	66	66	67	66	66	65	65	66	64	64	66	65	66	66	66	63	64	65	67	56	57	59	52	49	61	63	63	64	65	65	64	63	74	74	74	74	74	74	74	74	72	73	72	73	73	72	72	72	72	72	72	72		62	63	52	51	51	52	46	47	49	47	31	35	41	41	43	38	20	39	38	37	31	40	41
Methylomonas methanica MC09	*Methylomonas* sp. MC09	66	66	67	66	67	66	66	66	67	67	67	67	67	67	67	66	66	67	66	66	66	65	67	64	64	66	66	66	66	66	64	65	65	65	56	57	59	52	49	61	63	64	64	65	65	64	64	74	74	74	74	74	74	75	74	73	73	73	74	73	72	72	72	72	72	72	73	79		62	52	49	48	49	46	46	46	45	30	35	39	40	42	38	20	39	38	38	29	39	40
Methylomonas sp. FW.007	*Methylomonas* sp. FW.007	66	66	67	66	66	66	66	66	66	67	67	67	67	66	66	66	66	67	66	66	65	65	66	65	64	66	66	66	65	66	64	65	65	66	57	57	59	52	49	61	63	64	64	65	65	64	64	75	75	75	75	75	75	75	75	74	73	73	73	74	73	73	72	72	72	72	73	78	78		53	52	52	52	48	49	49	46	34	35	42	43	42	39	21	41	39	39	33	40	43
Methylomarinum vadi IT-4^{T}	*Methylomarinum vadi* IT-4^{T}	67	67	67	66	67	67	67	67	68	68	68	68	68	68	68	68	68	68	66	66	66	66	67	65	65	66	66	66	66	66	64	65	66	67	57	57	60	52	49	63	65	65	65	65	65	65	64	68	68	68	68	68	68	68	68	67	67	68	67	67	67	67	67	67	67	67	67	67	68	68		53	53	56	47	48	47	46	34	40	41	42	41	37	20	40	38	39	31	46	47
Methylomicrobium alcaliphilum 20Z^{T}	*Methylotuvimicrobium alcaliphilum* 20Z^{T}	68	68	68	68	68	68	68	68	68	68	70	70	69	69	69	69	68	69	67	67	66	66	68	66	65	67	67	67	67	67	64	66	66	68	57	57	60	52	49	63	64	66	66	65	66	66	65	65	66	65	65	65	65	65	65	65	65	65	65	65	65	64	64	64	64	65	65	65	65	67		78	70	49	49	48	47	34	37	40	41	41	36	20	40	39	39	31	44	44
"*Methylomicrobium buryatense*" 5G	"*Methylotuvimicrobium buryatense*" 5G	68	68	69	68	69	68	68	68	69	69	70	70	69	69	69	69	69	69	67	67	66	66	68	66	65	67	68	67	67	67	65	66	66	67	57	57	60	52	49	63	64	66	66	65	65	66	65	66	66	66	66	66	66	66	66	66	65	66	66	66	65	65	65	65	65	65	65	66	66	66	67	96		69	47	47	47	47	33	36	40	41	41	38	20	40	39	39	30	43	43
Methylomicrobium kenyense AMO1^{T}	*Methylotuvimicrobium kenyense* AMO1^{T}	68	68	68	67	68	68	68	68	68	69	70	70	69	69	69	69	69	69	67	67	66	66	68	66	65	67	67	67	67	67	65	66	66	67	57	57	60	52	50	63	64	66	66	66	66	66	65	66	66	66	66	66	66	66	66	66	66	66	66	66	65	65	65	65	65	65	65	65	66	66	67	88	88		48	49	48	47	34	37	42	42	41	37	20	40	39	39	32	44	44
Methylomicrobium agile ATCC 35068^{T}	*Methylomicrobium agile* ATCC 35068^{T}	70	70	71	70	70	70	70	70	71	71	72	72	71	70	70	70	69	70	69	69	68	68	70	67	67	68	69	69	68	69	66	68	69	69	57	57	59	52	50	62	63	68	68	67	67	67	66	66	66	66	66	66	66	66	66	65	66	65	66	66	65	65	64	65	64	64	65	65	65	65	66	66	66	67		91	61	58	34	36	42	42	41	38	19	41	39	42	32	41	41
Methylomicrobium album BG8^{T}	*Methylomicrobium album* BG8^{T}*	70	70	71	70	71	70	70	70	71	71	72	72	71	70	70	70	69	70	69	69	68	68	70	67	67	68	69	69	68	69	66	68	69	69	57	57	59	52	49	62	63	68	68	67	67	67	66	66	66	66	66	66	66	66	66	65	66	65	65	66	65	65	65	65	64	64	65	65	65	65	66	66	66	67	99		62	59	34	36	42	42	42	39	19	41	40	42	32	41	42
Methylosarcina lacus LW14^{T}	*Methylomicrobium lacus* LW14^{T}	71	71	71	70	71	71	71	71	71	71	72	72	71	70	70	70	70	70	69	69	69	69	71	68	67	69	69	69	69	69	67	68	69	70	57	57	60	52	50	62	63	68	68	67	67	67	66	67	67	67	66	67	66	66	67	66	66	66	66	66	66	66	65	65	65	65	65	65	66	65	66	67	67	67	82	82		58	35	36	42	43	41	38	19	40	39	40	32	40	41
Methylosarcina fibrata AML-C10^{T}	*Methylosarcina fibrata* AML-C10^{T}	71	71	72	71	72	72	71	71	71	72	74	74	73	70	70	70	71	71	69	70	69	69	71	68	67	69	69	69	70	70	66	68	70	70	56	57	59	51	49	62	63	68	69	68	67	67	67	65	65	66	65	65	65	65	65	65	65	65	65	65	65	65	65	65	65	65	65	65	65	65	66	67	67	67	73	73	73		33	37	41	41	42	37	19	42	40	41	30	41	41
Methylococcaceae bacterium UBA1147	*Methylococcaceae* bacterium UBA1147	60	60	60	60	60	60	60	59	60	61	60	60	60	61	61	61	61	61	60	60	60	60	60	59	59	59	60	59	60	60	59	59	60	60	56	57	59	52	50	59	59	59	60	60	59	60	59	59	59	59	59	59	59	59	59	60	60	60	60	60	59	59	59	59	59	59	59	59	60	59	60	60	60	60	60	60	60	60		37	38	38	33	29	16	30	29	31	30	36	41
Methylococcaceae bacterium UBA2778	*Methylococcaceae* bacterium UBA2778	62	61	62	61	62	62	62	61	61	62	62	62	61	62	62	62	62	62	60	60	61	60	61	60	60	60	60	60	61	60	59	60	60	61	57	58	61	52	50	61	60	60	60	60	60	61	60	60	60	60	60	60	60	60	60	60	60	60	60	60	60	60	60	60	60	60	60	60	60	60	62	61	61	61	61	61	61	61	63		38	39	36	32	17	35	34	34	29	40	42
Methylococcus capsulatus Bath	*Methylococcus capsulatus* Bath	59	59	59	58	59	59	59	58	59	59	59	60	59	59	59	59	59	60	59	59	59	59	60	59	59	59	59	59	59	59	59	59	59	59	56	57	59	53	50	58	57	59	59	59	59	60	59	59	59	59	59	59	59	59	59	59	59	60	60	60	60	59	59	59	59	59	59	58	59	59	59	59	59	60	60	60	60	60	60	61		91	53	48	22	48	46	48	43	43	45
Methylococcus capsulatus Texas^{T}	*Methylococcus capsulatus* Texas^{T}	59	59	59	58	59	59	59	58	59	60	59	60	59	59	59	60	59	60	59	59	59	59	60	59	59	59	59	59	59	59	59	59	59	59	56	57	59	53	50	58	57	58	59	59	59	60	59	59	59	59	59	59	59	59	59	59	59	60	60	60	60	59	59	59	59	59	59	58	59	59	59	59	59	60	60	60	60	60	60	61	99		53	48	22	49	46	48	43	43	45
Methyloterricola oryzae 73a^{T}	*Methyloterricola oryzae* 73a^{T}	59	59	60	59	59	59	60	59	60	60	60	60	60	60	60	60	60	60	60	60	60	59	60	59	59	59	59	59	59	59	59	59	59	59	56	57	59	52	49	58	58	59	59	59	59	60	59	59	59	59	59	59	59	59	59	59	59	59	59	59	60	59	59	59	59	59	59	58	59	58	59	59	59	59	60	60	60	60	60	61	66	66		48	22	48	47	46	37	41	43
Methylomagnum ishizawai 175	*Methylomagnum ishizawai* 175	59	59	59	58	59	59	59	58	59	59	60	60	59	59	59	59	59	59	59	59	59	59	59	59	59	59	59	59	59	59	58	59	58	58	56	56	58	51	49	58	57	58	59	59	59	59	59	59	59	59	59	59	59	59	59	59	59	59	59	59	59	59	59	59	59	59	59	58	58	58	59	59	59	59	60	60	60	60	60	61	65	65	65		20	45	44	45	36	37	38
Methylogaea oryzae JCM 16910^{T}	*Methylogaea oryzae* JCM 16910^{T}	60	60	60	60	60	60	60	60	60	60	61	60	60	60	60	60	61	61	61	60	61	61	61	60	60	60	60	60	60	60	59	60	60	59	57	58	59	54	50	59	59	60	60	60	60	60	60	60	61	60	61	61	61	61	61	61	61	61	61	61	61	61	60	60	60	60	60	60	60	60	60	60	60	60	60	60	61	61	60	62	64	64	64	63		20	19	20	17	19	20
Methylocaldum sp. 14B	*Methylocaldum* sp. 14B	59	59	59	59	59	59	59	59	59	60	60	61	59	60	60	60	60	61	59	59	60	59	60	59	59	59	59	59	59	59	59	59	59	59	57	57	60	51	49	59	57	59	59	59	59	60	59	59	59	59	59	59	59	59	59	59	59	59	59	59	59	59	59	59	59	59	59	58	58	59	59	59	59	59	60	60	59	60	60	61	66	67	66	69	64		82	65	33	41	40
Methylocaldum sp. SAD2	*Methylocaldum* sp. SAD2	59	59	59	59	59	59	59	59	59	60	60	60	59	60	60	60	60	61	60	59	59	59	60	59	59	59	59	59	59	59	59	59	59	59	56	57	60	52	49	59	58	59	59	59	59	60	59	59	59	59	59	59	59	59	59	59	59	59	59	59	59	59	59	59	59	59	59	58	59	59	59	59	59	59	60	60	60	60	60	61	66	66	66	69	64	98		64	32	40	39
Methylocaldum szegediense O-12	*Methylocaldum szegediense* O-12	60	59	60	59	60	59	59	59	60	60	60	60	60	60	60	60	60	61	60	60	60	60	60	59	59	59	60	59	59	60	59	59	59	59	57	57	60	52	50	59	58	59	59	59	59	60	59	59	59	59	59	59	59	59	59	59	59	59	59	59	59	59	59	59	59	59	59	59	59	60	59	60	60	60	60	60	60	60	60	62	67	67	66	69	64	84	84		34	42	41
Methylococcus sp. UBA6136	*Methylococcaceae* bacterium UBA6136	59	59	59	58	59	59	59	58	59	59	58	58	58	59	59	59	59	59	59	59	59	59	59	58	59	58	59	59	58	58	58	58	58	58	55	56	59	51	49	58	57	58	58	59	58	59	58	59	59	59	59	59	59	59	59	59	59	59	59	59	59	59	59	59	59	59	59	58	58	59	59	59	59	59	59	59	59	59	58	60	63	63	63	67	63	66	66	66		32	34
Methylohalobius crimeensis 10Ki^{T}	*Methylohalobius crimeensis* 10Ki^{T}	57	57	57	57	57	57	57	57	57	58	59	58	58	58	58	58	58	58	58	58	58	57	58	58	58	58	58	58	58	57	57	57	57	57	55	56	58	51	49	57	57	58	57	57	57	58	57	57	57	57	57	57	58	58	57	57	58	58	58	58	58	57	57	57	57	57	57	57	58	57	58	58	58	58	58	58	58	58	59	60	60	60	59	59	60	60	60	60	59		61
Methylothermaceae bacterium B42	*Methylothermaceae* bacterium B42	58	58	58	58	58	58	58	57	58	59	59	59	59	59	59	59	59	59	59	58	59	59	59	58	58	59	59	59	59	59	58	58	58	58	56	57	59	52	50	58	58	58	58	58	58	59	58	58	58	58	58	58	58	58	58	58	58	58	58	58	58	58	58	58	58	58	58	58	58	58	59	59	59	59	59	59	59	58	60	61	61	61	60	60	61	61	61	61	59	69	

Values are shown for AAI (green–purple, lower left) and POCP (yellow–blue, upper right). Boxed regions indicate inferred genus clusters with at least two members based on AAI comparisons, as well as monophyly in the genome-based phylogeny. POCP was not used to infer genera. Asterisks after reclassified names indicate later heterotypic synonyms of their senior counterparts in the same species clusters as shown in Figure 2.

Supplementary Table S4. dDDH and ANI from pairwise whole-genome comparisons.

			Average nucleotide identity (ANI)																																																																																									
	Previous name	Name after reclassification	*Methylobacter* sp. UBA2091	*Methylobacter* sp. UBA6696	*Methylobacter tundripaludum* OWC-DMM	*Methylobacter* sp. UBA6701	*Methylobacter* sp. UBA6712	*Methylobacter tundripaludum* 21/22	*Methylobacter tundripaludum* 31/32	*Methylobacter tundripaludum* UBA6706	*Methylobacter tundripaludum* SV96^{T}	*Methylobacter tundripaludum* OWC-G53F	*Methylococcaceae* bacterium NSO1	*Methylococcaceae* bacterium NSP1-1	*Methylococcaceae* bacterium NSM2-1	*Methylobacter whittenburyi* ACM 3310^{T}	*Methylobacter whittenburyi* UCM-B-3033	*Methylobacter marinus* A45^{T}	*Methylobacter* sp. BBA5.1	*Methylobacter luteus* IMV-B-3098	*Methylobacter* sp. BC.3.94	*Methylovulum miyakonense* HT12^{T}	*Methylovulum psychrotolerans* HV10-M2	*Methylovulum psychrotolerans* Sph1^{T}	*Methylococcaceae* bacterium UBA6146	*Methylococcaceae* bacterium UBA659	*Methylococcaceae* bacterium UBA662	*Methylococcaceae* bacterium UBA4132	*Methylococcaceae* bacterium UBA5071	*Methylococcaceae* bacterium UBA3127	*Crenothrix* sp. D3	*Methylococcaceae* bacterium NSP1-2	"*Methylocucumis oryzae*" Sn10-6^{T}	*Crenothrix polyspora* RSM_CP1	*Crenothrix polyspora* RSM_CP2	*Methylobacter* sp. BC.3.60	*Methylococcaceae* bacterium UBA1114	*Methylococcaceae* bacterium UBA975	*Methylococcaceae* bacterium UBA2780	*Methylococcaceae* bacterium TMED282	*Methylococcaceae* bacterium TMED69	*Methylococcales* bacterium OPU3_GD_OMZ	*Methyloprofundus sedimenti* WF1^{T}	*Methylococcaceae* bacterium UBA7658	*Methyloglobulus morosus* KoM1^{T}	*Methylobacter* sp. WM.3.3	*Methylomonas* sp. FW.017	*Methylobacter* sp. DS2.3.46	*Methylobacter* sp. DS3.3.25	*Methylomonas methanica* R-45363	*Methylomonas methanica* R-45371	*Methylomonas* sp. 11b	*Methylomonas* sp. MK1	*Methylomonas* sp. Kb3	*Methylomonas* sp. LW13	"*Methylomonas denitrificans*" FJG1^{T}	*Methylomonas methanica* NCIMB 11130^{T}	*Methylomonas koyamae* LM6	*Methylomonas koyamae* R-49807	*Methylomonas* sp. DH-1	*Methylomonas koyamae* JCM 16701^{T}	*Methylomonas koyamae* R-45378	*Methylomonas koyamae* R-45383	*Methylomonas* sp. LWB	*Methylomonas* sp. BC.006	*Methylomonas* sp. DS2.3.37	*Methylomonas* sp. DS1.3.54	*Methylomonas* sp. DS1.022	*Methylomonas* sp. FW.001	*Methylomonas lenta* R-45370	*Methylomonas methanica* MC09	*Methylomonas* sp. FW.007	*Methylomarinum vadi* IT-4^{T}	*Methylomicrobium alcaliphilum* 20Z^{T}	"*Methylomicrobium buryatense*" 5G	*Methylomicrobium kenyense* AMO1^{T}	*Methylomicrobium agile* ATCC 35068^{T}	*Methylomicrobium album* BG8^{T}	*Methylosarcina lacus* LW14^{T}	*Methylosarcina fibrata* AML-C10^{T}	*Methylococcaceae* bacterium UBA1147	*Methylococcaceae* bacterium UBA2778	*Methylococcus capsulatus* Bath	*Methylococcus capsulatus* Texas^{T}	*Methyloterricola oryzae* 73a^{T}	*Methylomagnum ishizawai* 175	*Methylogaea oryzae* JCM 16910^{T}	*Methylocaldum* sp. 14B	*Methylocaldum* sp. SAD2	*Methylocaldum szegediense* O-12	*Methylococcus* sp. UBA6136	*Methylohalobius crimeensis* 10Ki^{T}	*Methylothermaceae* bacterium B42
Digital DNA–DNA hybridization (dDDH)	*Methylobacter* sp. UBA2091	*Methylobacter* sp. UBA2091		100	97	96	96	95	95	96	96	85	77	77	78	75	75	75	75	75	72	72	72	71	74	70	70	71	71	71	72	72	69	72	73	73	64	64	65	62	62	66	68	71	71	70	70	70	70	70	70	70	70	70	70	70	70	69	69	69	69	69	69	69	69	69	69	69	69	69	70	69	70	70	70	70	71	71	72	72	65	67	66	66	66	66	67	66	66	66	66	65	65
	Methylobacter sp. UBA6696	*Methylobacter* sp. UBA6696	99		97	96	96	96	95	96	96	85	77	77	78	75	75	75	75	75	72	72	72	72	74	70	70	72	72	72	72	72	68	72	73	73	64	64	65	63	62	66	68	71	71	70	70	70	70	70	70	70	70	70	70	70	70	69	69	69	69	69	69	69	69	69	69	69	69	69	70	69	70	70	70	70	71	71	72	72	65	67	66	66	66	66	67	66	66	66	66	65	65
	Methylobacter tundripaludum OWC-DMM	*Methylobacter* sp. OWC-DMM	72	72		96	97	96	96	96	96	85	77	77	78	75	75	75	75	75	72	72	72	72	74	70	70	72	72	72	72	72	69	72	73	73	64	64	65	63	62	66	68	71	71	70	70	70	70	70	70	70	70	70	70	70	70	70	70	70	70	70	69	69	69	69	69	69	69	69	70	69	70	70	70	70	71	71	72	72	65	67	66	66	66	66	67	66	66	66	66	65	65
	Methylobacter sp. UBA6701	*Methylobacter* sp. UBA6701	69	69	72		100	96	95	96	96	85	77	77	78	74	74	74	75	75	72	72	71	71	74	70	70	71	71	71	71	72	68	72	73	73	64	64	65	62	62	66	68	70	71	70	70	70	70	70	70	70	70	70	70	70	70	69	69	69	69	69	69	69	69	69	69	69	69	69	69	69	69	70	70	70	71	71	72	72	65	66	66	66	66	66	67	66	66	66	66	65	65
	Methylobacter sp. UBA6712	*Methylobacter* sp. UBA6712	69	69	73	99		96	96	96	96	85	77	77	78	75	75	75	75	75	72	72	72	72	74	70	70	72	72	72	72	72	69	72	73	73	64	64	65	63	62	66	68	71	71	70	70	70	70	70	70	70	70	70	70	70	70	69	69	69	70	69	69	69	69	69	69	69	69	69	70	69	70	70	70	70	71	71	72	72	65	67	66	66	66	66	67	66	66	66	66	65	65
	Methylobacter tundripaludum 21/22	*Methylobacter* sp. 21/22	64	65	65	64	65		98	96	96	85	77	77	78	75	75	75	75	75	72	72	72	72	74	70	70	72	72	72	72	72	69	72	73	73	64	64	65	63	62	66	68	71	71	70	70	70	70	70	70	70	70	70	70	70	70	70	70	70	70	70	69	69	69	69	69	69	69	70	70	69	70	70	70	70	71	71	72	72	65	67	66	66	66	66	67	66	66	66	66	65	65
	Methylobacter tundripaludum 31/32	*Methylobacter* sp. 31/32	64	64	65	64	65	84		96	96	85	77	77	78	75	75	75	75	75	72	72	72	71	74	70	70	72	72	72	72	72	69	72	73	73	64	64	65	63	62	66	68	71	71	70	70	70	70	70	70	70	70	70	70	70	70	70	70	70	70	70	69	69	69	69	69	69	69	69	70	69	70	70	70	70	71	71	72	72	65	67	66	66	66	66	67	66	66	66	66	65	65
	Methylobacter tundripaludum UBA6706	*Methylobacter* sp. UBA6706	66	66	67	66	66	69	69		96	85	77	77	78	75	75	75	75	75	72	72	71	71	74	70	69	71	71	71	71	72	68	71	73	73	64	64	64	62	62	66	67	70	71	70	70	70	70	70	70	70	70	70	70	70	70	70	69	69	69	69	69	69	68	68	68	69	69	69	70	69	69	70	70	70	71	71	72	72	65	67	66	65	65	66	67	66	66	65	66	65	65
	Methylobacter tundripaludum SV96^{T}	*Methylobacter tundripaludum* SV96^{T}	66	66	67	67	67	67	67	69		85	77	77	78	75	75	75	75	75	72	72	72	72	74	70	70	72	72	72	72	72	69	72	73	73	64	65	65	63	62	66	68	71	71	70	70	70	70	70	70	70	70	70	70	70	70	70	69	69	70	70	70	69	69	69	69	69	69	70	70	69	70	70	70	70	71	71	72	72	65	67	66	66	66	66	67	66	66	66	66	65	65
	Methylobacter tundripaludum OWC-G53F	*Methylobacter* sp. OWC-G53F	30	30	30	31	31	30	30	30	30		76	76	77	75	75	75	75	75	72	73	72	72	74	71	70	71	71	71	71	72	68	71	72	72	64	64	65	63	62	66	68	71	71	70	70	70	70	70	70	70	70	70	70	70	70	70	70	70	70	70	70	70	69	69	69	69	69	69	70	69	70	70	70	71	72	72	72	73	66	67	66	66	66	66	67	66	66	66	66	65	65
	Methylococcaceae bacterium NSO1	*Methylobacter* sp. NSO1	22	22	22	22	22	22	22	21	22	21		97	88	74	74	74	74	75	72	72	71	71	74	69	69	73	73	73	73	74	69	72	73	72	65	64	65	63	62	67	69	70	71	70	70	70	69	69	69	68	69	69	69	68	68	68	68	68	68	68	68	68	67	67	67	68	68	69	69	69	69	70	70	69	69	69	70	71	64	65	64	64	64	64	65	65	65	64	64	64	64
	Methylococcaceae bacterium NSP1-1	*Methylobacter* sp. NSP1-1	22	22	22	21	22	22	22	21	22	21	77		89	74	74	74	74	74	72	72	71	71	74	69	69	73	73	73	73	74	69	72	73	73	64	64	65	63	62	67	69	70	71	69	70	70	69	69	69	69	69	69	69	69	69	68	68	68	68	68	68	68	68	68	68	68	68	69	69	69	69	69	70	69	69	69	70	71	65	65	65	64	64	65	65	65	65	65	65	64	65
	Methylococcaceae bacterium NSM2-1	*Methylobacter* sp. NSM2-1	22	22	23	22	23	22	22	22	23	21	35	37		74	74	74	74	75	72	72	71	71	74	70	69	73	73	73	73	74	70	72	73	73	65	65	66	63	62	67	69	70	71	70	70	70	70	69	69	69	69	69	69	69	69	68	68	68	68	68	68	68	68	68	68	68	69	69	69	69	69	70	70	69	69	70	70	71	65	66	65	65	65	65	66	65	65	65	65	64	65
	Methylobacter whittenburyi ACM 3310^{T}	*Methylobacter whittenburyi* ACM 3310^{T}*	20	20	21	20	21	21	21	20	21	21	22	20	20		100	99	98	84	71	72	71	71	72	70	69	69	70	69	70	70	68	70	71	70	64	64	65	63	62	66	67	69	70	70	70	70	70	70	70	70	70	70	70	70	70	70	70	70	70	70	70	70	69	69	69	69	69	70	70	69	71	71	71	71	72	72	72	73	66	67	67	67	67	67	68	67	67	66	66	66	65
	Methylobacter whittenburyi UCM-B-3033	*Methylobacter marinus* UCM-B-3033	20	20	21	20	21	21	21	20	21	21	22	20	20	100		99	98	84	71	72	71	71	72	70	69	69	70	69	70	70	68	70	71	70	64	64	65	63	62	66	67	69	70	70	70	70	70	70	70	70	70	70	70	70	70	70	70	70	70	70	70	70	69	69	69	69	69	70	70	69	71	71	71	71	72	72	72	73	66	67	67	67	67	67	68	67	67	66	66	66	65
	Methylobacter marinus A45^{T}	*Methylobacter marinus* A45^{T}	20	20	21	20	20	21	21	20	21	21	22	20	20	89	89		99	85	71	71	71	71	72	70	69	69	70	70	69	70	68	70	71	71	64	64	65	63	62	66	67	69	70	70	70	70	70	70	70	69	70	69	69	70	70	70	70	70	70	70	69	70	69	69	69	69	69	69	70	69	71	70	70	71	72	72	72	73	66	67	67	67	67	67	68	67	67	67	66	66	65
	Methylobacter sp. BBA5.1	*Methylobacter marinus* BBA5.1	20	20	21	20	21	21	21	21	21	21	22	20	20	88	88	90		85	71	71	71	71	72	70	69	69	70	70	69	70	68	70	71	70	64	64	65	62	62	66	67	69	70	71	70	70	70	70	70	70	70	69	70	70	70	70	70	70	70	70	69	69	69	69	69	69	69	69	70	69	70	70	70	71	72	72	72	74	66	67	67	67	67	67	68	67	67	66	66	66	65
	Methylobacter luteus IMV-B-3098	*Methylobacter luteus* IMV-B-3098	21	21	21	21	21	22	21	20	22	21	21	20	20	29	29	29	29		71	72	71	71	72	70	69	70	70	70	70	70	68	70	71	71	64	64	65	62	62	66	67	69	70	70	70	70	70	70	70	70	70	70	70	70	70	70	70	70	70	70	70	70	69	69	69	69	69	69	70	70	69	70	71	70	71	72	72	72	73	65	67	66	67	67	67	67	67	67	66	66	66	65
	Methylobacter sp. BC.3.94	*Methylovulum miyakonense* BC.3.94	19	19	19	19	19	19	19	19	19	20	19	19	19	19	19	19	19	19		97	76	76	74	71	71	71	71	71	70	71	69	70	71	70	64	64	65	63	62	66	67	69	70	69	69	70	69	70	70	69	70	69	70	70	70	69	69	69	69	69	69	69	69	69	69	69	69	69	69	69	69	69	69	69	70	70	70	71	65	66	66	66	66	66	67	66	66	65	65	65	65
	Methylovulum miyakonense HT12^{T}	*Methylovulum miyakonense* HT12^{T}	19	19	20	19	19	20	20	18	20	20	20	19	19	20	20	19	19	20	73		77	77	74	71	71	70	70	71	70	71	69	70	71	70	64	64	65	63	62	66	67	69	70	69	69	70	69	69	70	70	70	70	70	70	70	69	69	69	70	70	69	69	69	69	69	69	69	69	69	69	69	69	69	69	70	70	71	71	65	66	66	66	66	67	67	66	66	66	66	65	65
	Methylovulum psychrotolerans HV10-M2	*Methylovulum psychrotolerans* HV10-M2	19	18	19	18	19	19	20	19	20	19	18	18	18	19	19	19	18	19	22	22		98	73	71	70	70	70	70	70	71	69	70	70	70	64	65	65	63	62	66	67	69	70	69	69	70	69	69	69	69	69	69	69	69	69	69	69	69	69	69	69	69	69	68	68	69	69	68	69	69	69	69	69	69	70	70	71	70	65	66	66	66	66	66	67	66	65	66	65	65	65
	Methylovulum psychrotolerans Sph1^{T}	*Methylovulum psychrotolerans* Sph1^{T}	19	19	20	19	19	20	20	19	20	19	19	18	18	19	19	19	18	19	22	22	83		73	71	70	70	70	70	70	71	69	70	70	70	64	64	65	63	62	66	67	69	70	69	69	70	69	69	69	69	69	69	69	69	69	69	69	69	69	69	69	69	69	68	68	69	69	68	69	69	69	69	69	69	70	70	70	70	65	66	66	66	66	66	67	66	65	65	65	65	65
	Methylococcaceae bacterium UBA6146	*Methylovulum* sp. UBA6146	20	20	20	20	20	19	19	19	20	19	19	19	20	19	19	19	19	18	21	21	20	20		71	71	71	71	71	71	72	69	71	72	71	64	64	65	63	62	66	67	69	70	70	70	70	70	69	69	69	69	69	69	69	69	69	68	68	69	69	68	68	68	68	68	68	69	69	69	69	69	69	69	69	70	70	70	71	65	66	65	65	65	65	66	65	65	65	65	65	65
	Methylococcaceae bacterium UBA659	*Methylococcaceae* bacterium UBA659	17	17	18	18	18	18	18	18	18	18	18	17	16	18	18	18	17	17	20	20	18	18	19		77	68	68	68	68	69	68	69	69	69	63	64	64	63	61	65	66	68	69	69	69	69	69	69	68	68	68	68	68	68	68	69	69	69	69	69	68	69	69	69	68	69	69	68	69	68	69	68	68	69	70	70	70	70	65	66	66	66	66	66	67	66	66	65	66	65	65
	Methylococcaceae bacterium UBA662	*Methylococcaceae* bacterium UBA662	17	17	17	17	17	17	17	18	17	17	16	17	20	17	17	17	17	17	19	19	18	18	19	22		68	68	68	68	68	67	68	69	68	63	64	64	62	62	65	66	68	68	68	69	69	68	68	68	68	68	68	68	68	68	68	68	68	68	68	68	69	68	68	68	68	69	67	68	68	68	68	68	68	69	69	69	69	65	66	66	66	66	66	67	65	65	65	65	65	65
	Methylococcaceae bacterium UBA4132	*Methylococcaceae* bacterium UBA4132	19	18	19	19	19	19	19	18	19	19	20	19	19	20	20	20	20	19	17	17	19	19	19	16	16		100	100	81	82	70	71	71	71	65	65	65	63	62	66	68	69	69	68	68	68	68	68	68	68	68	68	68	68	68	67	67	67	67	67	66	67	67	67	67	67	67	68	67	68	67	68	68	68	67	67	68	68	64	64	63	63	63	64	64	64	64	64	64	63	64
	Methylococcaceae bacterium UBA5071	*Methylococcaceae* bacterium UBA5071	19	18	19	19	19	19	19	18	19	19	20	19	19	19	19	19	19	19	18	18	19	19	19	16	16	100		100	81	82	70	71	71	71	64	65	65	63	63	66	68	69	69	68	68	68	68	68	68	68	68	68	68	68	68	67	67	67	67	67	67	67	67	67	67	67	67	68	68	68	67	68	68	68	67	67	68	68	64	64	63	63	63	64	64	64	64	64	64	63	64
	Methylococcaceae bacterium UBA3127	*Methylococcaceae* bacterium UBA3127	19	18	19	19	19	19	19	18	19	19	20	19	19	19	19	19	19	19	18	18	18	18	19	15	17	100	100		81	82	70	71	71	71	65	65	65	63	62	67	68	69	69	68	68	69	68	68	68	68	68	68	68	68	68	67	67	67	67	67	67	67	67	67	67	67	67	68	67	68	67	68	68	68	67	67	68	68	64	64	64	64	63	64	64	64	64	64	64	63	64
	Crenothrix sp. D3	*Methylococcaceae* bacterium D3	19	19	20	18	20	20	20	18	20	19	20	19	20	19	19	19	19	19	18	19	19	19	19	16	17	25	25	25		82	70	71	71	71	64	65	65	63	62	67	68	69	69	68	68	68	68	68	68	68	68	68	68	68	68	67	67	67	67	67	67	67	67	66	66	67	67	68	67	68	67	68	68	68	67	67	68	68	64	64	63	63	63	64	64	64	64	64	64	63	64
	Methylococcaceae bacterium NSP1-2	*Methylococcaceae* bacterium NSP1-2	20	20	20	19	20	20	20	19	21	20	24	24	23	19	19	19	19	20	19	19	19	19	19	16	17	26	26	26	27		70	72	72	71	65	65	65	63	63	67	68	69	70	68	68	69	68	68	68	68	68	68	68	68	68	67	67	67	67	67	67	67	67	67	67	67	67	68	68	68	68	68	68	68	67	67	68	68	64	64	63	63	64	64	64	64	64	64	64	63	64
	"*Methylocucumis oryzae*" Sn10-6^{T}	"*Methylocucumis oryzae*" Sn10-6^{T}	18	18	19	17	18	19	19	17	19	19	19	18	17	18	18	18	17	19	19	19	18	18	18	17	17	17	18	18	18	18		69	69	69	64	65	65	63	63	66	67	67	67	67	67	68	67	67	67	67	67	67	67	67	67	66	66	66	66	66	66	66	66	66	66	66	66	67	67	67	67	67	67	67	66	66	67	67	63	64	63	64	63	64	64	64	64	64	63	63	64
	Crenothrix polyspora RSM_CP1	*Crenothrix polyspora* RSM_CP1	18	18	19	18	19	20	20	18	19	19	18	18	18	20	20	19	19	18	19	20	19	19	19	17	17	18	19	18	20	19	18		80	70	64	65	65	63	62	66	68	69	70	68	69	69	68	68	68	68	68	68	68	68	68	68	67	68	68	68	67	68	67	67	67	67	67	68	68	68	68	68	68	68	68	68	69	69	64	65	65	65	65	65	65	65	65	65	65	64	65
	Crenothrix polyspora RSM_CP2	*Crenothrix* sp. RSM_CP2	19	19	19	19	20	20	19	19	19	19	19	19	19	18	18	19	18	19	19	19	19	19	19	17	16	18	19	18	19	19	17	26		70	64	65	65	63	62	66	67	69	71	69	69	69	69	68	68	68	68	68	68	68	68	68	68	68	68	68	67	68	68	68	68	68	68	68	68	68	68	69	68	69	69	69	69	70	65	65	64	64	65	65	65	65	65	65	65	64	65
	Methylobacter sp. BC.3.60	*Methylococcaceae* bacterium BC.3.60	18	18	18	18	18	18	18	18	18	18	19	18	19	17	17	17	17	17	17	17	18	17	17	16	19	18	18	18	19	19	17	18	17		64	64	64	62	62	66	67	69	69	68	69	69	68	69	69	68	69	69	68	68	68	67	67	67	67	67	67	67	67	67	67	67	67	69	68	68	67	68	68	68	68	68	69	69	64	65	63	64	64	64	64	64	64	64	64	63	64
	Methylococcaceae bacterium UBA1114	*Methylococcaceae* bacterium UBA1114	15	16	19	15	16	31	31	33	31	18	31	31	28	19	19	19	20	20	25	26	27	27	19	20	15	14	15	16	15	15	16	20	17	32		70	65	62	62	64	65	64	64	64	63	64	63	64	64	64	64	63	63	64	64	63	63	63	63	63	63	63	63	63	63	63	63	64	64	64	64	64	64	64	63	63	63	63	63	63	62	62	62	63	63	63	62	63	63	62	63
	Methylococcaceae bacterium UBA975	*Methylococcaceae* bacterium UBA975	17	18	16	15	16	21	20	17	20	18	16	26	18	20	20	25	25	23	24	29	18	20	21	26	28	16	18	17	20	15	18	23	34	14	16		65	62	61	64	65	64	64	64	64	64	64	64	64	64	64	64	64	64	64	64	64	64	64	64	64	64	64	64	64	64	64	64	64	64	64	64	64	64	64	64	63	64	64	63	63	63	63	63	63	63	63	63	63	63	63	63
	Methylococcaceae bacterium UBA2780	*Methylococcaceae* bacterium UBA2780	16	16	20	18	20	21	21	17	20	23	36	36	15	18	18	18	34	19	19	19	20	21	17	18	23	20	22	21	28	18	24	25	23	24	22	25		63	62	65	65	64	64	64	64	64	64	64	64	64	64	64	64	64	64	64	64	64	64	64	64	64	64	64	64	64	64	64	64	64	64	64	65	64	64	64	64	64	64	64	64	65	64	64	64	63	64	64	64	64	64	63	64
	Methylococcaceae bacterium TMED282	*Methylococcaceae* bacterium TMED282	67	67	43	67	67	40	37	67	40	37	35	6	6	27	27	27	65	25	64	35	37	35	64	78	53	6	64	66	38	53	25	49	79	6	6	23	56		64	63	63	63	63	62	62	63	62	62	62	62	62	62	62	62	62	63	62	62	63	62	62	62	62	62	62	62	63	62	62	63	63	63	63	62	63	62	62	62	62	62	62	62	62	62	62	62	62	62	62	62	62
	Methylococcaceae bacterium TMED69	*Methylococcaceae* bacterium TMED69	15	15	15	15	15	15	15	15	15	14	6	16	6	14	14	6	14	15	20	20	19	19	20	6	40	6	48	48	23	19	19	17	41	6	88	40	25	18		62	62	62	61	62	61	62	61	62	62	62	62	62	62	62	62	61	61	61	62	61	62	61	61	61	61	61	61	62	62	62	61	62	62	61	61	61	61	61	61	62	61	60	61	61	61	61	61	61	61	61	61
	Methylococcales bacterium OPU3_GD_OMZ	*Methylococcaceae* bacterium OPU3_GD_OMZ	16	17	17	17	17	17	17	17	18	18	17	15	16	18	18	18	17	17	17	19	19	20	16	35	25	16	17	17	18	16	18	18	16	15	17	19	16	14	24		67	66	66	65	65	66	65	65	65	65	66	65	66	65	65	65	65	65	65	65	65	65	64	64	64	64	65	66	66	66	65	66	66	66	65	65	65	65	64	64	63	63	63	63	63	63	63	63	63	63	64
	Methyloprofundus sedimenti WF1^{T}	*Methyloprofundus sedimenti* WF1^{T}	17	17	20	17	19	23	23	16	23	19	19	18	18	25	25	24	21	24	19	24	24	25	18	18	32	16	17	16	19	18	19	20	16	22	16	28	26	38	23	17		66	66	66	66	67	66	67	67	67	67	67	67	67	67	66	66	66	66	66	66	66	65	65	65	65	66	68	67	67	67	67	67	67	65	65	66	66	64	64	63	63	63	63	63	64	63	63	63	63	64
	Methylococcaceae bacterium UBA7658	*Methyloglobulus* sp. UBA7658	17	17	18	18	17	17	17	17	18	18	17	17	16	18	18	17	17	18	17	18	17	18	17	16	17	17	17	17	17	17	16	18	18	17	21	21	26	37	17	15	16		73	68	68	69	68	68	68	68	68	68	68	68	68	68	68	68	68	68	68	68	68	68	68	68	68	68	68	68	68	68	68	68	68	68	68	69	69	69	69	69	65	65	65	65	65	66	66	65	65	65	65	65	65
	Methyloglobulus morosus KoM1^{T}	*Methyloglobulus morosus* KoM1^{T}	18	18	19	18	18	19	18	18	19	18	18	18	19	18	18	17	17	18	18	18	17	17	18	17	18	17	17	17	17	18	17	20	18	16	19	17	20	17	22	16	17	19		68	69	69	69	68	68	68	68	68	68	68	68	68	68	68	68	68	68	68	68	68	68	68	68	68	68	68	68	68	68	68	68	68	68	68	69	69	70	70	65	65	65	65	65	66	66	65	65	65	65	65	65
	Methylobacter sp. WM.3.3	*Methylococcaceae* bacterium WM.3.3	18	18	19	18	18	19	19	18	19	18	17	19	17	17	17	17	19	18	18	18	17	17	17	17	17	17	18	17	17	18	16	17	17	16	16	28	16	53	18	14	16	18	19		99	100	99	69	69	68	69	69	68	69	69	68	68	68	68	69	68	68	68	68	68	75	68	68	68	68	68	68	68	68	69	69	69	69	64	66	65	65	66	66	66	65	65	65	65	65	65
	Methylomonas sp. FW.017	*Methylococcaceae* bacterium FW.017	18	18	18	18	18	18	19	18	18	18	18	21	19	17	17	17	17	16	17	17	17	17	17	17	19	17	18	17	17	17	16	17	18	16	16	14	16	53	6	14	17	16	18	99		99	98	69	69	69	69	69	69	69	69	69	69	69	69	69	68	68	68	68	68	74	68	68	69	68	68	68	68	68	69	69	69	69	64	66	65	65	65	66	66	66	65	65	65	65	65
	Methylobacter sp. DS2.3.46	*Methylococcaceae* bacterium DS2.3.46	18	18	18	18	18	18	18	18	18	18	18	18	18	17	17	17	18	17	17	17	17	18	17	17	17	17	17	17	17	17	17	17	17	16	16	17	17	53	18	15	17	18	19	98	99		99	69	70	69	69	69	69	69	69	69	69	69	69	69	68	68	68	68	68	73	68	68	69	68	69	68	69	69	69	69	69	70	65	66	66	66	66	66	67	66	66	66	66	65	65
	Methylobacter sp. DS3.3.25	*Methylococcaceae* bacterium DS3.3.25	18	18	17	18	17	18	18	17	18	19	17	19	18	17	17	17	18	17	17	17	17	18	17	16	17	16	17	16	17	17	17	17	18	17	21	15	16	53	15	14	17	16	19	94	92	93		69	69	68	68	68	69	69	69	68	68	68	68	68	69	68	68	68	68	75	68	68	68	68	68	68	68	68	69	69	69	69	65	66	66	66	66	66	67	66	66	66	65	65	65
	Methylomonas methanica R-45363	*Methylomonas* sp. R-45363	18	18	19	19	19	20	20	19	19	19	21	18	18	20	20	19	19	22	19	19	19	20	22	18	20	17	17	18	19	18	22	19	18	27	28	22	37	37	20	20	30	19	17	20	20	25	22		95	89	90	87	87	88	88	76	76	76	76	76	75	75	72	72	72	72	72	73	74	74	71	69	69	70	70	70	70	70	65	66	66	66	66	67	67	66	66	66	66	66	65
	Methylomonas methanica R-45371	*Methylomonas* sp. R-45371	20	19	18	19	19	21	21	23	20	19	20	19	19	20	20	19	20	21	19	20	20	20	17	18	18	17	18	17	20	18	20	19	16	25	28	31	37	37	20	20	28	18	17	22	19	29	22	63		90	90	87	87	88	88	76	76	76	76	76	75	75	72	72	72	72	72	73	74	74	70	69	69	70	70	70	70	70	65	66	66	67	66	67	67	66	66	66	66	65	65
	Methylomonas sp. 11b	*Methylomonas* sp. 11b	21	20	20	20	20	21	21	25	21	20	20	19	19	20	20	20	20	20	19	20	21	21	18	17	17	18	18	18	20	17	22	22	17	17	25	29	37	36	20	21	34	17	17	19	18	24	18	39	40		95	89	89	87	87	76	76	76	76	76	75	75	72	72	72	72	72	73	74	73	70	69	69	69	70	70	70	70	65	66	66	66	66	67	67	66	66	66	66	66	65
	Methylomonas sp. MK1	*Methylomonas* sp. MK1	18	18	19	18	19	20	20	18	19	20	21	18	18	20	20	19	19	20	19	20	21	21	19	17	17	18	18	18	20	17	21	21	18	30	25	29	34	36	20	22	27	17	19	23	18	26	19	40	40	60		89	89	87	87	76	76	76	76	76	75	75	72	72	72	72	72	73	74	73	70	69	69	69	70	70	70	70	65	66	66	67	66	67	68	66	66	66	66	66	65
	Methylomonas sp. Kb3	*Methylomonas* sp. Kb3	19	19	19	18	18	19	19	18	19	20	18	18	18	20	20	19	19	21	18	19	20	20	19	17	19	17	18	18	19	18	20	20	18	28	21	22	37	36	20	20	28	18	19	20	19	19	18	33	33	37	37		98	86	86	76	77	76	77	77	75	75	72	72	72	72	72	73	74	73	70	69	69	70	70	70	70	70	65	66	66	67	66	67	68	66	66	66	66	65	65
	Methylomonas sp. LW13	*Methylomonas* sp. LW13	19	19	19	18	19	20	20	18	20	20	18	18	18	20	20	19	19	21	18	19	20	20	19	17	19	17	18	18	20	18	19	20	18	18	22	22	38	36	19	20	31	18	19	18	19	20	19	33	34	38	37	80		86	86	76	77	76	77	77	75	75	72	72	72	72	72	73	74	73	70	69	69	69	70	70	70	70	65	66	67	67	66	67	68	66	66	66	66	66	65
	"*Methylomonas denitrificans*" FJG1^{T}	"*Methylomonas denitrificans*" FJG1^{T}*	19	19	21	19	19	20	20	19	20	19	23	20	18	19	19	19	20	20	18	20	19	19	20	18	17	18	18	18	19	18	23	23	17	19	29	24	32	38	21	20	34	17	17	19	21	21	20	35	35	33	34	32	32		100	76	77	76	77	76	75	75	72	72	72	72	73	73	74	74	71	69	69	70	70	70	70	70	65	66	67	67	66	67	68	66	66	66	66	66	65
	Methylomonas methanica NCIMB 11130^{T}	*Methylomonas methanica* NCIMB 11130^{T}	19	19	20	19	19	20	20	19	20	19	22	20	18	19	19	19	19	20	18	20	18	18	20	18	17	17	18	18	19	18	21	21	17	19	28	22	32	40	21	19	35	17	17	19	21	21	20	35	35	33	33	32	32	92		76	77	76	77	76	75	75	72	72	72	72	73	73	74	74	71	69	69	70	70	70	70	70	65	66	66	67	66	67	68	66	66	66	66	66	65
	Methylomonas koyamae LM6	*Methylomonas koyamae* LM6	18	18	19	18	18	20	20	18	20	19	20	19	18	20	20	19	19	19	18	19	19	20	18	17	18	18	18	19	21	18	18	21	16	16	26	34	32	37	20	19	30	18	18	17	18	18	17	21	21	21	21	22	22	21	21		99	97	97	96	76	76	73	73	73	73	73	72	74	72	71	69	69	70	71	71	71	71	66	67	68	68	67	68	69	67	67	67	67	67	65
	Methylomonas koyamae R-49807	*Methylomonas koyamae* R-49807	18	18	18	18	18	19	19	18	20	19	20	19	18	20	20	19	18	19	18	19	19	19	17	17	18	17	19	20	20	18	17	19	17	17	26	34	33	35	20	19	28	17	18	18	18	20	17	22	22	22	22	22	22	21	21	92		97	97	95	76	76	73	73	73	73	73	72	73	72	71	69	69	70	71	71	71	70	66	67	68	68	67	68	69	67	67	67	67	66	65
	Methylomonas sp. DH-1	*Methylomonas koyamae* DH-1	19	19	19	18	18	20	20	18	20	19	20	19	18	20	20	19	18	19	18	19	20	19	18	17	18	17	18	18	20	18	21	21	17	18	26	34	32	34	19	19	30	19	18	17	19	20	17	21	21	21	21	22	22	21	21	76	76		97	95	76	76	73	73	73	73	73	71	73	72	71	69	69	70	70	71	71	70	66	67	68	68	67	68	69	67	67	67	67	66	66
	Methylomonas koyamae JCM 16701^{T}	*Methylomonas koyamae* JCM 16701^{T}	18	18	18	18	18	19	19	18	19	19	19	18	19	19	19	18	18	19	18	20	19	19	17	17	18	17	18	20	19	18	17	20	16	19	26	34	34	39	19	18	28	17	17	17	18	19	17	21	21	21	21	22	22	21	21	73	72	74		95	76	76	73	73	73	73	74	71	73	72	71	69	69	70	71	71	71	71	66	67	68	68	67	68	69	67	67	67	67	67	65
	Methylomonas koyamae R-45378	*Methylomonas* sp. R-45378	18	18	18	18	18	20	20	19	20	19	19	19	17	19	19	19	18	20	18	19	18	19	17	17	17	17	18	20	19	18	17	20	17	17	26	34	23	36	20	18	28	18	18	20	18	21	18	21	21	21	22	22	22	21	21	65	64	65	64		76	76	73	73	73	73	73	71	74	72	71	69	69	70	71	71	71	70	66	67	67	68	67	68	69	67	67	66	67	66	66
	Methylomonas koyamae R-45383	*Methylomonas* sp. R-45383	18	18	19	18	19	20	20	19	20	19	19	18	19	20	20	20	20	22	19	21	19	21	18	18	18	18	18	20	19	18	24	19	16	18	22	22	18	36	20	19	35	19	17	17	19	18	18	21	21	22	22	22	22	22	21	22	22	22	22	22		97	73	73	73	73	73	71	73	72	71	69	69	70	71	71	71	70	66	67	67	67	67	68	68	67	67	67	66	66	65
	Methylomonas sp. LWB	*Methylomonas* sp. LWB	18	18	20	18	19	21	21	18	22	21	20	20	18	21	21	21	21	22	18	20	22	23	18	18	17	17	18	19	22	18	30	22	17	19	25	25	19	32	20	23	34	19	18	17	18	18	18	22	21	21	22	22	22	21	21	22	22	22	22	22	77		73	73	73	73	73	71	73	72	71	69	69	70	71	71	71	70	66	67	68	68	68	68	69	67	67	67	67	67	66
	Methylomonas sp. BC.006	*Methylomonas* sp. BC.006	19	19	18	19	19	18	18	18	19	18	19	20	18	17	17	17	17	18	18	19	16	16	20	19	18	20	19	18	17	19	16	18	18	18	52	24	30	60	17	17	22	17	[illegible]	17	17	17	16	19	19	19	19	19	19	19	19	20	20	20	20	21	20	19		100	100	99	100	71	72	72	70	68	68	69	70	70	70	70	66	67	68	68	67	68	69	67	67	66	67	66	66
	Methylomonas sp. DS2.3.37	*Methylomonas* sp. DS2.3.37	19	18	17	19	18	18	18	18	18	18	18	20	17	17	17	17	17	17	17	18	16	16	19	19	18	17	19	17	18	19	16	18	18	18	22	21	26	60	17	16	23	17	17	17	17	17	17	18	19	19	19	19	19	19	19	20	20	20	20	21	20	19	100		100	99	100	71	72	72	70	68	68	69	70	70	70	70	66	67	68	68	67	68	69	67	67	66	67	66	66
	Methylomonas sp. DS1.3.54	*Methylomonas* sp. DS1.3.54	19	18	17	19	19	18	18	18	18	18	18	20	17	17	17	17	17	18	17	18	16	16	19	19	18	17	18	17	17	18	16	18	18	21	22	21	26	60	78	16	22	17	17	16	17	16	16	19	19	19	19	19	19	19	19	20	20	20	20	20	20	19	100	100		99	100	71	72	72	70	68	68	69	70	70	70	70	66	67	68	68	67	68	69	67	67	66	67	66	66
	Methylomonas sp. DS1.022	*Methylomonas* sp. DS1.022	19	18	18	19	18	18	18	18	18	18	18	19	17	17	17	17	17	18	17	18	16	16	19	20	18	17	18	17	17	18	17	17	18	15	22	21	26	60	78	16	45	17	18	70	71	63	68	19	19	19	19	19	19	20	20	20	20	20	20	21	20	19	100	100	100		99	70	72	72	70	68	68	69	70	70	70	70	66	67	68	68	67	68	69	67	67	66	67	66	66
	Methylomonas sp. FW.001	*Methylomonas* sp. FW.001	18	18	17	19	18	18	18	18	18	18	18	20	17	17	17	17	17	17	17	18	16	16	18	19	18	17	19	17	18	19	16	17	18	15	22	21	27	60	19	16	22	17	17	17	18	17	16	18	19	19	19	19	19	19	19	20	20	20	20	20	20	19	100	100	100	100		71	72	72	70	68	68	69	70	70	70	70	66	67	68	67	67	68	68	67	67	67	67	66	66
	Methylomonas lenta R-45370	*Methylomonas lenta* R-45370	20	19	19	19	19	20	21	19	21	19	20	18	18	25	25	20	21	20	17	18	20	21	17	17	17	18	18	17	19	18	20	19	18	42	17	25	20	41	21	18	23	19	17	20	16	18	16	21	21	20	21	20	19	20	20	20	19	19	19	19	20	20	18	18	18	18	18		76	75	69	69	69	69	68	68	68	68	65	65	65	65	65	65	66	65	65	65	65	65	65
	Methylomonas methanica MC09	*Methylomonas* sp. MC09	17	17	19	18	18	19	19	18	19	19	19	20	17	20	20	20	20	19	18	19	18	18	18	17	17	16	17	17	21	17	22	20	18	17	28	29	33	35	18	19	23	17	17	17	18	18	19	20	20	21	21	20	20	21	21	20	20	20	20	20	20	21	19	19	19	19	19	21		75	71	69	69	70	70	70	70	70	65	66	67	67	66	67	68	66	66	66	66	66	65
	Methylomonas sp. FW.007	*Methylomonas* sp. FW.007	18	18	18	17	17	19	18	17	18	18	18	17	17	19	19	18	18	19	17	18	18	18	17	17	17	17	18	18	17	18	18	16	17	16	24	14	31	57	55	17	19	17	18	17	18	19	18	22	22	21	21	21	21	21	21	20	20	20	19	20	20	21	18	18	18	18	18	20	20		70	68	68	69	68	68	69	69	65	66	65	65	65	66	66	65	65	65	66	65	65
	Methylomarinum vadi IT-4^{T}	*Methylomarinum vadi* IT-4^{T}	17	17	20	17	17	19	18	18	19	19	18	18	17	20	20	20	19	20	17	19	18	19	17	17	16	18	18	17	18	18	22	22	18	16	28	27	34	38	26	21	23	18	18	16	16	16	16	20	20	20	19	19	19	19	19	19	19	19	19	19	20	20	18	18	18	18	18	20	19	18		69	69	70	70	70	71	70	65	66	67	67	67	67	67	66	66	66	66	66	65
	Methylomicrobium alcaliphilum 20Z^{T}	*Methylotuvimicrobium alcaliphilum* 20Z^{T}	18	18	20	19	19	20	21	19	21	20	20	19	18	22	22	20	20	20	18	20	19	19	18	19	18	17	17	17	19	17	18	21	18	16	18	28	22	23	16	21	24	17	17	18	16	18	17	22	23	20	20	19	19	20	20	19	20	19	20	20	20	22	19	19	18	19	18	21	20	21	19		95	86	70	70	70	69	65	66	66	66	65	66	66	66	66	65	65	65	65
	"*Methylomicrobium buryatense*" 5G	"*Methylotuvimicrobium buryatense*" 5G	18	18	19	19	19	20	20	19	20	19	23	19	18	21	21	19	19	20	18	20	21	22	19	17	17	17	18	17	19	17	18	22	19	16	23	20	22	35	15	19	22	18	18	17	16	18	16	23	20	20	21	19	20	20	20	20	20	19	20	21	21	22	18	18	18	18	18	21	21	19	19	63		86	69	70	70	70	65	66	66	66	65	66	66	66	66	65	66	65	65
	Methylomicrobium kenyense AMO1^{T}	*Methylotuvimicrobium kenyense* AMO1^{T}	18	18	19	18	19	20	20	18	20	19	20	16	17	20	20	20	19	19	18	22	20	20	19	19	18	17	18	17	20	18	20	22	18	17	21	22	26	38	16	21	22	17	17	17	17	18	17	19	21	20	21	19	20	19	19	20	21	20	20	20	20	21	19	19	18	18	18	23	22	19	19	33	33		71	71	71	71	65	67	67	67	66	67	67	66	66	66	66	66	65
	Methylomicrobium agile ATCC 35068^{T}	*Methylomicrobium agile* ATCC 35068^{T}	18	18	19	18	19	19	20	18	19	19	18	19	17	21	21	20	20	20	19	20	20	21	18	19	18	16	17	17	18	17	21	20	19	15	24	27	21	26	17	19	32	17	18	17	17	18	17	19	19	18	20	19	19	20	20	19	19	19	19	20	20	20	18	18	18	18	18	21	20	20	20	19	19	20		99	81	75	66	68	68	68	68	68	69	68	67	67	67	67	66
	Methylomicrobium album BG8^{T}	*Methylomicrobium album* BG8^{T}*	19	19	19	19	18	19	19	18	19	19	18	19	17	20	20	20	20	20	18	20	19	20	18	19	18	16	17	16	17	16	20	19	19	15	23	26	20	34	17	18	30	17	18	17	17	18	18	19	19	19	21	19	19	20	20	19	19	19	19	19	19	20	18	18	18	18	18	21	20	19	20	19	19	20	90		81	75	66	67	68	68	68	68	69	68	67	67	67	67	65
	Methylosarcina lacus LW14^{T}	*Methylosarcina lacus* LW14^{T}	20	20	21	20	19	20	20	20	21	19	20	18	19	20	20	19	19	20	18	21	19	19	19	17	17	16	17	17	18	17	20	20	18	15	26	28	27	36	17	18	27	17	19	18	18	18	18	20	20	20	19	19	20	20	19	19	19	19	19	19	19	20	18	17	17	17	17	22	20	19	19	19	19	20	26	26		75	66	68	68	68	68	68	69	67	67	67	67	67	66
	Methylosarcina fibrata AML-C10^{T}	*Methylosarcina fibrata* AML-C10^{T}	19	18	19	19	19	20	20	20	19	18	19	18	20	20	20	23	20	19	20	20	20	[illegible]	[illegible]	18	19	18	17	18	18	18	18	20	18	17	30	25	26	42	13	19	19	17	19	18	17	18	18	19	19	20	20	19	18	20	19	20	19	19	19	18	19	19	18	18	18	18	18	22	21	18	19	19	19	21	23	23	21		66	67	68	68	68	68	69	67	67	67	67	67	66
	Methylococcaceae bacterium UBA1147	*Methylococcaceae* bacterium UBA1147	17	17	33	27	31	21	19	23	18	24	28	27	31	17	17	16	16	17	16	17	17	17	17	25	28	21	25	24	32	27	16	21	19	28	19	21	26	50	31	37	36	15	16	15	15	15	15	16	17	24	33	32	33	18	17	32	32	21	20	32	19	21	19	18	18	19	19	18	24	17	22	18	19	20	19	18	20	17		67	66	66	66	66	66	66	66	65	66	65	65
	Methylococcaceae bacterium UBA2778	*Methylococcaceae* bacterium UBA2778	17	17	17	16	16	16	17	16	16	17	47	28	25	18	18	17	17	15	20	18	17	18	16	17	24	54	53	58	47	23	15	14	16	50	20	24	47	21	19	20	45	28	20	15	15	15	14	14	16	15	16	15	15	15	15	16	15	16	17	16	19	18	18	18	18	18	18	47	17	15	19	15	15	16	17	17	16	16	18		67	67	67	67	68	67	67	67	66	66	66
	Methylococcus capsulatus Bath	*Methylococcus capsulatus* Bath	15	16	19	16	19	23	23	16	23	19	35	21	18	22	22	20	19	21	16	21	21	22	21	17	16	18	23	15	20	17	32	33	25	23	18	24	22	24	18	21	29	21	18	15	16	16	16	25	24	28	23	22	21	22	20	19	18	19	19	19	19	21	17	17	17	17	16	19	24	16	22	27	28	24	19	20	20	19	17	16		99	71	71	71	70	70	70	69	68	66
	Methylococcus capsulatus Texas^{T}	*Methylococcus capsulatus* Texas^{T}	15	16	23	16	20	25	25	17	25	21	39	26	19	23	23	22	21	22	16	22	24	25	21	16	16	18	25	15	23	17	33	35	25	25	18	24	22	23	18	23	30	22	18	16	16	16	16	27	24	28	28	23	23	23	21	20	19	20	20	20	21	22	17	17	16	16	16	21	26	16	21	29	30	26	21	21	21	20	18	16	91		71	71	71	71	70	70	69	68	66
	Methyloterricola oryzae 73a^{T}	*Methyloterricola oryzae* 73a^{T}	17	16	18	16	17	23	23	16	23	23	32	49	72	22	22	21	19	20	17	20	19	21	25	17	17	25	24	15	19	18	21	33	25	21	21	25	16	18	16	20	31	18	16	16	15	16	16	20	21	23	22	19	20	20	18	18	18	18	17	17	20	22	17	17	17	17	17	25	20	18	22	19	22	22	19	19	20	17	17	16	19	19		71	71	70	70	69	69	68	66
	Methylomagnum ishizawai 175	*Methylomagnum ishizawai* 175	16	16	19	17	18	20	20	17	20	17	47	20	59	19	19	19	18	18	17	21	20	21	16	17	17	22	26	15	21	18	22	31	30	16	20	27	21	21	16	22	28	19	16	16	16	17	17	18	21	20	22	19	19	18	17	20	18	19	18	19	18	19	17	17	17	17	17	20	21	17	20	24	25	21	19	19	19	19	16	17	19	19	19		71	71	71	71	70	68	66
	Methylogaea oryzae JCM 16910^{T}	*Methylogaea oryzae* JCM 16910^{T}	17	17	19	16	18	21	21	16	23	19	24	23	21	20	20	19	18	21	16	19	18	19	18	17	17	24	25	16	20	18	20	24	19	28	20	25	25	23	25	18	32	18	16	17	17	17	17	19	19	20	20	18	18	19	18	19	19	19	18	19	19	21	17	17	17	17	17	19	20	16	20	25	25	25	20	19	21	18	17	17	20	21	20	19		70	70	69	69	68	67
	Methylocaldum sp. 14B	*Methylocaldum* sp. 14B	20	20	22	17	17	21	23	18	22	19	17	32	16	21	21	22	20	22	16	21	19	20	16	18	18	20	23	14	18	18	21	31	17	16	19	22	14	21	15	18	32	18	18	17	15	18	18	19	20	23	22	19	19	22	20	19	18	18	19	18	18	20	18	18	18	17	18	20	24	16	20	20	20	20	21	21	19	19	15	16	19	22	18	18	19		99	81	69	67	66
	Methylocaldum sp. SAD2	*Methylocaldum* sp. SAD2	20	20	21	18	16	17	17	19	19	17	14	18	15	19	19	20	19	20	17	16	15	15	16	18	17	20	21	15	14	18	15	22	17	16	19	19	14	17	15	16	20	17	19	16	15	17	17	18	17	16	17	17	17	18	18	17	17	17	17	17	16	17	16	16	16	16	16	18	18	17	17	15	15	16	18	19	18	16	16	16	18	18	18	18	18	91		81	69	67	66
	Methylocaldum szegediense O-12	*Methylocaldum szegediense* O-12	20	20	22	16	19	21	21	18	21	18	41	20	19	23	23	22	19	21	16	21	19	21	17	22	17	17	19	15	17	18	22	28	17	16	24	24	15	19	15	17	28	18	19	16	18	18	17	21	26	23	20	19	18	26	24	18	17	18	17	18	21	17	17	17	17	17	17	21	21	17	22	20	20	19	21	21	20	20	17	17	19	20	19	18	19	25	25		69	67	66
	Methylococcus sp. UBA6136	*Methylococcaceae* bacterium UBA6136	17	17	17	18	17	17	16	17	17	16	47	19	28	16	16	17	16	18	16	17	17	17	17	16	18	16	18	15	15	16	15	21	33	20	26	30	20	17	16	15	24	17	17	16	15	16	17	17	19	16	16	17	17	17	17	17	16	16	16	16	20	17	16	16	16	16	16	15	17	16	18	17	17	16	17	17	16	17	15	17	17	17	18	18	18	16	16	16		67	66
	Methylohalobius crimeensis 10Ki^{T}	*Methylohalobius crimeensis* 10Ki^{T}	16	17	19	15	17	20	20	16	20	19	26	18	14	19	19	19	18	19	15	23	21	22	24	15	15	21	25	15	20	17	22	29	24	18	30	21	19	24	18	19	25	19	16	15	15	16	15	21	22	22	23	23	22	20	19	20	19	19	20	18	18	21	15	15	16	16	16	20	21	15	18	22	23	21	19	18	20	18	16	16	19	20	18	17	19	18	16	19	16		70
	Methylothermaceae bacterium B42	*Methylothermaceae* bacterium B42	15	15	18	17	19	19	20	15	20	17	39	17	15	21	21	22	19	22	15	20	17	18	18	16	17	14	15	15	20	17	20	29	17	18	20	20	23	21	19	19	26	17	16	20	19	25	21	25	18	24	21	18	19	19	18	19	17	17	18	18	24	25	15	16	15	15	15	18	17	15	23	21	23	20	20	20	18	18	16	16	17	17	18	18	18	18	17	20	16	17	

Values are shown for dDDH (yellow–red, lower left) and ANI (green–blue, upper right). Boxed regions indicate inferred species clusters with at least two members based on comparisons that are at least 70% dDDH and 95% ANI, as well as monophyly in the genome-based phylogeny. Asterisks after reclassified names indicate later heterotypic synonyms of their senior counterparts in the same species clusters as shown in Figure 2.

Supplementary Table S5. Reclassification of the *Methylococcales* genomes.

Previous name	Name after reclassification	Genus cluster	Species cluster	G+C of DNA (mol%)	Genome size (bp)
Crenothrix polyspora RSM_CP1	*Crenothrix polyspora* RSM_CP1	6	20	44.24	3,921,762
Crenothrix polyspora RSM_CP2	*Crenothrix* sp. RSM_CP2	6	21	44.73	3,527,685
Crenothrix sp. D3	*Methylococcaceae* bacterium D3	4	17	42.18	3,633,770
Methylobacter luteus IMV-B-3098	*Methylobacter luteus* IMV-B-3098	1	10	51.13	5,027,778
Methylobacter marinus A45^{T}	*Methylobacter marinus* A45^{T}	1	9	52.67	4,987,856
Methylobacter sp. BBA5.1	*Methylobacter marinus* BBA5.1	1	9	52.24	5,071,908
Methylobacter sp. BC.3.60	*Methylococcaceae bacterium* BC.3.60	7	22	45.33	1,321,518
Methylobacter sp. BC.3.94	*Methylovulum miyakonense* BC.3.94	2	11	50.52	4,467,756
Methylobacter sp. DS2.3.46	*Methylococcaceae* bacterium DS2.3.46	15	32	48.79	3,075,382
Methylobacter sp. DS3.3.25	*Methylococcaceae* bacterium DS3.3.25	15	32	49.80	2,753,434
Methylobacter sp. UBA2091	*Methylobacter* sp. UBA2091	1	1	49.28	3,970,437
Methylobacter sp. UBA6696	*Methylobacter* sp. UBA6696	1	1	49.27	3,961,971
Methylobacter sp. UBA6701	*Methylobacter* sp. UBA6701	1	2	49.26	3,793,708
Methylobacter sp. UBA6712	*Methylobacter* sp. UBA6712	1	2	48.86	4,212,994
Methylobacter sp. WM.3.3	*Methylococcaceae* bacterium WM.3.3	15	32	49.39	2,673,132
Methylobacter tundripaludum 21/22	*Methylobacter* sp. 21/22	1	3	49.46	4,665,210
Methylobacter tundripaludum 31/32	*Methylobacter* sp. 31/32	1	3	49.24	5,047,546
Methylobacter tundripaludum OWC-DMM	*Methylobacter* sp. OWC-DMM	1	1	49.47	4,607,785
Methylobacter tundripaludum OWC-G53F	*Methylobacter* sp. OWC-G53F	1	6	50.46	4,180,651
Methylobacter tundripaludum SV96^{T}	*Methylobacter tundripaludum* SV96^{T}	1	5	49.51	4,846,919
Methylobacter tundripaludum UBA6706	*Methylobacter* sp. UBA6706	1	4	49.44	3,565,077
Methylobacter whittenburyi ACM 3310^{T}	*Methylobacter whittenburyi* ACM 3310^{T}*	1	9	51.98	5,440,158
Methylobacter whittenburyi UCM-B-3033	*Methylobacter marinus* UCM-B-3033	1	9	51.98	5,440,158
Methylocaldum sp. 14B	*Methylocaldum* sp. 14B	27	59	58.24	5,621,438
Methylocaldum sp. SAD2	*Methylocaldum* sp. SAD2	27	59	58.18	5,907,411
Methylocaldum szegediense O-12	*Methylocaldum szegediense* O-12	27	60	57.15	5,011,695
Methylococcaceae bacterium NSM2-1	*Methylobacter* sp. NSM2-1	1	8	44.82	1,901,400
Methylococcaceae bacterium NSO1	*Methylobacter* sp. NSO1	1	7	43.92	1,404,415
Methylococcaceae bacterium NSP1-1	*Methylobacter* sp. NSP1-1	1	7	44.25	1,667,819
Methylococcaceae bacterium NSP1-2	*Methylococcaceae* bacterium NSP1-2	4	18	42.81	2,718,667
Methylococcaceae bacterium TMED282	*Methylococcaceae* bacterium TMED282	10	26	43.73	1,499,574
Methylococcaceae bacterium TMED69	*Methylococcaceae* bacterium TMED69	11	27	38.55	1,973,107
Methylococcaceae bacterium UBA1114	*Methylococcaceae* bacterium UBA1114	8	23	42.83	2,173,118

Previous name	Name after reclassification	Genus cluster	Species cluster	G+C of DNA (mol%)	Genome size (bp)
Methylococcaceae bacterium UBA1147	*Methylococcaceae* bacterium UBA1147	21	53	53.15	2,103,507
Methylococcaceae bacterium UBA2778	*Methylococcaceae* bacterium UBA2778	22	54	55.65	3,927,269
Methylococcaceae bacterium UBA2780	*Methylococcaceae* bacterium UBA2780	9	25	44.90	2,373,821
Methylococcaceae bacterium UBA3127	*Methylococcaceae* bacterium UBA3127	4	16	42.18	3,099,008
Methylococcaceae bacterium UBA4132	*Methylococcaceae* bacterium UBA4132	4	16	42.15	3,069,361
Methylococcaceae bacterium UBA5071	*Methylococcaceae* bacterium UBA5071	4	16	42.15	3,132,147
Methylococcaceae bacterium UBA6146	*Methylovulum* sp. UBA6146	2	13	48.50	3,187,382
Methylococcaceae bacterium UBA659	*Methylococcaceae* bacterium UBA659	3	14	51.40	2,140,836
Methylococcaceae bacterium UBA662	*Methylococcaceae* bacterium UBA662	3	15	52.99	2,387,527
Methylococcaceae bacterium UBA7658	*Methyloglobulus* sp. UBA7658	14	30	46.81	3,709,136
Methylococcaceae bacterium UBA975	*Methylococcaceae* bacterium UBA975	8	24	44.76	2,328,141
Methylococcales bacterium OPU3_GD_OMZ	*Methylococcaceae* bacterium OPU3_GD_OMZ	12	28	42.32	2,094,096
Methylococcus capsulatus Bath	*Methylococcus capsulatus* Bath	23	55	63.58	3,304,560
Methylococcus capsulatus Texas^{T}	*Methylococcus capsulatus* Texas^{T}	23	55	63.56	3,263,814
Methylococcus sp. UBA6136	*Methylococcaceae* bacterium UBA6136	28	61	57.14	2,440,517
"*Methylocucumis oryzae*" Sn10-6^{T}	"*Methylocucumis oryzae*" Sn10-6^{T}	5	19	43.87	4,583,004
Methylogaea oryzae JCM 16910^{T}	*Methylogaea oryzae* JCM 16910^{T}	26	58	62.55	3,696,254
Methyloglobulus morosus KoM1^{T}	*Methyloglobulus morosus* KoM1^{T}	14	31	47.32	4,136,844
Methylohalobius crimeensis 10Ki^{T}	*Methylohalobius crimeensis* 10Ki^{T}	29	62	58.28	3,509,607
Methylomagnum ishizawai 175	*Methylomagnum ishizawai* 175	25	57	62.96	5,480,099
Methylomarinum vadi IT-4^{T}	*Methylomarinum vadi* IT-4^{T}	17	46	51.29	4,335,162
Methylomicrobium agile ATCC 35068^{T}	*Methylomicrobium agile* ATCC 35068^{T}	19	50	56.17	4,526,071
Methylomicrobium album BG8^{T}	*Methylomicrobium album* BG8^{T}*	19	50	56.25	4,493,344
Methylomicrobium alcaliphilum 20Z^{T}	*Methylotuvimicrobium alcaliphilum* 20Z^{T}	18	47	48.74	4,796,711
"*Methylomicrobium buryatense*" 5G	"*Methylotuvimicrobium buryatense*" 5G	18	48	48.71	5,064,796
Methylomicrobium kenyense AMO1^{T}	*Methylotuvimicrobium kenyense* AMO1^{T}	18	49	50.16	4,474,416
"*Methylomonas denitrificans*" FJG1^{T}	"*Methylomonas denitrificans*" FJG1^{T}*	16	38	51.66	5,172,098
Methylomonas koyamae JCM 16701^{T}	*Methylomonas koyamae* JCM 16701^{T}	16	39	56.05	4,954,151
Methylomonas koyamae LM6	*Methylomonas koyamae* LM6	16	39	56.23	5,080,660
Methylomonas koyamae R-45378	*Methylomonas* sp. R-45378	16	40	56.06	5,115,236
Methylomonas koyamae R-45383	*Methylomonas* sp. R-45383	16	41	55.85	5,414,320
Methylomonas koyamae R-49807	*Methylomonas koyamae* R-49807	16	39	55.86	5,179,245
Methylomonas lenta R-45370	*Methylomonas lenta* R-45370	16	43	46.63	4,703,858
Methylomonas methanica MC09	*Methylomonas* sp. MC09	16	44	51.31	5,051,681
Methylomonas methanica NCIMB 11130^{T}	*Methylomonas methanica* NCIMB 11130^{T}	16	38	51.67	5,023,270

Previous name	Name after reclassification	Genus cluster	Species cluster	G+C of DNA (mol%)	Genome size (bp)
Methylomonas methanica R-45363	*Methylomonas* sp. R-45363	16	33	51.29	5,407,230
Methylomonas methanica R-45371	*Methylomonas* sp. R-45371	16	34	51.27	5,482,877
Methylomonas sp. 11b	*Methylomonas* sp. 11b	16	35	51.40	5,412,616
Methylomonas sp. BC.006	*Methylomonas* sp. BC.006	16	42	56.11	2,569,055
Methylomonas sp. DH-1	*Methylomonas koyamae* DH-1	16	39	56.21	5,127,407
Methylomonas sp. DS1.022	*Methylomonas* sp. DS1.022	16	42	56.48	2,850,821
Methylomonas sp. DS1.3.54	*Methylomonas* sp. DS1.3.54	16	42	56.50	2,478,482
Methylomonas sp. DS2.3.37	*Methylomonas* sp. DS2.3.37	16	42	56.14	2,570,809
Methylomonas sp. FW.001	*Methylomonas* sp. FW.001	16	42	55.96	2,324,165
Methylomonas sp. FW.007	*Methylomonas* sp. FW.007	16	45	48.36	4,032,484
Methylomonas sp. FW.017	*Methylococcaceae* bacterium FW.017	15	32	49.21	2,488,664
Methylomonas sp. Kb3	*Methylomonas* sp. Kb3	16	37	51.81	5,121,132
Methylomonas sp. LW13	*Methylomonas* sp. LW13	16	37	51.80	5,200,804
Methylomonas sp. LWB	*Methylomonas* sp. LWB	16	41	55.93	5,365,240
Methylomonas sp. MK1	*Methylomonas* sp. MK1	16	36	51.47	5,233,591
Methyloprofundus sedimenti WF1^T	*Methyloprofundus sedimenti* WF1^T	13	29	41.02	4,290,526
Methylosarcina fibrata AML-C10^T	*Methylosarcina fibrata* AML-C10^T	20	52	54.07	4,978,052
Methylosarcina lacus LW14^T	*Methylomicrobium lacus* LW14^T	19	51	54.67	4,384,919
Methyloterricola oryzae 73a^T	*Methyloterricola oryzae* 73a^T	24	56	61.10	4,902,365
Methylothermaceae bacterium B42	*Methylothermaceae* bacterium B42	30	63	51.47	3,040,036
Methylovulum miyakonense HT12^T	*Methylovulum miyakonense* HT12^T	2	11	50.66	4,686,311
Methylovulum psychrotolerans HV10-M2	*Methylovulum psychrotolerans* HV10-M2	2	12	50.88	4,923,391
Methylovulum psychrotolerans Sph1^T	*Methylovulum psychrotolerans* Sph1^T	2	12	50.85	5,162,352

Asterisks after reclassified names indicate later heterotypic synonyms of their senior counterparts in the same species clusters.

2. Supplementary Figures

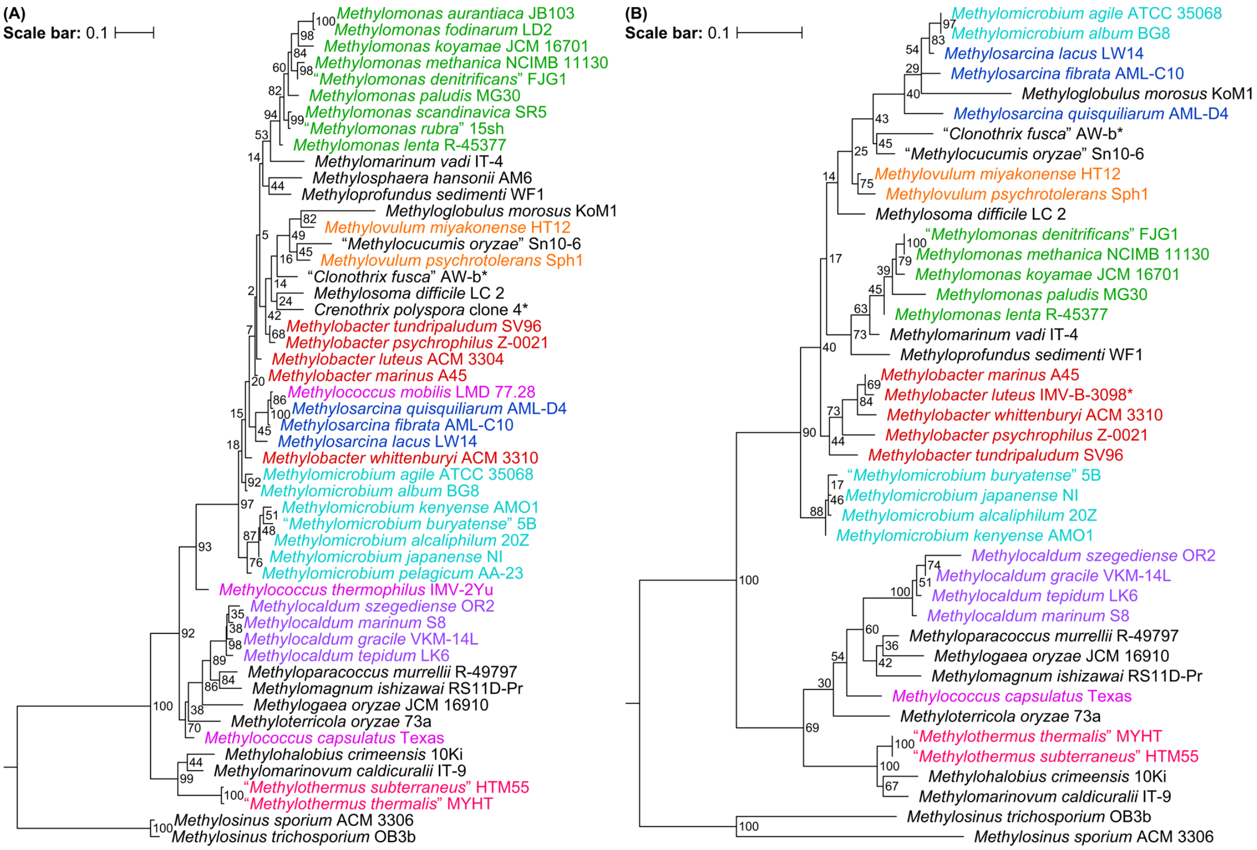

Supplementary Figure S1. The phylogenetic relationship of *Methylococcales* type and representative strains based on (A) 16S rRNA and (B) PmoA sequences. The phylogenetic trees are the same trees as in Figure 1 but expanded to show all nodes and bootstrap support.

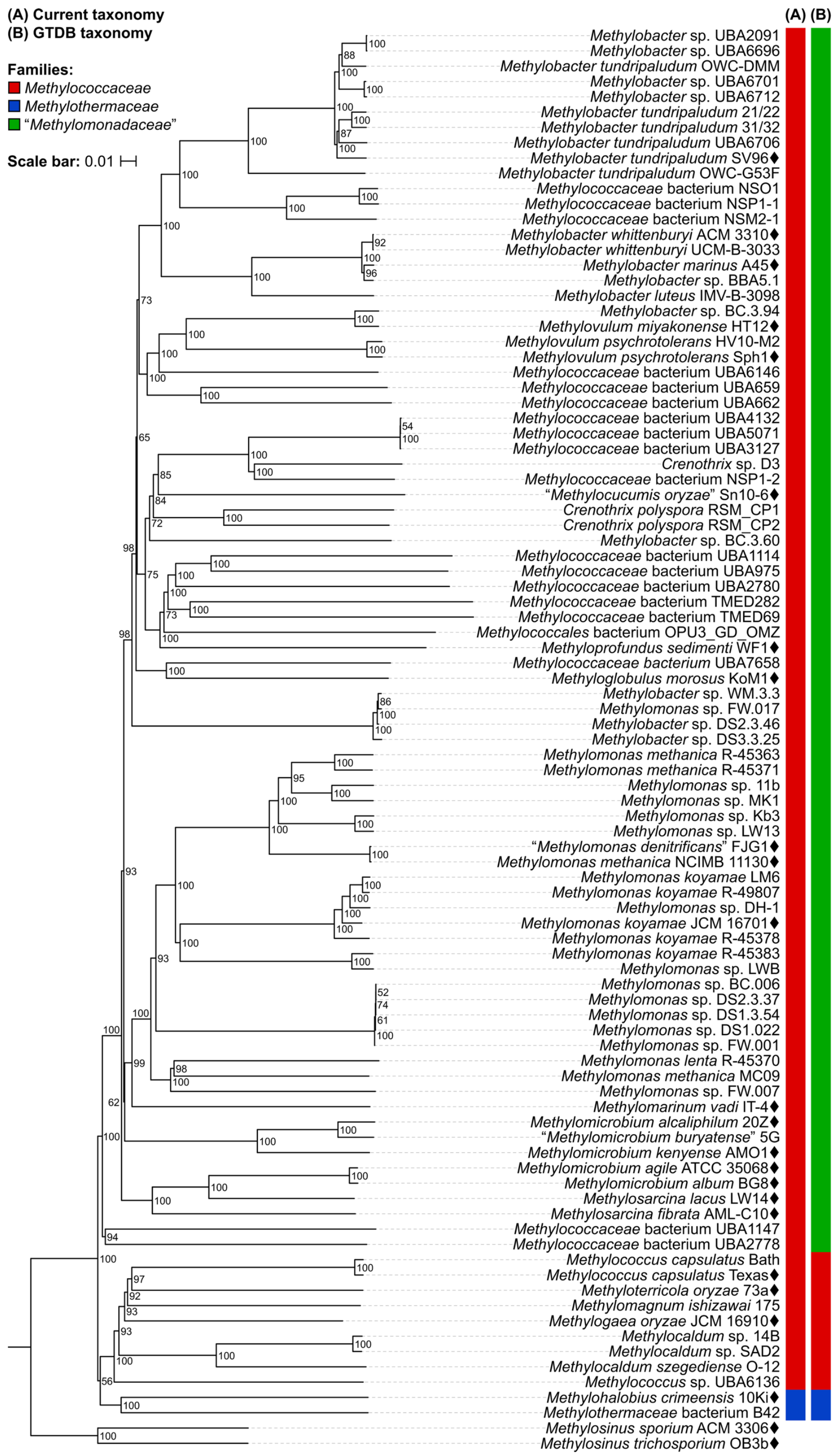

Supplementary Figure S2. The phylogenetic relationship of the *Methylococcales* genomes and their family designations. The phylogenetic tree is the same tree as in Figure 2. (A) The genus *Crenothrix* is listed under family *Methylococcaceae* in the *Bergey's Manual of Systematics of Archaea and Bacteria* (Bowman, 2016) and the Genome Taxonomy Database (GTDB) (Parks et al., 2018) but is listed under *Crenotrichaceae* in the List of Prokaryotic Names with Standing in Nomenclature (Parte, 2014). (B) GTDB taxonomy proposed family-level reclassification.

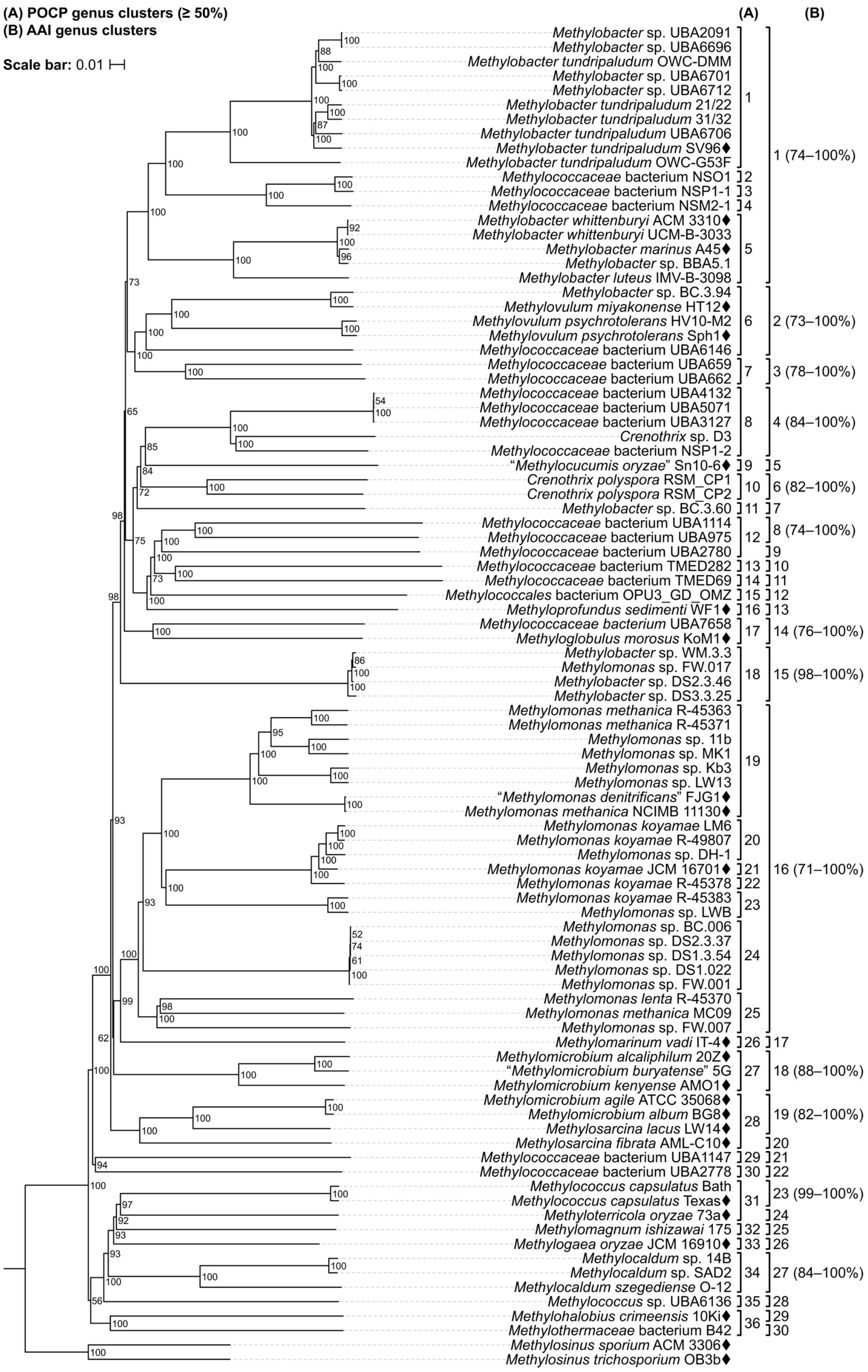

Supplementary Figure S3. Inferred genus clusters based on (A) POCP and (B) AAI. The phylogenetic tree is the same tree as in Figure 2. (A) Inferred genus clusters based on monophyly and the 50% POCP threshold for genus delineation (Qin et al., 2014). (B) AAI range values are indicated for every inferred genus cluster based on monophyly and AAI.

3. Supplementary References

Bowman, J.P. (2016). "*Methylococcaceae*," in *Bergey's Manual of Systematics of Archaea and Bacteria*, eds. W.B. Whitman, F. Rainey, P. Kämpfer, M. Trujillo, J. Chun, P. De Vos, B. Hedlund & S. Dedysh. (Hoboken, NJ: John Wiley & Sons, Inc.).

Parks, D.H., Chuvochina, M., Waite, D.W., Rinke, C., Skarshewski, A., Chaumeil, P.A., et al. (2018). A standardized bacterial taxonomy based on genome phylogeny substantially revises the tree of life. *Nat. Biotechnol.* 36, 996-1004. doi: 10.1038/nbt.4229.

Parte, A.C. (2014). LPSN—list of prokaryotic names with standing in nomenclature. *Nucleic Acids Res.* 42, D613-D616. doi: 10.1093/nar/gkt1111.

Qin, Q.L., Xie, B.B., Zhang, X.Y., Chen, X.L., Zhou, B.C., Zhou, J., et al. (2014). A proposed genus boundary for the prokaryotes based on genomic insights. *J. Bacteriol.* 196, 2210-2215. doi: 10.1128/JB.01688-14.
